# Stereogenic-at-Metal Ir(III) Complexes as Platforms for the Construction of Asymmetric Bimetallic Complexes

**DOI:** 10.1021/acs.inorgchem.5c04469

**Published:** 2025-12-09

**Authors:** Paul D. Newman, James A. Platts, Simon J. A. Pope, Benson M. Kariuki

**Affiliations:** School of Chemistry, 2112 Cardiff University, Park Place, Cardiff, Wales CF10 3AT, U.K.

## Abstract

The stereochemical-at-metal complexes Δ- and Λ-[Ir­(ppy)_2_(^*S,R*^ **L**)]­BF_4_, where ^*S,R*^ **L** is a bpy ligand substituted at the 6-position with a chiral diamine or derivative thereof, have been prepared and fully characterized by a combination of empirical and theoretical methods. The differing solubility of the Δ and Λ parent diastereomers in acetone allowed their ready separation. Further functionalization of an external amino group occurred with complete retention of absolute configuration and gave systems capable of binding to a second metal. The facility to coordinate other metals was explored by using in situ ^1^H NMR experiments with ZnI_2_. Coordination shifts were seen in the ^1^H NMR spectra, confirming the coordination of Zn­(II) and showcasing the potential of the complexes as flexible platforms for the construction of asymmetric bimetallics.

## Graphical abstract

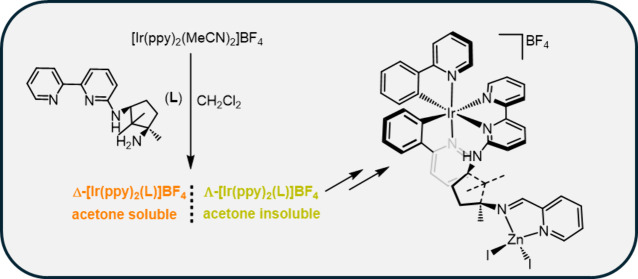

## Introduction

Given the nature of living systems, the concept of chirality and the control of stereoselectivity have been, and continue to be, fascinating to chemists. The pioneering work of Werner^1^ alerted the chemistry fraternity to the concept of chirality in coordination compounds, and although relatively dormant for decades, renewed interest in stereogenic-at-metal (SAM) complexes has arisen due to their widening importance and applicability in medicinal chemistry,^2–5^ materials science,^6,7^ and catalysis.^8–12^ The general term stereogenic-at-metal (SAM) refers to complexes where the metal is not necessarily the sole source of chirality and includes the subset where metal stereochemistry is combined with ligand-based chirality. The early coordination compounds of Werner where the stereogenicity is exclusively at the metal are called chiral-at-metal (CAM) complexes, and research in this area has been reignited by the elegant work of the groups of Meggers,^13–17^ Davies,^18^ Gladysz,^19^ Grubbs,^10^ and others^20–27^ showcasing examples of tetrahedral^20^ or pseudotetrahedral (piano-stool),^21,28–30^ five-coordinate, and octahedral CAM complexes.^2–9,2–9,13–17^

There are numerous things to consider when designing chiral (stereogenic)-at-metal complexes for specific applications. Inert metal ions are often preferred, as they tend to be stereochemically robust and hence maintain their structural integrity under the reaction conditions necessary for employment. However, if the application requires substitution within the coordination sphere, then labile sites are needed even with typically inert metal ions. Enabling control in inherently labile systems is more difficult, and although there are examples of this using mono- (albeit rarely) or bidentate ligands,^11^ multidentate ligands are usually necessary to ensure maintenance of configurational integrity. Examples of CAM complexes of labile metal ions that do not readily undergo ligand redistribution and/or racemization are rare but not unknown.^20,26^

We are mainly interested in diastereomeric complexes where a chiral-metal center is combined with a second chiral element in one or more bound ligands. The inclusion of an asymmetric ligand is judicious, as it often leads to stereoselective formation of a single diastereomer when generating a chiral metal center upon coordination. Even when selective formation of the desired SAM complex proves elusive, ligands with a prebuilt chiral element(s) are advantageous as they can enable the ready separation of the resultant diastereomeric mixture; this has been exploited by Meggers for the recovery of pure chiral-at-metal complexes after displacement of an asymmetric control ligand.^17^ Unlike the elegant chemistry of the Meggers’ group, our approach does not use a chiral auxiliary ligand as a removable resolving agent but rather as an integral part of a SAM complex that is necessarily diastereomeric. Such complexes are sought as precursors to heterobimetallic species that combine an inert, photoactive SAM center with a secondary metal ion(s) in a single molecule for (photo)­catalysis or molecular imaging. The strategy is to create new molecular architectures to explore the influence of the SAM unit on the behavior and/or activity of the second metals. The SAM moiety may be structurally benign and energetically active as a photon acceptor and redox relay. Our investigations toward these applications are at an early stage, and we report here some preliminary results related to the construction of single- diastereomers of an [Ir­(ppy)_2_(^*S,R*^ **L**)]­BF_4_ complex, where ^*S,R*^ **L** represents a bpy ligand with additional functionality capable of binding to other metal ions.

## Results and Discussion

### Synthesis, NMR Spectroscopy, and Computational Studies

The palladium-catalyzed C–N coupling between 6-bromo-2,2′-bipyridine and 1*R*,3*S*-diamino-1,2,2-trimethyl­cyclopentane is known to occur with a high degree of chemoselectivity to give *N* ^1^-([2,2′-bipyridin]-6-yl)-2,2,3-trimethyl­cyclopentane-1*S*,3*R*-diamine, ^*S,R*^ **L**, in 88% yield (Scheme 1).^31^ This tetramine ligand is composed of two connected parts: a bpy site and a diamine unit. The inclusion of the bpy moiety is to enable coordination to photoactive transition-metal centers, in this case, Ir­(III), while the chiral diamino ancillary is both an internal resolution module and a pivot for the construction of larger fragments to bind secondary metal ions. To achieve these goals, selective coordination of the bpy fragment to the [Ir­(ppy)_2_]^+^ core was necessary, and this was readily achieved through the 1:1 reaction of the ligand with [Ir­(ppy)_2_(MeCN)_2_]­BF_4_ at room temperature to give a 1:1 diastereomeric mixture of Λ- and Δ-[Ir­(ppy)_2_(^*S,R*^ **L**)]­BF_4_ (Λ,Δ-**Ir** ^***S,R***^ **1**) as a yellow solid in quantitative yield. Separation of the two diastereomers was achieved by fractional crystallization from acetone, with one isomer being poorly soluble and the other freely soluble (Scheme 1). In the ^1^H NMR spectrum of the least soluble isomer, a methyl group and one CH_2_ proton resonate significantly upfield of their positions in the spectrum of the free ligand whereas a CH_2_ hydrogen is seen upfield of TMS at −0.53 ppm for the other isomer. These unusual chemical shifts reflect orientations of the diamino group that position the highlighted hydrogens over a shielding region of an aromatic ring. This was confirmed upon determination of the solid-state structure of the least soluble form and acquisition of the ECD spectra (see below).

**Scheme 1:**
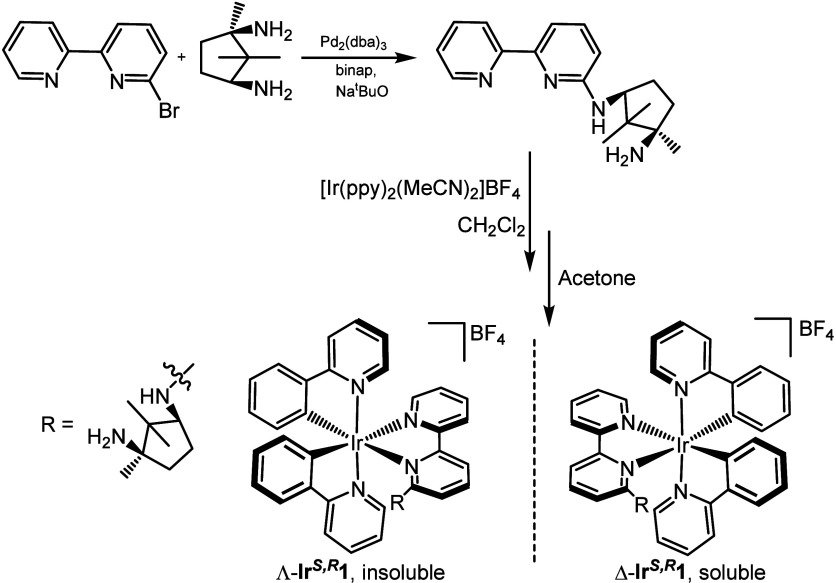
Synthetic Route to the Parent Diastereomers

DFT analysis of both SAM possibilities gave energy-minimized structures for the Λ and Δ configurations. For the Λ isomer, DFT predicts one methyl group and one H of a CH_2_ group to be proximate to aromatic rings of a ppy ligand. The chemical shift of the methyl protons of the noted group is predicted to be −0.40 ppm, while the H of CH_2_ is predicted to be a multiplet centered around 2.00 ppm. In the Δ isomer, only a single H of a CH_2_ group is found in close proximity to the ppy ligand, which is predicted to have a chemical shift of −0.55 ppm, and all methyl groups are situated far from any aromatic ring. DFT-predicted NMR spectra hence support the Λ assignment for the least soluble compound and Δ for the soluble form (Supporting Information, SI).

### Solid-State Structure

The molecular structure of Λ-**Ir** ^***S,R***^ **1** as determined by SCXRD is shown in Figure 1 along with pertinent metrics. In line with numerous other six-coordinate complexes of the type [Ir­(ppy)_2_(bpy)]^+^, the pyridine donors of the ppy ligands are mutually *trans* and the bpy nitrogens lie *trans* to the ppy carbon donors. The absolute configuration about the metal is Λ, as anticipated from the spectroscopic and DFT analysis. The location of the diamine substituent at position 6 of the bpy is expected to create some distortion in bond lengths and angles about the metal. This is evident in the longer Ir1–N4 bond of 2.205(6) Å compared to 2.135(8) Å for Ir1–N3 and deviations in the C12–Ir1–N4 and C11–Ir1–N4 bond angles away from the ideal with values of 168.4(3) and 106.1(3)°, respectively. Anilinic nitrogen N5 is planar with a C–N–C angle of 126.0(8)°, and the N*H* hydrogen (not shown) points toward one of the ppy ligands. This geometry enables the larger cyclopentanediamine group to be oriented as far away as possible from the coordinated ligands. Even so, elements of this group are positioned near one of the ppy ligands, notably a CH_3_ group and a CH_2_ hydrogen, as deduced above from the NMR data and DFT analysis. The hydrogens in question and their positions relative to the ppy ligand are shown in the molecular fragment on the right in Figure 1. To our surprise, a search of the CSD database (23/07/2025) for related complexes containing a 6-amino-bpy derivative yielded no results, so comparison between the geometric features seen here and known closely similar compounds is not possible. Even more surprising, a Scifinder search did not yield any derivatives of the type [Ir­(ppy)_2_(6-R-bpy)]^+^, where R is an amino group. For comparison, [Ir­(ppy)_2_(6-OMe-bpy)]^+^ shows similar but lesser bond length and angle distortions to Λ-**Ir** ^***S,R***^ **1**.^32^

**Figure 1:**
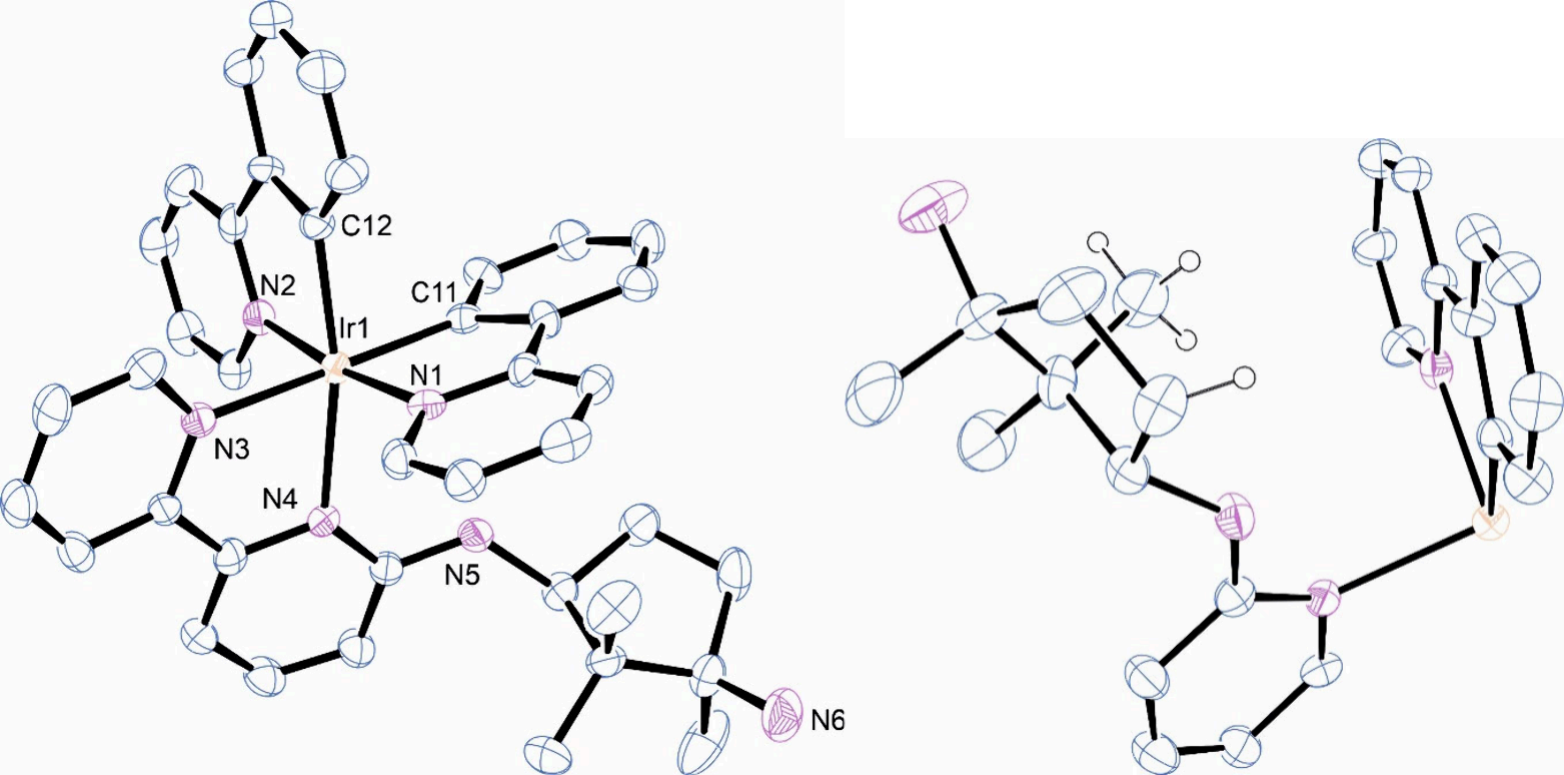
ORTEP view (left side) of the molecular structure of Λ-Ir^*S,R*^1. Hydrogen atoms and the BF_4_^–^ counterion have been omitted for clarity. Selected bond lengths (Å) and angles (deg): Ir1—N1 2.042(8); Ir1—N2 2.056(8); Ir1—N3 2.135(8); Ir1—N4 2.205(6); Ir1—C11 2.015(11); Ir1—C12 2.019(9); N1—Ir1—N2 172.9(3); C11—Ir1—N3 175.6(4); C12—Ir1—N4 168.4(3); C11—Ir1—N4 106.1(3); C11—Ir1—C12 83.6(3); N1—Ir1—N3 95.8(3); and N2—Ir1—N4 92.0(3). The right-hand partial structure shows the position of the methyl and CH_2_ hydrogen in relation to a ppy ligand (see the main text for context).

### Absorption and Circular Dichroism Spectroscopy

The UV–visible absorption spectra of the two isomeric complexes were obtained on equimolar MeCN solutions (2 × 10^–5^ M) and are very similar in appearance; the positions of the bands are comparable, and only a subtle difference in molar absorptivity was noted between the two species (Figure 2). In accordance with the benchmark [Ir­(ppy)_2_(bpy)]­PF_6_, the absorption features in the 200–300 nm range are attributed to spin-allowed, ligand-centered transitions^1^ (π–π*) that relate to the different aromatic constituents of the ppy and bpy ligands.^33^ The 300–450 nm region is known to comprise overlapping spin-allowed charge transfer (CT) bands. For these complexes, a clear feature is noted at ca. 380 nm with an additional shoulder at 405 nm within the band envelope; this absorption band is more clearly pronounced than for [Ir­(ppy)_2_(bpy)]­PF_6_. Thus, we expect metal-to-ligand, ligand-to-ligand, and intraligand charge transfer (MLCT, LLCT, and ILCT, respectively) to contribute to this region. These assignments were supported by DFT calculations: for both isomers, the lowest-energy absorption (ca. 430 nm for Λ/Δ) corresponds to HOMO–LUMO excitation, with further bands at 390–400 nm from HOMO – 1 and LUMO + 1 involvement. Differences between isomers predicted by DFT are very small and likely within the anticipated error associated with theoretical method used. HOMO and HOMO – 1 are largely metal d orbital-based while the LUMO is localized on the bipy ligand and LUMO + 1 is ppy-based (SI). As noted in similar Ir­(III) complexes, the weak shoulder that extends to ca. 500 nm is likely to include a lower-energy, spin-forbidden charge transfer feature (e.g., ^3^MLCT) that becomes weakly allowed due to spin orbit coupling effects.^33^

**Figure 2:**
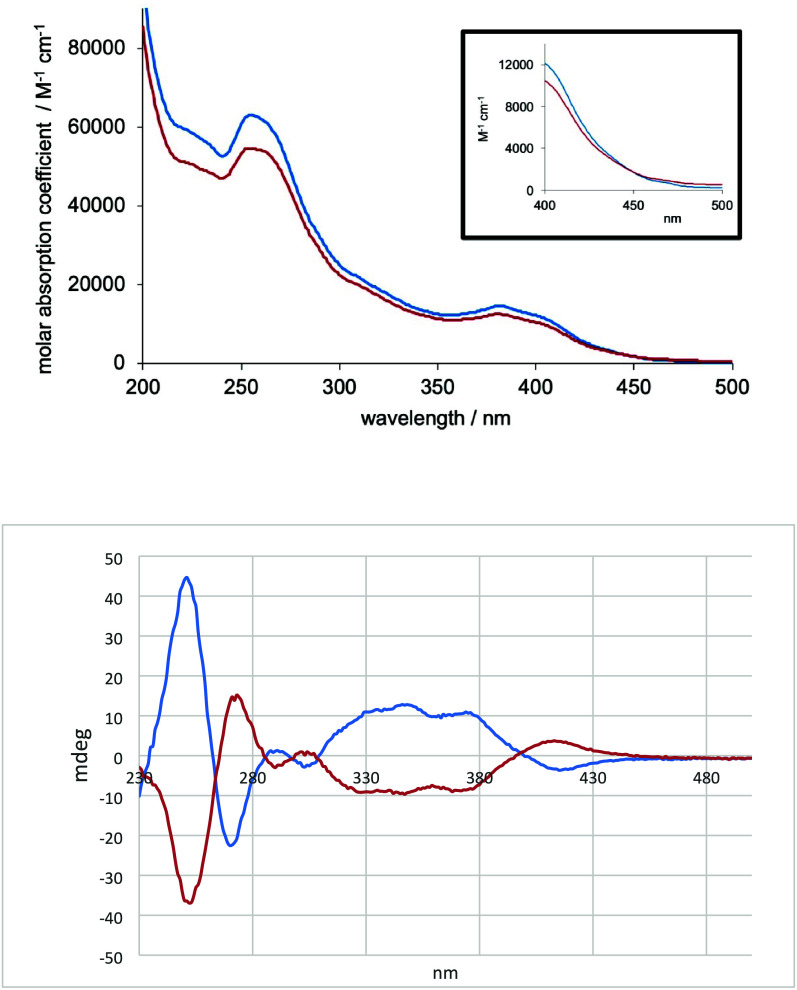
UV/vis absorption (top; with expansion shown in the inset) and CD spectra of Λ-Ir^*S,R*^1 (blue) and Δ-Ir^*S,R*^1 (red) recorded in MeCN (2 × 10^–5^ M) at 20 °C.

The ECD spectra of the two complexes are also shown in Figure 2. Although they are diastereomers and not enantiomers, the CD spectra are essentially mirror images and hence are dominated by the stereochemistry at the metal with little to no influence of the appended chiral diamine. This is unsurprising as the CD manifold is dictated by MLCT and ILCT transitions at the tris-chelate Ir­(III) center, as highlighted above. The CD profiles of both diastereomers show greater structure than is evident from the absorption spectra and closely mimic those of related systems.^34^

### Luminescence Studies

The photoluminescence properties were first assessed in aerated MeCN at room temperature. Using an excitation wavelength of 380 nm, the steady-state emission spectra reveal two features: one dominant signal at 435 nm and a second feature ca. 550 nm which is quite broad and featureless (Figure 3). The difference between the complexes was noted in the relative intensity of the 550 nm feature, which is more clearly defined in Λ-**Ir** ^***S,R***^ **1**. The positioning of this band was reiterated by using 415 nm excitation, which is expected to be more selective for the ^1^MLCT/^1^LLCT transitions (Figure 2). Time-resolved luminescence analysis of these features shows that the 435 nm band is likely to be from residual fluorescence (τ < 5 ns for Λ-**Ir** ^***S,R***^ **1** and Δ-**Ir** ^***S,R***^ **1**, respectively) probably associated with the amino-substituted bipy ligand. (Figure S74, Supporting Information). In comparison, the emission decay kinetics of the 550 nm features fit best using a biexponential where the resultant longer-lived component (τ = 79 and 65 ns for Λ-**Ir** ^***S,R***^ **1** and Δ-**Ir** ^***S,R***^ **1**, respectively) was dominant and indicative of a triplet emitting level, with subtle differences between the two isomers (Figure S75). When compared to [Ir­(ppy)_2_(bpy)]­PF_6_ (λ_em_ = 602 nm; τ = 275 ns), the blue shift in the emission wavelength for Λ-**Ir** ^***S,R***^ **1** and Δ-**Ir** ^***S,R***^ **1** is consistent with the substitution of the bipyridine ligand (which is the likely locale for the important LUMO) with an electron-donating amine group. Furthermore, low-temperature studies (77 K, EtOH glass doped with a MeCN solution of a metal complex (λ_ex_ = 415 nm) on the two species revealed the clear influence of rigidochromism and a vibronically structured emission peak with maximum intensities at 526 and 534 nm for Λ-**Ir** ^***S,R***^ **1** and Δ-**Ir** ^***S,R***^ **1**, respectively (for [Ir­(ppy)_2_(bpy)]­PF_6_, the peak is also hypsochromically shifted to 542 nm but is rather unstructured).^33^ The emission decays monoexponentially to give lifetime values of 3.58 μs (Λ-**Ir** ^***S,R***^ **1**) and 3.85 μs (Δ-**Ir** ^***S,R***^ **1**), which are slightly shorter than that for [Ir­(ppy)_2_(bipy)]­PF_6_ (4.77 μs) but nonetheless confirm the phosphorescent nature of the emission bands. Again, subtle differences in these photophysical parameters are noted between the isomers.

**Figure 3:**
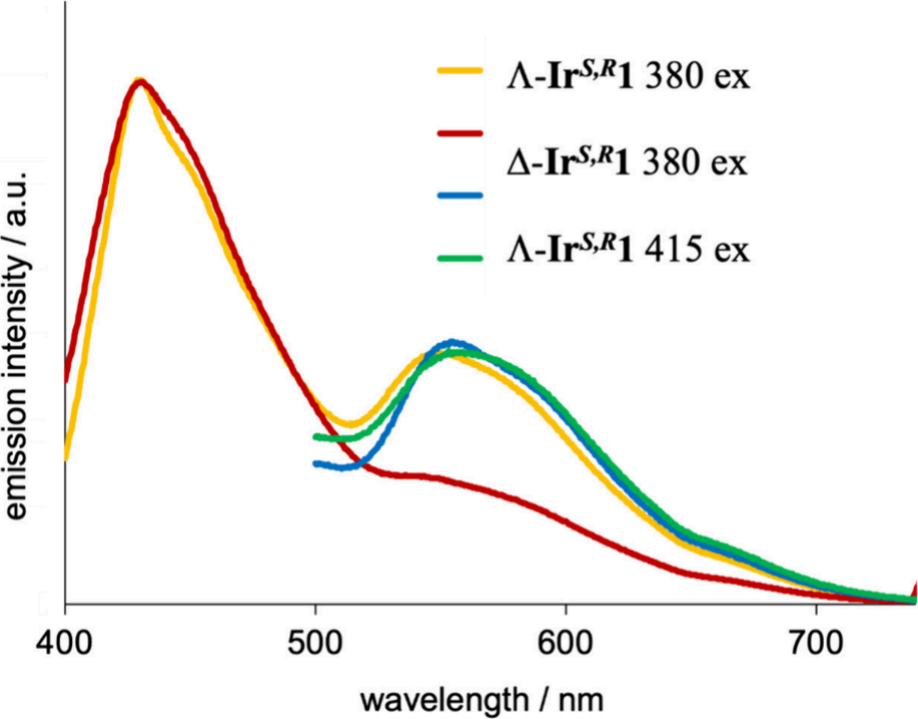
Emission spectra of the complexes at room temperature obtained using either 380 or 415 nm excitation (aerated MeCN).

**4:**
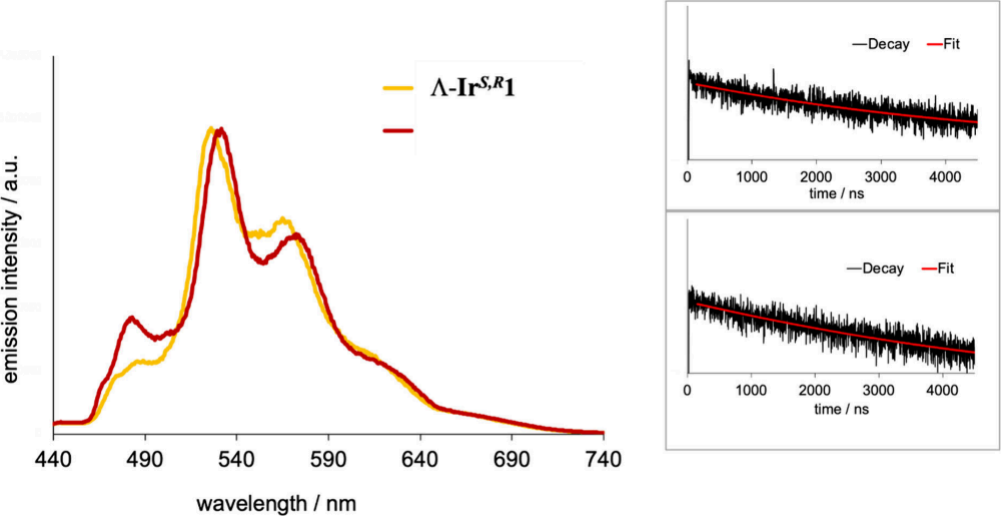
Emission spectra of the complexes at 77 K (EtOH/MeCN, λ_ex_ = 415 nm). (Inset) Lifetime decay traces (λ_ex_ = 295 nm) at 77 K for Λ-Ir^*S,R*^1 (top) and Δ-Ir^*S,R*^1 (bottom) fitted to give lifetimes of 3.58 μs (Λ-Ir^*S,R*^1) and 3.85 μs (Δ-Ir^*S,R*^1).

### Extended Structures and Bimetallic Complexes

The facility to add further functionality and ultimately introduce other metal ions is implicit in the ligand design. Although unreactive in the C–N coupling reaction to give the parent ligand, the residual primary amine group remains sufficiently nucleophilic to react with aldehydes and enable extension of the 6-substituent R. 2-Pyridinecarboxaldehyde was chosen as the starting substrate for functionalization, as it is known to react with the amino group in question and to provide a further donor for potential metal binding. Both diastereomers reacted cleanly with this aldehyde to give the imino-pyridine functionalized compounds Δ- and Λ-**Ir** ^***S,R***^ **2** (Figure 3). It is noted that the preparation of Δ-**Ir** ^***S,R***^ **2** required the use of molecular sieves to drive the equilibrium as the imine was sensitive to the presence of water. As anticipated, the integrity of the configuration at the Ir­(III) center is not compromised upon the addition of the extra external function as demonstrated by the ECD and NMR spectra. Little change is observed in the UV–vis and CD spectra with respect to the parent complexes, and the upfield signals noted above in the ^1^H NMR spectra of the relevant diastereomers of **Ir** ^***S,R***^ **1** remain in those for **Ir** ^***S,R***^ **2**, suggesting little change in the relative orientation of the cyclopentanediamine unit (SI). These spectral analogies extend to all of the other accessible derivatives shown in Figure 5. Δ-**Ir** ^***S,R***^ **4** and Δ-**Ir** ^***S,R***^ **5** are not included, as they could not be obtained in a pure state. This reflects a wider issue with the Δ-**Ir** ^***S,R***^ **1** parent as the condensation reactions between the primary amine and the small library of aldehydes reported here were never as clean as those for Λ-**Ir** ^***S,R***^ **1**, partly because of a greater susceptibility toward the reverse (hydrolysis) reaction. The Λ-**Ir** ^***S,R***^ **4** complex prepared from the reaction of Λ-**Ir** ^***S,R***^ **1** with 0.5 equiv of 2,6-pyridinedicarboxaldehyde has two chromophores per molecule and hence shows extinction coefficients approximately twice those for the other complexes in the UV–vis and CD spectra.

**Figure 5:**
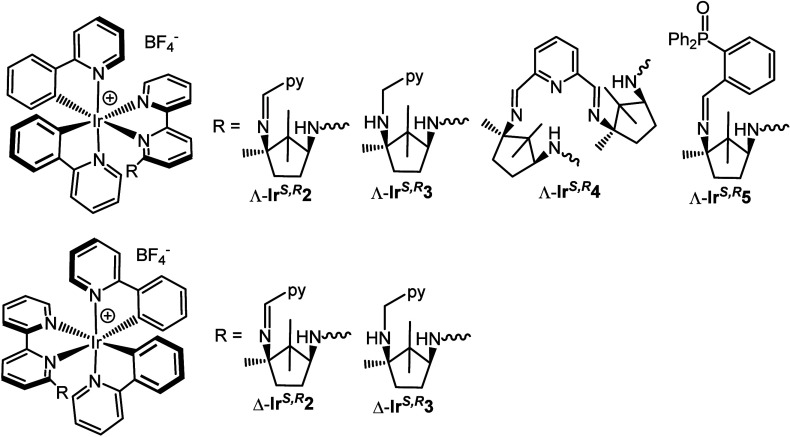
Functionalized derivatives.

In an initial screen as to the viability of the extended complexes as platforms for the construction of bimetallic complexes, in situ ^1^H NMR analysis of Λ-**Ir** ^***S,R***^ **2** and Δ-**Ir** ^***S,R***^ **2** in the presence of a slight excess of ZnI_2_ was performed. Λ-**Ir** ^***S,R***^ **1** was initially reacted with 2-pyridinecarboxaldehyde (1.05 equivs) in CD_3_CN for several hours, and the ^1^H NMR spectrum was recorded to ensure complete formation of the imine. ZnI_2_ (1.05 equiv) was subsequently added and the ^1^H NMR spectrum was obtained after a short period of time (30–120 min) and compared with the sample before addition of the zinc iodide. As is evident from Figure 6, there are significant changes in the spectrum after the addition of ZnI_2_ as exemplified by the downfield shift of the imine C*H* singlet from 8.16 to 8.57 ppm, suggesting complexation of the Zn­(II) ion by the imino-pyridine group (see the SI for the full spectrum). Similar chemical shift variations are noted in the in situ-prepared spectrum of Λ-**Ir** ^***S,R***^ **4** after addition of ZnI_2_, reflecting the binding of Zn­(II) at the diimino-pyridine unit, but in this case, there is evidence of a second, minor isomer. The addition of ZnI_2_ to preformed Δ-**Ir** ^***S,R***^ **2** appeared to compromise the C=N double bond as some aldehydic product was apparent in the ^1^H NMR spectrum of Δ-**Ir** ^***S,R***^ **2-Zn**. Some of the issues observed in the preparation of Δ-**Ir** ^***S,R***^ **2** and the attempted preparation of Δ-**Ir** ^***S,R***^ **4** and Δ-**Ir** ^***S,R***^ **5** are exacerbated in the presence of Zn­(II).

**Figure 6:**
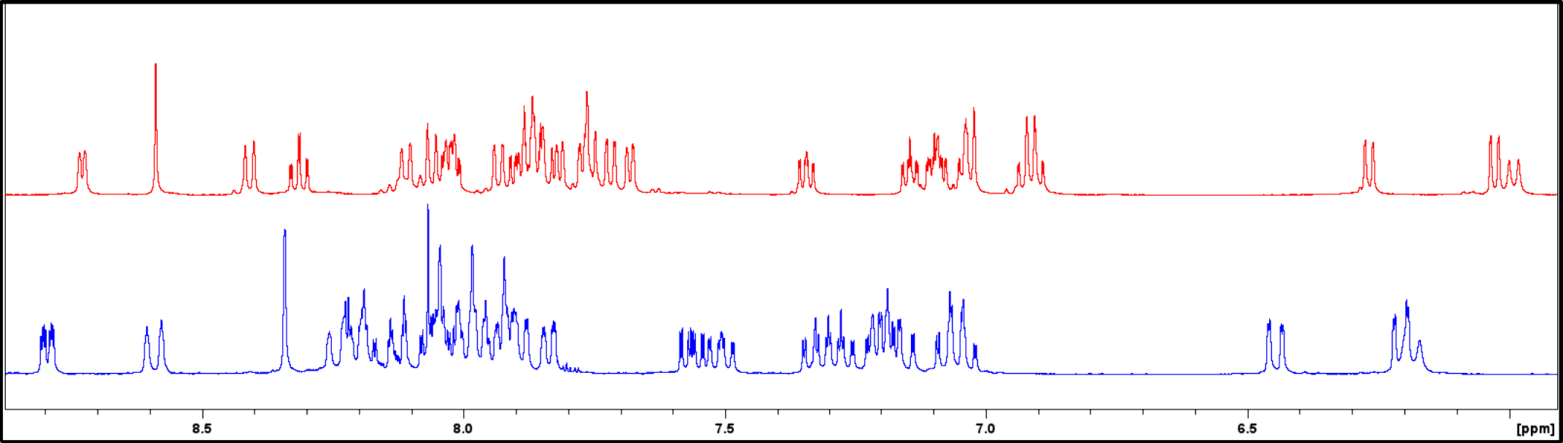
Comparison of the aromatic region of the ^1^H NMR spectra of Λ-Ir^*S,R*^2 (blue) and Λ-Ir^*S,R*^2-Zn (red) recorded in CD_3_CN.

The analysis of Λ-**Ir** ^***S,R***^ **3** and Δ-**Ir** ^***S,R***^ **3** was performed simply by adding a slight excess of ZnI_2_ to the preformed complexes in CD_3_CN. Although there is little change in the position of the aromatic resonances upon addition of the Zn­(II), appreciable differences are seen in the aliphatic region for Λ-**Ir** ^***S,R***^ **3**. The diastereomeric −C*H*_2_py hydrogens appear as a doublet of doublets and a virtual triplet separated by ∼0.7 ppm in the ^1^H NMR spectrum of Λ-**Ir** ^***S,R***^ **3-Zn**, and there is clear broadening in all of the signals of the trimethylcyclopentane unit. Although similar changes are observed for the benzylic CH_2_ hydrogens in the ^1^H NMR spectrum of Δ-**Ir** ^***S,R***^ **3-Zn**, there are numerous CH_3_ signals present, indicating a mixture of species in solution.

Due to an increased sensitivity of the imine bond in Λ-**Ir** ^***S,R***^ **5** toward hydrolysis, the samples for in situ NMR analysis were prepared and stored over molecular sieves at all stages prior to obtaining the spectra. As noted above, the most prominent change in the ^1^H NMR spectrum is associated with the imine C*H* hydrogen, although the coordination shift is now upfield upon complexation of Zn­(II) by Λ-**Ir** ^***S,R***^ **5**. This aside, there is little to distinguish between the ^1^H NMR spectra of Λ-**Ir** ^***S,R***^ **5** with and without the zinc. Far more compelling evidence for Zn­(II) binding is provided by the ^31^P­{^1^H} spectra where a shift from 28.6 ppm in Λ-**Ir** ^***S,R***^ **5** to 40.0 ppm in Λ-**Ir** ^***S,R***^ **5-Zn** is observed along with some broadening of the peak from ∼2.5 Hz at peak half height to 14 Hz; this is a very typical coordination shift for phosphine oxide ligands complexed to Zn­(II).^35,36^ Unfortunately, efforts to identify the parent molecular ions by MS were unsuccessful.

## Conclusions

*N* ^1^-([2,2′-Bipyridin]-6-yl)-2,2,3-trimethylcyclopentane-1*S*,3*R*-diamine, ^*S,R*^ **L**, has been used as a chiral auxiliary to enable easy separation of Δ- and Λ-[Ir­(ppy)_2_(^*S,R*^ **L**)]­BF_4_ diastereomers, both of which have been further functionalized at a peripheral amine group through condensation with selected aldehydes and, in some cases, subsequent reduction. The potential of these complexes as platforms for the formation of bimetallic complexes was explored through in situ ^1^H NMR studies with ZnI_2_. This proved successful with the Λ-[Ir­(ppy)_2_(^*S,R*^ **L**)]­BF_4_ derivatives but less so with the Δ forms, which showed the presence of more than one species in each case. We are continuing to explore the chemistry of these derivatives with other metal ions and to extend the library of substituents, and we will report on our findings in due course.

## Experimental Section

### General

All chemicals were purchased from commercial sources and used without further purification, unless otherwise stated. NMR spectra were recorded on Bruker Fourier 300, DPX 400, and Avance 500 or 600 MHz NMR spectrometers. ^1^H and ^13^C­{^1^H} NMR chemical shifts were referenced relative to the residual solvent resonances in the deuterated solvent. Mass spectra (ESI) were recorded on a Waters LCT Premier XE spectrometer. The UV/vis absorption and luminescence spectra of Λ- and Δ-**Ir** ^***S,R***^ **1** were obtained on MeCN solutions using a JobinYvon–Horiba Fluorolog spectrometer fitted with a JY TBX picosecond photodetection module. The pulsed source was a Nano-LED configured for 295 nm output operating at 1 MHz. Luminescence lifetime profiles were obtained using the JobinYvon–Horiba FluoroHub single photon counting module, and the data fits yielded the lifetime values using the provided DAS6 deconvolution software. All other UV/vis and all circular dichroism (CD) spectra were obtained on a Chirascan spectrometer (Applied Photophysics, Leatherhead, U.K.)

Single-crystal XRD data were collected on an Agilent SupaNova Dual Atlas diffractometer with a mirror monochromator using Mo (λ = 0.7107 Å) radiation and solved and refined using SHELXT^37^ and SHELXL.^38^ Non-hydrogen atoms were refined with anisotropic displacement parameters. Hydrogen atoms were inserted in idealized positions, and a riding model was used with Uiso set at 1.2 or 1.5 times the value of Ueq for the atom to which they are bonded. CCDC 2490619 contains the supplementary crystallographic data that can be obtained free of charge via https://www.ccdc.cam.ac.uk.

DFT calculations were performed using Orca v6.1.0.^39^ The initial structure of one isomer was built using solid state structure as a guide, and its isomeric form was constructed by inverting chirality at the metal but retaining it at the R group. Both were explored for conformational freedom using the GOAT^40^ tool of Orca with GFN2-xTB^41^ energies. The lowest energy so obtained was optimized first at the BP86/def2-SVP^42,43^ level, and analytical frequencies were calculated to confirm energy minima. Geometries were reoptimized at the r2SCAN-3c^44^ level, and spectroscopic properties were calculated using the single-point PBE0/def2-TZVPP^45^ method.

### *N*1-([2,2′-Bipyridin]-6-yl)-2,2,3-trimethylcyclopentane-1*S*,3*R*-diamine, ^*S*,*R*^ *L*

This was prepared by a modification of a reported procedure.^31^ A mixture of (1*R*,3*S*)-1,3-diamino-1,2,2-trimethylcyclopentane (1.21 g, 8.6 mmol), 6-bromobipyridine (2.00 g, 8.6 mmol), Pd_2_(dba)_3_ (0.12 g, 0.14 mmol), BINAP (348 mg, 0.56 mmol), and NaO^t^Bu (2.32 g, 23.3 mmol) in toluene (50 mL) was held at 100 °C for 48 h. After cooling, the mixture was extracted into 5 M aq. HCl (50 mL) through vigorous stirring over 30 min. The aqueous solution was isolated and extracted with CH_2_Cl_2_ (2 × 25 mL) before being made basic (use caution!) by the addition of solid NaOH with cooling and constant agitation. The basic solution was extracted into CH_2_Cl_2_ (3 × 50 mL), and the organic extracts were dried over MgSO_4_, filtered, and taken to dryness to yield a viscous oil which solidified on standing. Yield = 2.09 g (82%). ^1^H (CDCl_3_, 400 MHz): 8.56 (ddd, 7.4, 1.8, 0.9 Hz, 1H, H4), 8.25 (d, 8.0 Hz, 1H, H1), 7.69 (td, 7.8, 1.8 Hz, 1H, H2), 7.51 (dd, 7.4, 0.6 Hz, 1H, H5), 7.41 (t, 7.5 Hz, 1H, H6), 7.16 (ddd, 7.7, 6.1, 1.2 Hz, 1H, H3), 6.32 (d, 8.1 Hz, 1H, H7), 5.73 (d, 9.7 Hz, 1H, H8), 4.19 (td, 9.3, 3.4 Hz, 1H, H9), 2.21 (m, 1H, H10), 1.71 (m, 1H, H11), 1.54 (m, 2H, H10,11), 1.08 (s, 3H, H12), 0.90 (s, 3H, H13), 0.87 (s, 3H, H13) ppm. ^13^C­{^1^H} (CDCl_3_, 125 MHz): 158.5 (C), 157.1 (C), 154.2 (C), 149.0 (CH), 137.9 (CH), 136.6 (CH), 123.1 (CH), 109.3 (CH), 107.8 (CH), 61.4 (C), 60.5 (CH), 47.3 (C), 38.2 (CH_2_), 29.3 (CH_2_), 26.6 (CH_3_), 24.4 (CH_3_), 17.3 (CH_3_) ppm. HRMS (ES): *m*/*z* 297.2090 (calcd 297.2079) [M]^+^, 100%.

### Λ- and Δ-**Ir^*S*,*R*^1**

A solution of [Ir­(ppy)_2_(MeCN)_2_]­BF_4_ ^46^ (1.00 g, 1.49 mmol) and **L** (450 mg, 1.50 mmol) in CH_2_Cl_2_ (30 mL) was left to stand for 7 days at room temperature. The volatiles were removed in vacuo, and the solid residue was dissolved in acetone (30 mL). After the mixture was left to stand overnight, a crystalline yellow solid was isolated by filtration. Two additional crops were obtained upon further evaporation and cooling (4 °C) of the mother liquor. Combined yield of Λ-**Ir** ^***S,R***^ **1** (3 crops): 531 mg. The acetone soluble Δ-**Ir** ^***S,R***^ **1** was obtained as an orange solid after the complete removal of the acetone. Yield = 701 mg. ^1^H (Λ-**Ir-** ^***S,R***^ **1**, *d*_6_-DMSO, 400 MHz): 9.11 (d, 8.3 Hz, 1H), 8.74 (d, 8.2 Hz, 1H), 8.21 (d, 8.1 Hz, 1H), 8.63 (d, 8.1 Hz, 1H), 8.54 (t, 7.9 Hz, 1H), 8.49–8.27 (m, 3H), 8.23 (d, 7.5 Hz, 1H), 8.12 (m, 2H), 8.05 (d, 5.0 Hz, 1H), 7.92 (t, 6.8 Hz, 1H), 7.68 (m, 2H), 7.46 (d, 8.8 Hz, 1H), 7.40 (m, 2H), 7.27 (m, 2H), 6.53 (d, 7.4 Hz, 1H), 6.34 (d, 7.4 Hz, 1H), 6.21 (d, 8.7 Hz, 1H), 4.21 (q, 8.6 Hz, 1H), 3.77 (s br, 1H), 1.94 (m, 1H), 1.57 (m, 2H), 1.29 (s, 3H), 0.97 (s, 3H), 0.47 (m, 1H), 0.20 (s, 3H) ppm. ^13^C­{^1^H} (*d*_6_-DMSO, 125 MHz): 167.6 (C), 166.8 (C), 159.4 (C), 158.2 (C), 153.5 (C), 149.6 (CH), 149.4 (CH), 148.3 (C), 145.3 (C), 143.5 (C), 140.3 (CH), 139.7 (CH), 139.4 (CH), 139.0 (CH), 132.6 (CH), 131.2 (2 x CH), 129.8 (CH), 127.8 (CH), 126.6 (CH), 125.5 (CH), 125.2 (CH), 124.7 (CH), 124.3 (CH), 123.2 (CH), 122.8 (CH), 120.9 (CH), 120.8 (CH), 113.5 (CH), 111.0 (CH), 60.6 (C), 59.6 (CH), 48.1 (C), 37.3 (CH_2_), 26.1 (CH_3_), 25.8 (CH_2_), 22.6 (CH_3_), 17.6 (CH_3_) ppm. UV–Vis (MeCN): λ_max_/nm (ε/M^–1^ cm^–1^): 257 (62870), 384 (14410). CD (MeCN): λ_max_/nm (Δε/M^–1^ cm^–1^, g factor): 252 (+66.0, 1.0 × 10^–3^), 270 (−34.2, 5.9 × 10^–4^), 303 (−4.3, 1.7 × 10^–4^), 348 (+19.4, 1.46 × 10^–3^), 375 (+16.2, 1.05 × 10^–3^), 417 (−5.2, 6.9 × 10^–4^). HRMS (ES): *m*/*z* 797.2931 (calcd 797.2944) [M]^+^, 100%. ^1^H (Δ-**Ir** ^***S,R***^ **1**, *d*_6_-DMSO, 400 MHz): 8.73 (d, 8.4 Hz, 1H), 8.31 (t, 8.5 Hz, 2H), 8.19 (t, 5.5 Hz, 2H), 7.94 (m, 4H), 7.85 (t, 8.5 Hz, 1H), 7.59 (dd, 5.6, 1.1 Hz, 1H), 7.53 (m, 1H), 7.45 (dd, 5.9, 0.8 Hz, 1H), 7.34 (m, 1H), 7.19 (m, 1H), 6.99 (m, 2H), 6.86 (t, 7.6 Hz, 2H), 6.18 (m, 2H), 5.90 (dd, 7.7, 0.9 Hz, 1H), 3.73 (q, 8.6 Hz, 1H), 1.21 (m, 3H), 0.93 (s, 3H), 0.81 (s, 3H), 0.49 (s, 3H), −0.51 (m, 1H) ppm. ^13^C­{^1^H} (*d*_6_-DMSO, 125 MHz): 167.2 (C), 166.8 (C), 159.0 (C), 158.3 (C), 153.3 (C), 150.8 (CH), 149.6 (C), 149.4 (CH), 147.6 (C), 145.0 (C), 140.1 (CH), 139.8 (CH), 139.5 (CH), 139.3 (CH), 133.0 (CH), 131.1 (CH), 130.8 (CH), 130.1 (CH), 127.9 (CH), 126.3 (CH), 125.7 (CH), 125.4 (CH), 124.5 (CH), 124.3 (CH), 123.2 (CH), 123.0 (CH), 120.9 (CH), 120.6 (CH), 113.5 (CH), 110.5 (CH), 60.8 (C), 58.9 (CH), 47.7 (C), 36.9 (CH_2_), 25.8 (CH_3_), 25.1 (CH_2_), 22.1 (CH_3_), 19.0 (CH_3_) ppm. UV–Vis (MeCN): λ_max_/nm (ε/M^–1^ cm^–1^): 258 (54390), 384 (12360). CD (MeCN): λ_max_/nm (Δε/M^–1^ cm^–1^, g factor): 252 (−55.8, 9.4 × 10^–4^), 274 (+23.0, 4.7 × 10^–4^), 290 (−4.5, 1.4 × 10^–4^), 347 (−14.7, 1.2 × 10^–3^), 373 (−13.0, 9.4 × 10^–4^), 414 (+5.5, 6.6 × 10^–4^). HRMS (ES): *m*/*z* 797.2932 (calc. 797.2944) [M]^+^, 100%.

### Λ-**Ir^*S*,*R*^2**

A solution of **Λ-Ir** ^***S,R***^ **1** (55 mg, 6.2 × 10^–5^ mol) and 1.1 equiv of 2-pyridinecarboxaldehyde (7.3 mg, 6.85 × 10^–5^ mol) were stirred in MeCN (3 mL) at 50 °C for several hrs. Prior to heating, a yellow suspension was observed with little evidence of solubilization of the iridium complex. After the mixture was heated, a clear yellow solution resulted. The solution was left to cool, and all volatiles were removed in vacuo. The solid residue was triturated with Et_2_O (20 mL), filtered, and air-dried to give the desired compound as a yellow solid. Yield = 45 mg (75%). ^1^H (CD_3_CN, 500 MHz): 8.60 (ddd, 4.9, 1.7, 1.0 Hz, 1H), 8.40 (dt, 8.2, 0.9 Hz, 1H), 8.16 (s, 1H), 8.06 (ddd, 8.4, 1.3, 0.8 Hz, 1H), 8.00 (m, 2H), 7.94 (dt, 8.0, 1.1 Hz, 1H), 7.90–7.77 (m, 6H), 7.72–7.69 (m, 3H), 7.65 (ddd, 5.8, 1.5, 0.8 Hz, 1H), 7.37 (ddd, 7.5, 4.9, 1.3 Hz, 1H), 7.32 (ddd, 8.0, 5.6, 1.2 Hz, 1H), 7.14 (m, 1H), 7.09 (m, 1H), 7.03–6.96 (m, 4H), 6.90–6.82 (m, 2H), 6.26 (ddd, 7.6, 0.8, 0.4 Hz, 1H), 6.02 (ddd, 7.7, 1.2, 0.5 Hz, 1H), 6.00 (d, 9.0 Hz, 1H), 3.95 (q, 9.6 Hz, 1H), 1.82 (m, 1H), 1.62 (m, 1H), 1.52 (m, 1H), 1.10 (s, 3H), 0.82 (s, 3H), 0.43 (m, 1H), −0.18 (s, 3H) ppm. ^13^C­{^1^H} (CD_3_CN, 100 MHz): 168.1 (C), 167.4 (C), 160.1 (C), 158.8 (C), 158.2 (CH), 156.0 (C), 154.0 (C), 150.5 (C), 150.3 (C), 150.0 (CH), 149.8 (CH), 149.4 (CH), 148.5 (C), 145.8 (C), 144.0 (C), 140.0 (CH), 139.5 (CH), 139.2 (CH), 138.9 (CH), 137.2 (CH), 133.4 (CH), 131.5 (CH), 131.3 (CH), 130.5 (CH), 127.7 (CH), 126.5 (CH), 125.3 (CH), 124.9 (CH), 124.4 (CH), 124.0 (CH), 123.5 (CH), 123.4 (CH), 120.7 (CH), 120.6 (CH), 113.4 (CH), 111.0 (CH), 70.9 (C), 59.8 (CH), 49.0 (C), 32.6 (CH_2_), 26.5 (CH_2_), 24.3 (CH_3_), 22.2 (CH_3_), 16.2 (CH_3_) ppm. UV–Vis (MeCN): λ_max_/nm (ε/M^–1^ cm^–1^): 254 (65270), 377 (16580). CD (MeCN): λ_max_/nm (Δε/M^–1^ cm^–1^, g factor): 253 (+64.1, 9.7 × 10^–4^), 271 (−33.8, 6.0 × 10^–4^), 303 (−4.3, 1.7 × 10^–4^), 347 (+19.3, 1.45 × 10^–3^), 376 (+16.4, 1.05 × 10^–3^), 415 (−5.5, 6.6 × 10^–4^). HRMS (ES): *m*/*z* 886.3206 (calc. 886.3209) [M]^+^, 5%.

### Δ-**Ir^*S*,*R*^2**

This was prepared in a similar manner to that described for Λ-**Ir** ^***S,R***^ **2** except all reagents were soluble from the outset. The desired compound was obtained as an orange-yellow solid. Yield = 48 mg (80%). ^1^H (*d*_6_-acetone, 400 MHz): 8.69 (d, 8.3 Hz, 1H), 8.60 (ddd, 4.9, 1.6, 0.9 Hz, 1H), 8.44 (d, 5.8 Hz, 1H), 8.28 (d, 7.9 Hz, 1H), 8.25 (d, 8.0 Hz, 1H), 8.16 (s, 1H), 8.02–7.77 (m, 9H), 7.69 (dd, 5.9, 0.8 Hz, 1H), 7.48 (ddd, 7.8, 5.6, 1.0 Hz, 1H), 7.38 (ddd, 7.5, 4.9, 1.2 Hz, 1H), 7.30 (ddd, 7.5, 5.8, 1.3 Hz, 1H), 7.09 (d, 8.6 Hz, 1H), 7.00 (m, 2H), 6.84 (m, 2H), 6.39 (d, 9.1 Hz, 1H), 6.35 (dd, 7.6, 0.8 Hz, 1H), 6.06 (dd, 7.6, 0.9 Hz, 1H), 4.00 (q, 8.9 Hz, 1H), 1.70–1.47 (m, 3H), 1.14 (s, 3H), 1.11 (s, 3H), 0.59 (s, 3H), −0.20 (m, 1H) ppm. ^13^C­{^1^H} (*d*_6_-acetone, 100 MHz): 167.6 (C), 167.2 (C), 159.4 (C), 158.6 (C), 158.2 (CH), 155.6 (C), 153.5­(C), 150.9 (CH), 149.8 (CH), 149.4 (CH), 148.6 (CH), 147.3 (C), 145.0 (C), 139.8 (CH), 139.3 (CH), 138.9 (CH), 138.7 (CH), 136.4 (CH), 133.2 (CH), 130.7 (CH), 130.6 (CH), 130.2 (CH), 127.3 (CH), 126.0 (CH), 125.1 (CH), 124.8 (CH), 124.7 (CH), 123.8 (CH), 123.7 (CH), 122.9 (CH), 120.2 (CH), 120.1 (CH), 119.9 (CH), 113.2 (CH), 110.1 (CH), 70.2 (C), 58.6 (CH), 48.2 (C), 32.5 (CH_2_), 25.5 (CH_2_), 23.7 (CH_3_), 21.6 (CH_3_), 19.2 (CH_3_) ppm. UV–Vis (MeCN): λ_max_/nm (ε/M^–1^ cm^–1^): 253 (54450), 378 (13050). CD (MeCN): λ_max_/nm (Δε/M^–1^ cm^–1^, g factor): 252 (−42.3, 7.8 × 10^–4^), 271 (+12.4, 2.7 × 10^–4^), 304 (+5.9, 3.0 × 10^–4^), 328 (−10.6, 8.0 × 10^–4^), 345 (−10.9, 1.04 × 10^–3^), 372 (−9.4, 8.0 × 10^–4^), 411 (+5.2, 7.5 × 10^–4^). HRMS (ES): *m*/*z* 886.3206 (calc. 886.3209) [M]^+^, 5%.

### Λ-**Ir^*S*,*R*^3**

A solution of Λ-**Ir** ^***S,R***^ **1** (55 mg, 6.2 × 10^–5^ mol) and 2 equiv of 2-pyridinecarboxaldehyde (13.3 mg, 1.24 × 10^–4^ mol) were stirred in MeOH (3 mL) at 50 °C for several hours. After cooling, the volatiles were removed and the residue was dissolved in MeOH to which an excess of NaBH_4_ was added portionwise, and the whole was left to stir overnight. On return, the clear orange solution was taken to dryness, triturated with water, filtered, and air-dried. Yield = 47 mg (78%). ^1^H (*d*_6_-DMSO, 400 MHz): 8.71 (d, 8.2 Hz, 1H), 8.49 (d, 4.4 Hz, 1H), 8.35 (d, 8.2 Hz, 1H), 8.24 (d, 8.0 Hz, 1H), 8.15 (t, 7.8 Hz, 1H), 8.08–7.88 (m, 6H), 7.84 (d, 7.7 Hz, 1H), 7.74 (m, 2H), 7.65 (d, 5.1 Hz, 1H), 7.53 (t, 6.6 Hz, 1H), 7.39 (d, 7.9 Hz, 1H), 7.27 (m, 2H), 7.12 (d, 8.8 Hz, 1H), 7.00 (m, 2H), 6.90 (t, 7.3 Hz, 1H), 6.85 (t, 7.4 Hz, 1H), 6.14 (d, 7.5 Hz, 1H), 5.94 (d, 7.6 Hz, 1H), 5.84 (d, 8.8 Hz, 1H), 3.90 (q, 9.1 Hz, 1H), 3.76 (dd, 14.2, 6.7 Hz, 1H), 3.63 (dd, 14.2, 7.4 Hz, 1H), 1.72 (t, 6.9 Hz, 1H), 1.56 (m, 1H), 1.35 (m, 1H), 1.21 (m, 1H), 1.04 (s, 3H), 0.67 (s, 3H), 0.16 (m, 1H), −0.10 (s, 3H) ppm. ^13^C­{^1^H} (*d*_6_-DMSO, 125 MHz): 167.5 (C), 166.8 (C), 161.5 (C), 159.5 (C), 158.2 (C), 153.5 (C), 149.6 (CH), 149.5 (C), 149.4 (CH), 149.0 (CH), 148.2 (C), 145.3 (C), 143.5 (C), 140.3 (CH), 139.8 (CH), 139.4 (CH), 139.1 (CH), 136.8 (CH), 132.7 (CH), 131.2 (CH), 129.8 (CH), 127.9 (CH), 126.6 (CH), 125.5 (CH), 125.2 (CH), 124.7 (CH), 124.4 (CH), 123.3 (CH), 122.9 (CH), 122.4 (CH), 122.2 (CH), 120.9 (CH), 120.8 (CH), 113.6 (CH), 111.0 (CH), 63.4 (C), 59.6 (CH), 49.1 (C), 48.7 (CH_2_), 34.7 (CH_2_), 25.8 (CH_2_), 23.0 (CH_3_), 22.4 (CH_3_), 17.6 (CH_3_) ppm. UV–Vis (MeCN): λ_max_/nm (ε/M^–1^ cm^–1^): 251 (62560), 382 (14450). CD (MeCN): λ_max_/nm (Δε/M^–1^ cm^–1^, g factor): 251 (+53.5, 8.6 × 10^–4^), 271 (−27.7, 5.1 × 10^–4^), 304 (−3.6, 1.7 × 10^–4^), 350 (+16.2, 1.33 × 10^–3^), 375 (+15.2, 1.05 × 10^–3^), 419 (−4.4, 6.2 × 10^–4^). HRMS (ES): *m*/*z* 888.3343 (calcd 888.3366) [M]^+^, 100%.

### Δ-**Ir^*S*,*R*^3**

This was prepared in a manner similar to that described for Λ-**Ir** ^***S,R***^ **3** and obtained as an orange-yellow solid. Yield = 42 mg (70%). ^1^H (CD_3_CN, 400 MHz): 8.50 (d, 4.5 Hz, 1H), 8.46 (d, 8.4 Hz, 1H), 8.29 (d, 5.5 Hz, 1H), 8.16–8.04 (m, 3H), 7.96–7.67 (m, 10H), 7.52 (d, 5.8 Hz, 1H), 7.37 (m, 2H), 7.20 (m, 2H), 7.07 (m, 3H), 6.91 (m, 2H), 6.32 (d, 7.4 Hz, 1H), 6.27 (d, 9.0 Hz, 1H), 6.05 (d, 7.8 Hz, 1H), 3.88 (dd, 13.9, 6.7 Hz, 1H), 3.74 (m, 2H), 1.66 (t, 6.8 Hz, 1H), 1.37 (m, 3H), 1.12 (s, 3H), 1.01 (s, 3H), 0.68 (s, 3H), −0.31 (m, 1H) ppm. ^13^C­{^1^H} (CD_3_CN, 100 MHz): 169.0 (C), 168.5 (C), 162.8 (C), 161.0 (C), 159.9 (C), 154.8 (C), 151.9­(CH), 151.0 (CH), 149.6 (CH), 146.3 (C), 144.8 (C), 140.6 (CH), 140.1 (CH), 139.7 (CH), 139.5 (CH), 137.2 (CH), 134.5 (CH), 131.8 (CH), 131.7 (CH), 131.6 (CH), 128.0 (CH), 126.9 (CH), 126.1 (CH), 125.6 (CH), 124.6 (CH), 124.3 (CH), 124.1 (CH), 124.0 (CH), 123.0 (CH), 122.6 (CH), 121.0 (CH), 114.2 (CH), 111.1 (CH), 64.8 (C), 61.7 (CH), 49.8 (CH_2_), 49.7 (C), 35.6 (CH_2_), 26.7 (CH_2_), 23.8 (CH_3_), 22.3 (CH_3_), 19.4 (CH_3_) ppm. UV–Vis (MeCN): λ_max_/nm (ε/M^–1^ cm^–1^): 251 (61970), 379 (13990). CD (MeCN): λ_max_/nm (Δε/M^–1^ cm^–1^, g factor): 252 (−47.1, 7.8 × 10^–4^), 273 (+13.5, 2.7 × 10^–4^), 302 (+3.3, 1.4 × 10^–4^), 328 (−12.1, 7.8 × 10^–4^), 347 (−12.3, 1.07 × 10^–3^), 371 (−10.8, 4.4 × 10^–4^), 414 (+5.0, 6.1 × 10^–4^). HRMS (ES): *m*/*z* 888.3408 (calcd 888.3366) [M]^+^, 100%.

### Λ-**Ir^*S*,*R*^4**

A solution of Λ-**Ir** ^***S,R***^ **1** (50 mg, 5.6 × 10^–5^ mol) and 0.5 equiv of 2,6-pyridinedicarboxaldehyde (3.8 mg, 2.8 × 10^–5^ mol) were stirred in MeCN (3 mL) at 50 °C overnight. Prior to heating, a yellow suspension was observed with little evidence of solubilization of the iridium complex. After heating, a clear yellow solution resulted. The solution was left to cool, and all volatiles were removed in vacuo. The solid residue was triturated with Et_2_O (20 mL), filtered, and air-dried to give the desired compound as a yellow solid. Yield = 38 mg (70%). ^1^H (CD_3_CN, 400 MHz): 8.40 (d, 8.3 Hz, 2H), 8.17 (s, 2H), 8.08–7.94 (m, 8H), 7.90–7.62 (m, 18H), 7.32 (ddd, 7.6, 5.6, 1.0 Hz, 2H), 7.14 (m, 2H), 7.09 (ddd, 7.6, 5.9, 1.4 Hz, 2H), 7.00 (m, 3H), 6.87 (m, 2H), 6.26 (dd, 7.7, 0.7 Hz, 2H), 6.01 (dd, 7.8, 1.1 Hz, 2H), 6.00 (d, 8.7 Hz, 2H), 3.95 (q, 9.1 Hz, 2H), 1.84 (m, 2H), 1.58 (m, 4H), 1.11 (s, 6H), 0.84 (s, 6H), 0.44 (m, 2H), −0.18 (s, 6H) ppm. ^13^C­{^1^H} (CD_3_CN, 100 MHz): 168.1 (C), 167.5 (C), 160.2 (C), 158.8 (C), 158.3 (C), 157.8 (CH), 154.1 (C), 150.5 (C), 150.3 (C), 149.8 (CH), 149.5 (CH), 148.5 (C), 145.8 (C), 144.0 (C), 140.1 (CH), 139.5 (CH), 139.2 (CH), 138.9 (CH), 133.4 (CH), 131.5 (CH), 131.3 (CH), 130.5 (CH), 127.7 (CH), 126.5 (CH), 125.4 (CH), 124.9 (CH), 124.4 (CH), 124.0 (CH), 123.6 (CH), 123.2 (CH), 120.7 (CH), 120.6 (CH), 113.5 (CH), 111.0 (CH), 71.1 (C), 59.9 (CH), 49.1 (C), 32.7 (CH_2_), 26.6 (CH_2_), 24.3 (CH_3_), 22.2 (CH_3_), 18.2 (CH_3_) ppm. UV–Vis (MeCN): λ_max_/nm (ε/M^–1^ cm^–1^): 251 (114430), 378 (26105). CD (MeCN): λ_max_/nm (Δε/M^–1^ cm^–1^, g factor): 251 (+134.9, 1.18 × 10^–3^), 270 (−64.7, 6.4 × 10^–4^), 293 (+5.0, 1.0 × 10^–4^), 348 (+34.1, 1.55 × 10^–3^), 376 (+29.9, 1.16 × 10^–3^), 417 (−7.3, 6.0 × 10^–4^). HRMS (ES): (parent not observed) *m*/*z* 914.3134 amu [C_47_H_43_N_7_OIr]^+^, 50%.

### Λ-**Ir^*S*,*R*^5**

A solution of Λ-**Ir** ^***S,R***^ **1** (50 mg, 5.6 × 10^–5^ mol) and 1 equiv of 2-(diphenylphosphoryl)­benzaldehyde (17.1 mg, 5.6 × 10^–5^ mol) were stirred in MeOH (8 mL) at 50 °C overnight. After heating, a clear yellow solution resulted. The solution was left to cool, and all volatiles were removed in vacuo. The solid residue was triturated with Et_2_O (20 mL), filtered, and air-dried to give the desired compound as a yellow solid. Yield = 64 mg (96%). ^1^H (*d*_6_-DMSO, 400 MHz): 8.66 (d, 8.3 Hz, 1H), 8.56 (s, 1H), 8.26 (d, 8.2 Hz, 1H), 8.19 (d, 8.0 Hz, 1H), 8.09 (t, 7.8 Hz, 1H), 8.06–7.84 (m, 6H), 7.78 (d, 7.8 Hz, 1H), 7.72–7.43 (m, 16H), 7.24 (t, 6.6 Hz, 1H), 7.19 (t, 6.1 Hz, 1H), 7.05 (m, 2H), 6.94 (m, 2H), 6.85 (t, 7.4 Hz, 1H), 6.80 (t, 7.4 Hz, 1H), 6.08 (d, 7.6 Hz, 1H), 5.88 (d, 7.6 Hz, 1H), 5.72 (d, 8.5 Hz, 1H), 3.86 (q, 8.8 Hz, 1H), 1.57 (m, 1H), 1.05 (m, 1H), 0.91 (m, 1H), 0.56 (s, 6H), −0.04 (m, 1H), −0.41 (s, 3H) ppm. ^13^C­{^1^H} (*d*_6_-DMSO, 100 MHz): 162.7 (d, 2.0 Hz, C), 154.2 (C), 153.5 (C), 151.4 (d, 6.6 Hz, CH), 148.5 (C), 145.4 (CH), 144.9 (CH), 144.2 (C), 143.3 (CH), 139.6 (C), 137.9 (C), 136.2 (d, 7.0 Hz, C), 135.4 (CH), 134.8 (CH), 133.6 (CH), 133.4 (CH), 129.0 (d, 11.0 Hz, CH), 128.4 (d, 11.6 Hz, CH), 128.3 (CH), 128.0 (d, 12.0 Hz, CH), 127.9 (C), 127.8 (d, 3.3 Hz, C), 127.2 (2 x CH), 127.6 (d, 2.5 Hz, CH), 127.3 (CH), 127.2 (CH), 127.1 (CH), 127.0 (d, 9.6 Hz, CH), 126.4 (CH), 126.2 (CH), 125.5 (CH), 124.5 (CH), 124.2 (CH), 124.1 (CH), 124.0 (d, 8.9 Hz, CH), 123.9 (d, 9.2 Hz, CH), 122.8 (d, 9.0 Hz, CH), 122.3 (CH), 120.9 (CH), 120.5 (CH), 120.2 (CH), 118.5 (d, 34.8 Hz, CH), 118.3 (CH), 115.1 (d, 37.9 Hz, CH), 108.9 (CH), 104.7 (CH), 72.5 (C), 54.2 (CH), 43.4 (C), 27.8 (CH_2_), 20.8 (CH_2_), 19.1 (CH_3_), 17.7 (CH_3_), 14.6 (CH_3_) ppm. ^31^P­{^1^H} (*d*_6_-DMSO 161 MHz): 28.1 ppm. UV–Vis (MeCN): λ_max_/nm (ε/M^–1^ cm^–1^): 255 (65480), 382 (13640). CD (MeCN): λ_max_/nm (Δε/M^–1^ cm^–1^, g factor): 251 (+50.3, 7.7 × 10^–4^), 271 (−27.4, 5.3 × 10^–4^), 304 (−3.2, 1.5 × 10^–4^), 347 (+14.7, 1.36 × 10^–3^), 377 (+12.4, 8.7 × 10^–4^), 417 (−3.8, 5.7 × 10^–4^). LRMS (ES): *m*/*z* 1085.36 [M]^+^, 40%; 543.19 [M]^2+^, 100%. HRMS could not be obtained due to interference from a +1 amu species.

### In Situ ^1^H NMR Experiments

#### Λ-**Ir^*S*,*R*^2-Zn**

A solution of Λ-**Ir** ^***S,R***^ **1** (20 mg, 2.25 × 10^–5^ mol) and 1.05 equiv of 2-pyridinecarboxaldehyde (2.88 mg, 2.5 × 10^–5^ mol) were stirred in CD_3_CN (2 mL) at 50 °C for 2 h. After cooling, the ^1^H NMR spectrum was recorded to ensure completion of the reaction, whereupon ZnI_2_ (8 mg, 2.5 × 10^–5^ mol) was added, the mixture was stirred for 2 h, and the ^1^H NMR spectrum was recorded (see the SI for stacked spectra). ^1^H (CD_3_CN, 500 MHz): 8.70 (d, 4.8 Hz, 1H), 8.57 (s, 1H), 8.43 (m, 1H), 8.29 (td, 7.8, 1.5 Hz, 1H), 8.08 (d, 8.2 Hz, 1H), 8.05 (d, 7.8 Hz, 1H), 8.00 (m, 2H), 7.93–7.71 (m, 7H), 7.69 (dd, 7.8, 0.9 Hz, 1H), 7.66 (d, 5.9 Hz, 1H), 7.31 (ddd, 7.0, 6.1, 1.0 Hz, 1H), 7.13 (ddd, 7.3, 6.0, 1.2 Hz, 1H), 7.10–6.97 (m, 4H), 6.88 (m, 2H), 6.24 (d, 7.4 Hz, 1H), 6.00 (d, 7.5 Hz, 1H), 5.96 (d, 9.0 Hz, 1H), 3.95 (q, 9.0 Hz, 1H), 2.27 (m, 1H), 1.84 (m, 1H), 1.46 (s, 3H), 0.95 (s, 3H), 0.43 (m, 1H), −0.04 (s, 3H) ppm.

#### Λ-**Ir^*S*,*R*^3-Zn**

Λ-**Ir** ^***S,R***^ **3** (20 mg, 2.05 × 10^–5^ mol) was dissolved in CD_3_CN (1 mL) with gentle warming, and the ^1^H NMR spectrum of the cooled solution was recorded prior to the addition of ZnI_2_ (7 mg, 2.2 × 10^–5^ mol). After approximately 30 min, the ^1^H NMR spectrum was obtained (see the SI for stacked spectra). ^1^H (CD_3_CN, 500 MHz): 8.49 (d, 5.1 Hz, 1H), 8.41 (d, 8.2 Hz, 1H), 8.10 (d, 8.0 Hz, 1H), 8.08–7.96 (m, 5H), 7.91 (td, 6.7, 1.5 Hz, 1H), 7.87–7.79 (m, 4H), 7.75–7.69 (m, 3H), 7.62 (ddd, 5.8, 1.3, 0.8 Hz, 1H), 7.59 (m, 2H), 7.53 (d, 8.0 Hz, 1H), 7.32 (ddd, 7.7, 5.7, 1.1 Hz, 1H), 7.12 (m, 2H), 6.99 (m, 3H), 6.87 (m, 2H), 6.25 (dd, 7.7, 0.9 Hz, 1H), 6.02 (d, 8.8 Hz, 1H), 6.00 (dd, 7.7, 0.9 Hz, 1H), 4.22 (dd, 15.3, 9.0 Hz, 1H), 3.87 (q, 8.9 Hz, 1H), 3.51 (t, 7.7 Hz, 1H), 1.76 (s vbr, 3H), 1.00 (s vbr, 3H), 0.51 (m br, 1H), 0.12 (s br, 3H) ppm.

#### Λ-**Ir^*S*,*R*^4-Zn**

The experiment was performed as detailed for Λ-**Ir** ^***S,R***^ **2-Zn** except using 0.5 equiv of 2,6-pyridinedicarboxaldehyde. ^1^H (CD_3_CN, 500 MHz): 8.76 (s, 2H), 8.40–8.29 (m, 3H), 8.20 (d, 7.8, 2H), 8.06 (d, 8.0 Hz, 2H), 8.00 (m, 3H), 7.88–7.77 (m, 9H), 7.76–7.64 (m, 8H), 7.31 (m, 2H), 7.15 (ddd, 7.9, 5.9, 1.2 Hz, 2H), 7.08 (ddd, 7.6, 5.9, 1.3 Hz, 2H), 7.05–6.97 (m, 5H), 6.90–8.82 (m, 3H), 6.23 (dd, 7.6, 0.9 Hz, 2H), 6.00 (dd, 7.8, 0.9 Hz, 2H), 5.95 (d, 8.8 Hz, 2H), 3.95 (q, 9.0 Hz, 2H), 2.14 (m, 2H), 1.88–1.70 (m, 4H), 1.32 (s, 6H), 0.91 (s, 6H), 0.40 (m, 2H), −0.13 (s, 6H) ppm.

#### Λ-**Ir^*S*,*R*^5-Zn**

The experiment was performed as detailed for Λ-**Ir** ^***S,R***^ **2-Zn** except using 1 equiv of 2-(diphenylphosphoryl)­benzaldehyde. Molecular sieves were present at all times during the preparation of the sample prior to filtering before obtaining the NMR spectra. ^1^H (CD_3_CN, 400 MHz): 8.43 (s, 1H), 8.38 (d, 8.2 Hz, 1H), 8.17 (dd, 7.7, 4.2 Hz, 1H), 8.05 (d, 8.1 Hz, 1H), 8.00 (m, 2H), 8.05 (d, 7.8 Hz, 1H), 8.00 (m, 2H), 7.93–7.41 (m, 17H), 7.30 (t, 6.4 Hz, 1H), 7.21 (dd, 15.0, 7.7 Hz, 1H), 7.10 (m, 2H), 7.00 (t, 7.2 Hz, 2H), 6.94 (d, 9.1 Hz, 1H), 6.90–6.80 (m, 2H), 6.20 (d, 7.4 Hz, 1H), 6.00 (d, 7.5 Hz, 1H), 5.85 (d, 8.7 Hz, 1H), 3.78 (q, 9.0 Hz, 1H), 1.62 (m, 1H), 1.30–1.04 (m, 2H), 0.67 (s, 3H), 0.63 (s, 3H), 0.16 (m, 1H), −0.32 (s, 3H) ppm. ^31^P­{^1^H} (CD_3_CN, 162 MHz): 40.0 ppm.

## Supplementary Material



## Notes

NMR, MS, computational data, and tables of relevant crystallographic data and figures for the compounds (PDF)
