# Supplementary material for: Stereogenic-at-Metal Ir(III) Complexes as Platforms for the Construction of Asymmetric Bimetallic Complexes
Source: Inorg Chem. 2025 Dec 9;64(50):24744–53. doi: 10.1021/acs.inorgchem.5c04469 (PMC12728929; doi:10.1021/acs.inorgchem.5c04469)
Supplement: Supplementary file 1 [file ic5c04469_si_001.pdf]

Supplementary material.

Supporting Information for

Stereogenic-at-Metal Ir(III) complexes as Platforms for the Construction of Asymmetric Bimetallic Complexes

Paul D. Newman,* James A. Platts, Simon J. A. Pope and Benson M. Kariuki

School of Chemistry, Cardiff University, Park Place, Cardiff, Wales, UK CF10 3AT. *Corresponding author: Paul D. Newman, E-mail: newmanp1@cardiff.ac.uk, ORCID: 0000-0002-1808-1211.

Contents

Table S1. SCXRD collection and refinement data for **Λ-Ir^{S,R}1**.

Figure S1. Molecular structure of **Λ-Ir^{S,R}1**.

Figure S2. Geometry optimised structure for one of two possible conformers and calculated ^{1}H NMR chemical shifts for **Λ-Ir^{S,R}1**.

Figure S3. Geometry optimised structure and calculated ^{1}H NMR chemical shifts for **Δ-Ir^{S,R}1**.

Table S2. Calculated absorption spectra.

Table S3. Optimised Cartesian Coordinates.

Figure S4. Electronic spectra of the complexes. Λ-**Ir^{S,R}2** (brown); Δ-**Ir^{S,R}2** (grey); Λ-**Ir^{S,R}3** (yellow); Δ-**Ir^{S,R}3** (light blue); Λ-**Ir^{S,R}4** (green); Λ-**Ir^{S,R}5** (dark blue)

Figure S5. CD spectra of the complexes. Λ-**Ir^{S,R}2** (green); Δ-**Ir^{S,R}2** (dark blue); Λ-**Ir^{S,R}3** (orange); Δ-**Ir^{S,R}3** (grey); Λ-**Ir^{S,R}4** (yellow); Λ-**Ir^{S,R}5** (light blue)

Figure S6. ^{1}H NMR (400 MHz, $CDCl_3$) of *N*^{1}-([2,2'-bipyridin]-6-yl)-2,2,3-trimethylcyclopentane-1*S*,3*R*-diamine, ^{S,R}**L**.

Figure S7. ^{13}C{^{1}H} NMR (125 MHz, $CDCl_3$) of *N*^{1}-([2,2'-bipyridin]-6-yl)-2,2,3-trimethylcyclopentane-1*S*,3*R*-diamine, ^{S,R}**L**.

Figure S8. ^{1}H,^{13}C HSQC NMR (400 MHz, $CDCl_3$) of *N*^{1}-([2,2'-bipyridin]-6-yl)-2,2,3-trimethylcyclopentane-1*S*,3*R*-diamine, ^{S,R}**L**.

Figure S9. ^{1}H,^{1}H COSY NMR (400 MHz, $CDCl_3$) of *N*^{1}-([2,2'-bipyridin]-6-yl)-2,2,3-trimethylcyclopentane-1*S*,3*R*-diamine, ^{S,R}**L**.

Figure S10. ^{1}H,^{1}H NOESY NMR (400 MHz, $CDCl_3$) of *N*^{1}-([2,2'-bipyridin]-6-yl)-2,2,3-trimethylcyclopentane-1*S*,3*R*-diamine, ^{S,R}**L**.

Figure S11. HRMS of *N*^{1}-([2,2'-bipyridin]-6-yl)-2,2,3-trimethylcyclopentane-1*S*,3*R*-diamine, ^{S,R}**L**.

Figure S12. ^{1}H NMR (400 MHz, DMSO-d_6) of Λ-**Ir^{S,R}1**.

Figure S13. ^{13}C{^{1}H} NMR (125 MHz, DMSO-d_6) of Λ-**Ir^{S,R}1**.

Figure S14. ^{13}C DEPT NMR (125 MHz, DMSO-d_6) of Λ-**Ir^{S,R}1**.

Figure S15. ^{1}H,^{13}C HSQC NMR (400 MHz, DMSO-d_6) of Λ-**Ir**^{S,R}**1**.

Figure S16. ^{1}H,^{1}H COSY NMR (400 MHz, DMSO-d_6) of Λ-**Ir**^{S,R}**1**.

Figure S17. ^{1}H,^{1}H NOESY NMR (400 MHz, DMSO-d_6) of Λ-**Ir**^{S,R}**1**.

Figure S18. HRMS of Λ-**Ir**^{S,R}**1**.

Figure S19. ^{1}H NMR (400 MHz, DMSO-d_6) of Δ-**Ir**^{S,R}**1**.

Figure S20. ^{13}C{^{1}H} NMR (125 MHz, DMSO-d_6) of Δ-**Ir**^{S,R}**1**.

Figure S21. ^{13}C DEPT NMR (125 MHz, DMSO-d_6) of Δ-**Ir**^{S,R}**1**.

Figure S22. ^{1}H,^{13}C HSQC NMR (400 MHz, DMSO-d_6) of Δ-**Ir**^{S,R}**1**.

Figure S23. ^{1}H,^{1}H COSY NMR (400 MHz, DMSO-d_6) of Δ-**Ir**^{S,R}**1**.

Figure S24. ^{1}H,^{1}H NOESY NMR (400 MHz, DMSO-d_6) of Δ-**Ir**^{S,R}**1**.

Figure S25. HRMS of Δ-**Ir**^{S,R}**1**.

Figure S26. ^{1}H NMR (500 MHz, MeCN-d_3) of Λ-**Ir**^{S,R}**2**.

Figure S27. ^{13}C{^{1}H} NMR (100 MHz, MeCN-d_3) of Λ-**Ir**^{S,R}**2**.

Figure S28. ^{13}C DEPT NMR (100 MHz, MeCN-d_3) of Λ-**Ir**^{S,R}**2**.

Figure S29. ^{1}H,^{13}C HSQC NMR (500 MHz, MeCN-d_3) of Λ-**Ir**^{S,R}**2**.

Figure S30. ^{1}H,^{1}H COSY NMR (500 MHz, MeCN-d_3) of Λ-**Ir**^{S,R}**2**.

Figure S31. ^{1}H,^{1}H NOESY NMR (500 MHz, MeCN-d_3) of Λ-**Ir**^{S,R}**2**.

Figure S32. HRMS of Λ-**Ir**^{S,R}**2**.

Figure S33. ^{1}H NMR (400 MHz, acetone-d_6) of Δ-**Ir**^{S,R}**2**.

Figure S34. ^{13}C{^{1}H} NMR (100 MHz, acetone-d_6) of Δ-**Ir**^{S,R}**2**.

Figure S35. ^{13}C DEPT NMR (100 MHz, acetone-d_6) of Δ-**Ir**^{S,R}**2**.

Figure S36. ^{1}H,^{13}C HSQC NMR (100 MHz, acetone-d_6) of Δ-**Ir**^{S,R}**2**.

Figure S37. ^{1}H,^{1}H COSY NMR (400 MHz, acetone-d_6) of Δ-**Ir**^{S,R}**2**.

Figure S38. ^{1}H,^{1}H NOESY NMR (400 MHz, acetone-d_6) of Δ-**Ir**^{S,R}**2**.

Figure S39. HRMS of Δ-**Ir**^{S,R}**2**.

Figure S40. ^{1}H NMR (400 MHz, DMSO-d_6) of Λ-**Ir**^{S,R}**3**.

Figure S41. ^{13}C{^{1}H} NMR (125 MHz, DMSO-d_6) of Λ-**Ir**^{S,R}**3**.

Figure S42. ^{13}C DEPT NMR (125 MHz, DMSO-d_6) of Λ-**Ir**^{S,R}**3**.

Figure S43. ^{1}H,^{13}C HSQC NMR (400 MHz, DMSO-d_6) of Λ-**Ir**^{S,R}**3**.

Figure S44. ^{1}H,^{1}H COSY NMR (400 MHz, DMSO-d_6) of Λ-**Ir**^{S,R}**3**.

Figure S45. ^{1}H,^{1}H NOESY NMR (400 MHz, DMSO-d_6) of Λ-**Ir**^{S,R}**3**.

Figure S46. HRMS of Λ-**Ir**^{S,R}**3**.

Figure S47. ^{1}H NMR (400 MHz, MeCN-d_3) of Δ-**Ir^{S,R}3**.

Figure S48. ^{13}C{^{1}H} NMR (100 MHz, MeCN-d_3) of Δ-**Ir^{S,R}3**.

Figure S49. ^{13}C DEPT NMR (100 MHz, MeCN-d_3) of Δ-**Ir^{S,R}3**.

Figure S50. ^{1}H,^{13}C HSQC NMR (400 MHz, MeCN-d_3) of Δ-**Ir^{S,R}3**.

Figure S51. ^{1}H,^{1}H COSY NMR (400 MHz, MeCN-d_3) of Δ-**Ir^{S,R}3**.

Figure S52. HRMS of Δ-**Ir^{S,R}3**.

Figure S53. ^{1}H NMR (400 MHz, MeCN-d_3) of Λ-**Ir^{S,R}4**.

Figure S54. ^{13}C{^{1}H} NMR (100 MHz, MeCN-d_3) of Λ-**Ir^{S,R}4**.

Figure S55. ^{13}C DEPT NMR (100 MHz, MeCN-d_3) of Λ-**Ir^{S,R}4**.

Figure S56. ^{1}H,^{13}C HSQC NMR (400 MHz, MeCN-d_3) of Λ-**Ir^{S,R}4**.

Figure S57. ^{1}H,^{1}H COSY NMR (400 MHz, MeCN-d_3) of Λ-**Ir^{S,R}4**.

Figure S58. ^{1}H,^{1}H NOESY NMR (400 MHz, MeCN-d_3) of Λ-**Ir^{S,R}4**.

Figure S59. HRMS of Λ-**Ir^{S,R}4**.

Figure S60. ^{1}H NMR (400 MHz, MeCN-d_3) of Λ-**Ir^{S,R}5**.

Figure S61. ^{1}H NMR (400 MHz, DMSO-d_6) of Λ-**Ir^{S,R}5**.

Figure S62. ^{13}C{^{1}H} NMR (100 MHz, DMSO-d_6) of Λ-**Ir^{S,R}5**.

Figure S63. ^{13}C DEPT NMR (100 MHz, DMSO-d_6) of Λ-**Ir^{S,R}5**.

Figure S64. ^{31}P{^{1}H} NMR (161 MHz, DMSO-d_6) of Λ-**Ir^{S,R}5**.

Figure S65. LRMS of Λ-**Ir^{S,R}5**.

Figure S66. ^{1}H NMR (500 MHz, MeCN-d_3) of Λ-**Ir^{S,R}2-Zn**.

Figure S67. ^{1}H NMR (500 MHz, MeCN-d_3) of Λ-**Ir^{S,R}2** (bottom) and Λ-**Ir^{S,R}2-Zn** (top).

Figure S68. ^{1}H NMR (500 MHz, MeCN-d_3) of Δ-**Ir^{S,R}2** (bottom) and Δ-**Ir^{S,R}2-Zn** (top).

Figure S69. ^{1}H NMR (500 MHz, MeCN-d_3) of Λ-**Ir^{S,R}3** (bottom) and Λ-**Ir^{S,R}3-Zn** (top).

Figure S70. ^{1}H NMR (500 MHz, MeCN-d_3) of Λ-**Ir^{S,R}4** (bottom) and Λ-**Ir^{S,R}4-Zn** (top).

Figure S71. Expanded aromatic region of the ^{1}H NMR (500 MHz, MeCN-d_3) of Λ-**Ir^{S,R}4** (bottom) and Λ-**Ir^{S,R}4-Zn** (top).

Figure S72. ^{1}H NMR (400 MHz, MeCN-d_3) of Λ-**Ir^{S,R}5** (bottom) and Λ-**Ir^{S,R}5-Zn** (top).

Figure S73. ^{31}P{^{1}H} NMR (162 MHz, DMSO-d_6) of Λ-**Ir^{S,R}5-Zn**.

Figure S74. Fitted emission decay traces for Λ-**Ir^{S,R}1** (**left**, fitted parameters τ = 2.2 ns, χ^2 = 1.2) and Δ-**Ir^{S,R}1 (right,** fitted parameters τ = 2.2 ns, χ^2 = 0.97) for the emission feature noted ca. 435 nm.

Figure S75. Fitted emission decay traces for Λ-**Ir^{S,R}1** (**left**, fitted parameters τ = 4.1 ns, and 65 ns (84%), χ^2 = 1.1) and Δ-**Ir^{S,R}1** (**right,** fitted parameters τ = 5.2 ns and 79 ns (83%), χ^2 = 1.1) for the emission feature noted ca. 550 nm.

Compound (Identification code)	**Λ-Ir^{S,R}1**
CCDC reference	2490619
Empirical formula	$C_{40}H_{40}BF_4N_6Ir$
Formula weight	883.79
Temperature /K	293(2)
Wavelength /Å	0.71073
Crystal system	Orthorhombic
Space group	P 21 21 21
a/Å	13.9084(7)
b/Å	15.3147(6)
c/Å	16.3328(7)
α/°	90
β/°	90
γ/°	90
Volume/$Å^3$	3478.9(3)
Z	4
Density (calculated)/ Mgm^{-3}	1.687
Absorption coefficient/ mm^{-1}	3.899
S1Crystal size/ mm^3	0.250x0.020x0.020
Reflections collected	8185
Independent reflections	6186
R(int)	0.0478
Data / restraints / parameters	8185 / 236 / 522
Goodness-of-fit on F^2	1.020
R1, wR2 [I>2σ(I)]	0.0437, 0.0764
R1, wR2 (all data)	0.0693, 0.0810
Largest diff. peak and hole e.$Å^{-3}$	1.411 and -0.749
Abs. structure (Flack) parameter	-0.033(7)

Table S1. SCXRD collection and refinement data for **Λ-Ir^{S,R}1**.

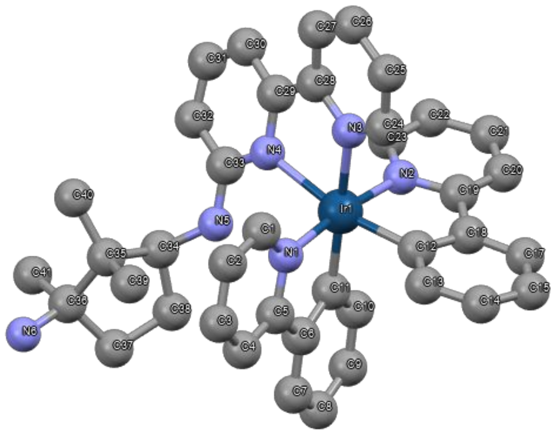

Figure S1. Molecular structure of **Λ-Ir**^{S,R}**1**. Hydrogen atoms and BF_4^- counterion have been omitted for clarity. Selected bond lengths (Å) and angles (°): Ir1—N1 2.042(8); Ir1—N2 2.056(8); Ir1—N3 2.135(8); Ir1—N4 2.205(6); Ir1—C11 2.015(11); Ir1—C12 2.019(9); N1—Ir1—N2 172.9(3); C11—Ir1—N3 175.6(4); C12—Ir1—N4 168.4(3); C11—Ir1—N4 106.1(3); C11—Ir1—C12 83.6(3); N1—Ir1—N3 95.8(3); N2—Ir1—N4 92.0(3).

Calculations.

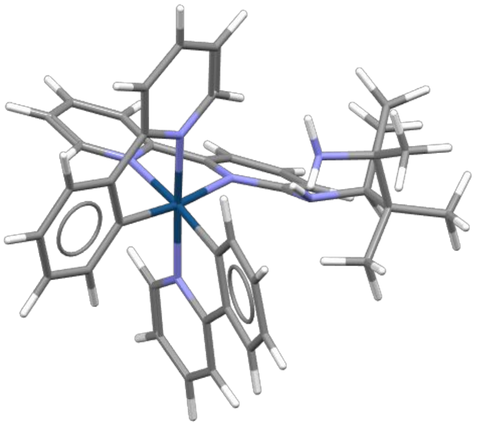

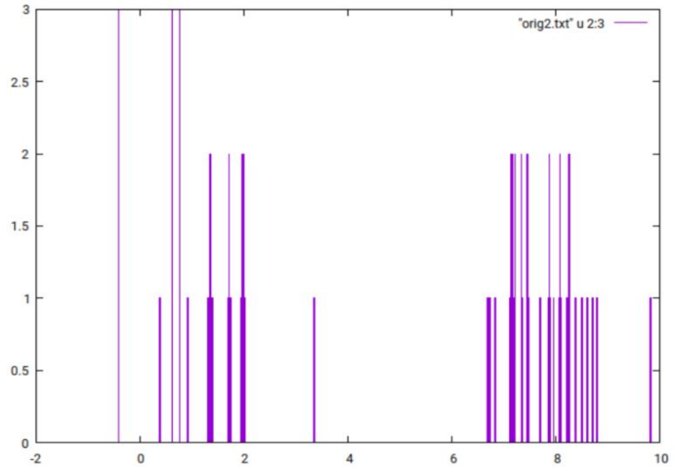

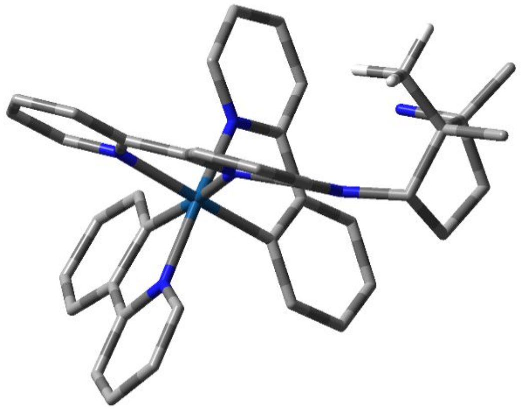

CH_3 (shown) at -0.4 ppm.

Figure S2. Geometry optimised structure for one of two possible conformers and calculated ^{1}H NMR chemical shifts for **Λ-Ir^{S,R}1**. The lower figure highlights the diagnostic hydrogens and the relative position of the diamine fragment.

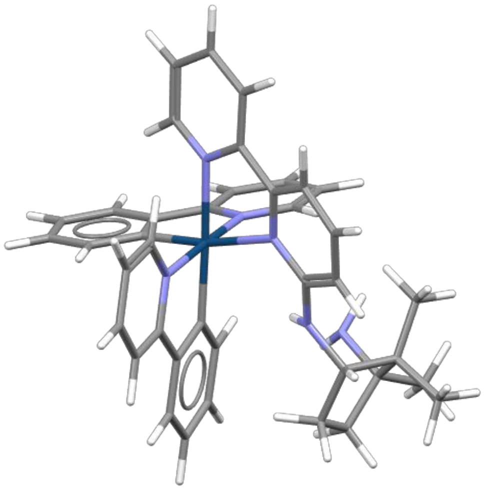

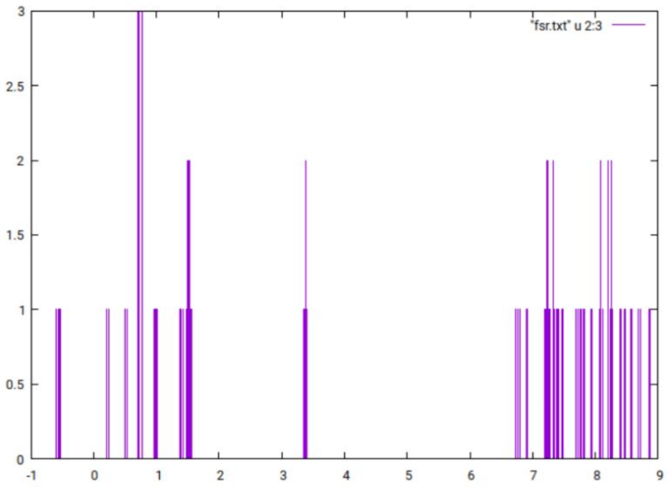

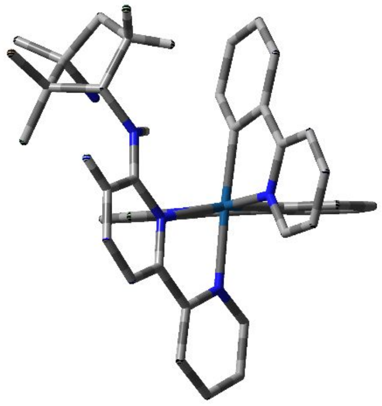

H of CH_2 next to chiral carbon (shown) -0.57 ppm

Figure S3. Geometry optimised structure and calculated ^{1}H NMR chemical shifts for **Δ-Ir^{S,R}1**. The lower figure highlights the diagnostic hydrogens and the relative position of the diamine fragment.

	λ /nm	f	
Λ	432	0.02	HOMO → LUMO
	400	0.13	HOMO-1 → LUMO
	393	0.08	HOMO → LUMO+1
Δ	429	0.02	HOMO → LUMO
	401	0.13	HOMO-1 → LUMO
	389	0.06	HOMO → LUMO+1

Table S2. Calculated absorption spectra.

Λ orbitals

HOMO	HOMO-1

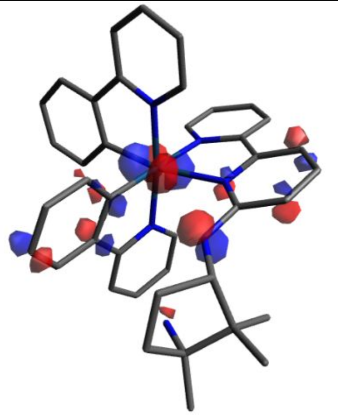	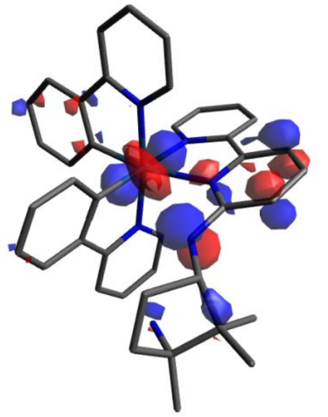
LUMO	LUMO+1
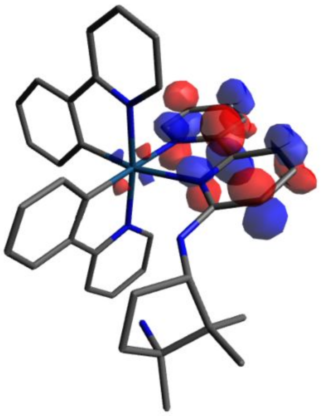	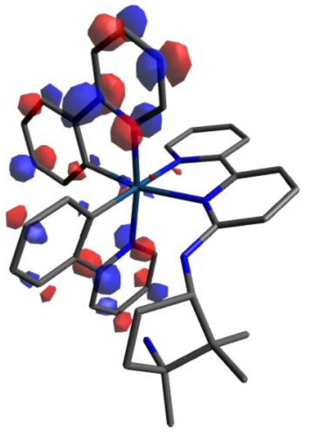

Δ orbitals

HOMO	HOMO-1
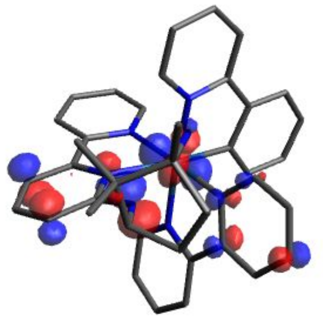	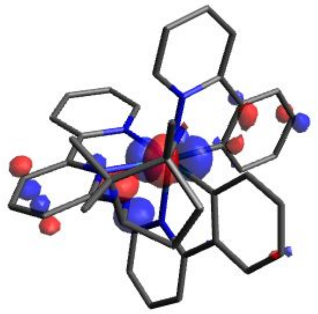
LUMO	LUMO+1

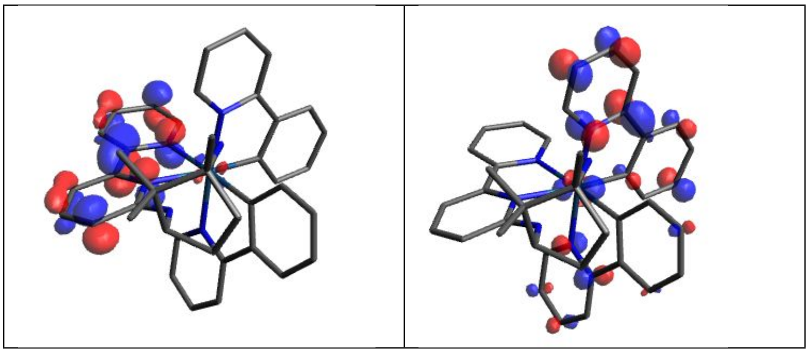

Λ-**Ir**^{S,R}**1**

Ir	-0.230565	0.757043	0.657940
N	-0.483905	2.489060	-0.440325
N	-0.379890	-0.342057	-1.197929
N	1.869396	0.556098	-0.036192
C	-1.786758	2.918825	-0.564211
C	0.520164	3.200770	-1.005138
C	0.286641	4.366507	-1.737994
C	-1.036906	4.813539	-1.893181
C	-2.073272	4.085381	-1.300084
C	-2.767552	2.080620	0.130567
C	-3.117878	0.128776	1.541699
C	-4.502920	0.373754	1.521253
C	-5.027480	1.470012	0.808264
C	-4.159433	2.323018	0.113336
H	1.536274	2.816627	-0.851272
H	1.136200	4.908260	-2.175968
H	-1.258082	5.725491	-2.467167
H	-3.115442	4.418132	-1.398265
H	-2.739619	-0.738825	2.102817
H	-5.182516	-0.299223	2.068251
H	-6.111771	1.657156	0.794628
H	-4.569729	3.178221	-0.445091
C	0.752894	-0.409926	-1.952475
C	0.734644	-1.033466	-3.216221
C	-0.457116	-1.587881	-3.695578
C	-1.613862	-1.512432	-2.901592
C	-1.532134	-0.877946	-1.659035
C	1.970499	0.175774	-1.350178
C	3.144835	0.344963	-2.092023
C	4.255984	0.935074	-1.458440
C	4.189045	1.265414	-0.113212
C	2.983136	1.021705	0.617605
H	1.651507	-1.096443	-3.816103
H	-0.480402	-2.080282	-4.678746
H	-2.570574	-1.938479	-3.234556
H	-2.405399	-0.783000	-0.997219
H	3.192238	0.051643	-3.147186
H	5.182168	1.117216	-2.023218
H	5.060652	1.689036	0.399573
C	-0.285502	0.844667	3.567388
C	-0.341757	1.362186	4.880400
C	-0.272131	2.745504	5.089963
C	-0.148865	3.612035	3.983341
C	-0.096637	3.100520	2.675545
C	-0.292791	-0.584891	3.252344
C	-0.405338	-1.641279	4.177179
C	-0.319465	-2.967259	3.742447
C	-0.120659	-3.228003	2.374179
C	-0.050530	-2.150228	1.489383
H	-0.432310	0.685191	5.743974
H	-0.311011	3.151088	6.112078
H	-0.091642	4.700306	4.145981
H	-0.000488	3.798867	1.829717
H	-0.550424	-1.411210	5.241649
H	-0.401252	-3.793428	4.464088
H	-0.032547	-4.252564	1.987482
H	0.080281	-2.296370	0.408701
C	-2.222449	0.969933	0.846975
N	2.880890	1.258695	1.944265
C	3.988843	1.596081	2.830693

C	3.455892	2.368920	4.071792
C	4.742252	0.343164	3.418317
H	4.697614	2.224794	2.260377
C	3.608642	1.423631	5.284540
H	2.400607	2.653674	3.901886
H	4.019417	3.310918	4.215729
C	3.876396	0.022699	4.692496
H	4.460460	1.725385	5.925427
H	2.708664	1.413382	5.933351
C	4.831528	-0.844008	2.450676
H	5.294793	-1.716696	2.955638
H	3.839714	-1.157230	2.071959
H	5.463465	-0.599062	1.573902
C	6.169821	0.785876	3.793362
H	6.178961	1.631868	4.509005
H	6.745735	-0.046709	4.243692
H	6.711495	1.112886	2.882121
H	2.094142	0.814573	2.441260
C	4.531899	-0.932654	5.698959
H	3.843538	-1.101921	6.552901
H	4.740336	-1.919261	5.234538
H	5.483136	-0.534034	6.105049
N	2.571538	-0.492276	4.218584
H	2.664580	-1.465613	3.897258
H	1.924670	-0.532195	5.016926
N	-0.152797	-0.866636	1.909263
C	-0.156893	1.710133	2.435187

Δ-Ir^{S,R}1

Ir	0.319917	-0.345264	-0.798506
N	0.528905	-2.300040	-1.374424
N	0.798146	-0.970907	1.211542
N	-1.653269	-0.504156	0.228509
C	1.735876	-2.632358	-1.949599
C	-0.434625	-3.236329	-1.215419
C	-0.266622	-4.552600	-1.650475
C	0.945529	-4.909842	-2.268445
C	1.950301	-3.946319	-2.409910
C	2.692387	-1.523637	-2.003179
C	3.085882	0.821668	-1.472674
C	4.393761	0.705550	-1.976652
C	4.856385	-0.520319	-2.497778
C	4.006407	-1.634669	-2.510524
H	-1.354772	-2.895942	-0.721753
H	-1.077848	-5.279207	-1.504953
H	1.108087	-5.935857	-2.630430
H	2.909933	-4.205276	-2.877520
H	2.742722	1.790563	-1.077644
H	5.061657	1.581901	-1.967028
H	5.880293	-0.604265	-2.892308
H	4.369393	-2.592491	-2.914170
C	-0.225916	-0.992691	2.112016
C	0.001795	-1.381602	3.447012
C	1.289784	-1.755254	3.846834
C	2.330503	-1.737231	2.902933
C	2.041236	-1.339449	1.594635
C	-1.554899	-0.604631	1.590263
C	-2.637004	-0.356647	2.442081
C	-3.867225	0.016497	1.864238
C	-3.996072	0.071304	0.484643
C	-2.870946	-0.236506	-0.344237
H	-0.828676	-1.407923	4.164293
H	1.477490	-2.064888	4.885388

H	3.356189	-2.027660	3.170178
H	2.816515	-1.300717	0.815410
H	-2.526115	-0.427598	3.530446
H	-4.730154	0.249315	2.505670
H	-4.958014	0.327586	0.024547
C	-0.338436	1.773468	-2.703629
C	-0.602311	2.423237	-3.930724
C	-0.614991	1.686375	-5.122654
C	-0.351823	0.301680	-5.086195
C	-0.101770	-0.347512	-3.865701
C	-0.233190	2.470346	-1.422021
C	-0.455287	3.845244	-1.205840
C	-0.288818	4.391883	0.070279
C	0.108028	3.552131	1.126630
C	0.311569	2.195923	0.864534
H	-0.785427	3.508431	-3.958536
H	-0.818855	2.188831	-6.080176
H	-0.340417	-0.277242	-6.023516
H	0.101041	-1.428163	-3.867257
H	-0.760962	4.478699	-2.049522
H	-0.463848	5.464360	0.241620
H	0.259968	3.935123	2.145156
H	0.629140	1.503525	1.655296
C	2.211183	-0.284719	-1.472555
N	-2.943410	-0.270037	-1.692389
C	-4.166999	-0.211565	-2.483345
C	-3.865381	0.378978	-3.888428
C	-4.794653	-1.623937	-2.772689
H	-4.894772	0.421536	-1.942295
C	-3.881472	-0.807196	-4.881781
H	-4.626545	1.137139	-4.155723
H	-2.889161	0.897744	-3.874155
C	-3.959274	-2.091899	-4.022788
H	-2.985672	-0.826148	-5.535233
H	-4.763290	-0.756346	-5.550500
C	-6.280279	-1.413611	-3.126856
H	-6.420016	-0.736821	-3.992919
H	-6.810603	-0.965636	-2.261492
H	-6.777568	-2.375216	-3.362356
C	-4.706587	-2.593679	-1.587740
H	-5.126752	-3.582733	-1.863774
H	-5.289235	-2.213428	-0.725064
H	-3.666018	-2.748566	-1.245201
H	-2.177732	-0.758773	-2.188286
N	0.140718	1.663887	-0.368951
C	-0.110962	0.362408	-2.645362
N	-2.583426	-2.391261	-3.556741
H	-2.566804	-3.298216	-3.070638
H	-1.972530	-2.511673	-4.375583
C	-4.562394	-3.278951	-4.787239
H	-3.909022	-3.544338	-5.644419
H	-4.640742	-4.173407	-4.134015
H	-5.571121	-3.054621	-5.187648

Table S3. Optimised Cartesian Coordinates.

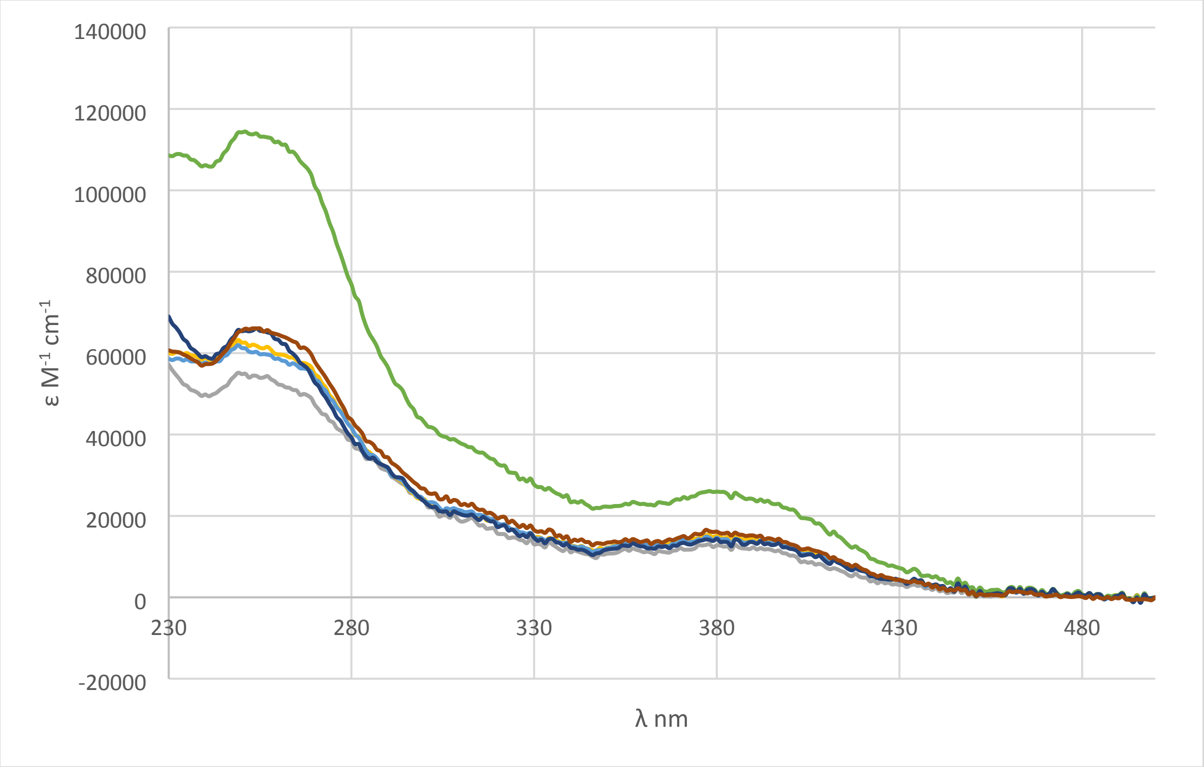

Figure S4. Electronic spectra of the complexes. Λ-**Ir**^{S,R}**2** (brown); Δ-**Ir**^{S,R}**2** (grey); Λ-**Ir**^{S,R}**3** (yellow); Δ-**Ir**^{S,R}**3** (light blue); Λ-**Ir**^{S,R}**4** (green); Λ-**Ir**^{S,R}**5** (dark blue)

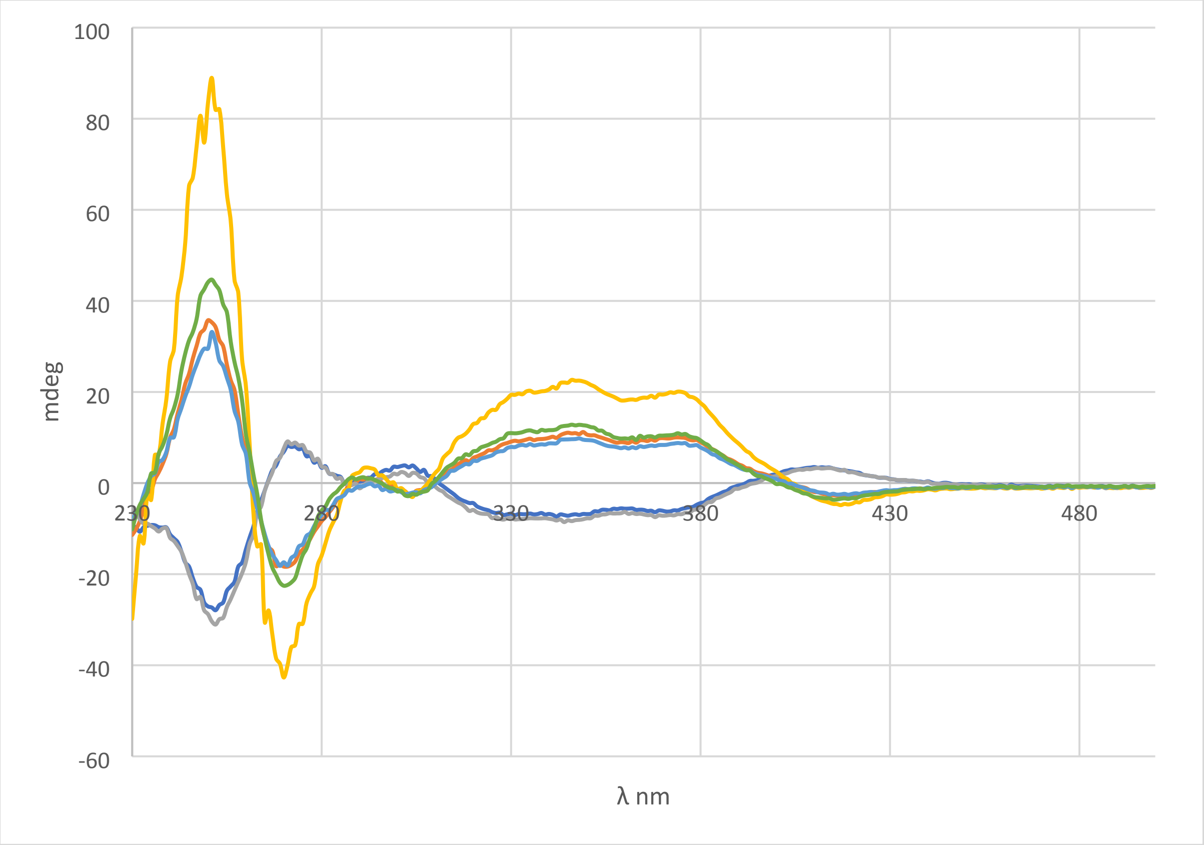

Figure S5. CD spectra of the complexes. Λ-**Ir**^{S,R}**2** (green); Δ-**Ir**^{S,R}**2** (dark blue); Λ-**Ir**^{S,R}**3** (orange); Δ-**Ir**^{S,R}**3** (grey); Λ-**Ir**^{S,R}**4** (yellow); Λ-**Ir**^{S,R}**5** (light blue)

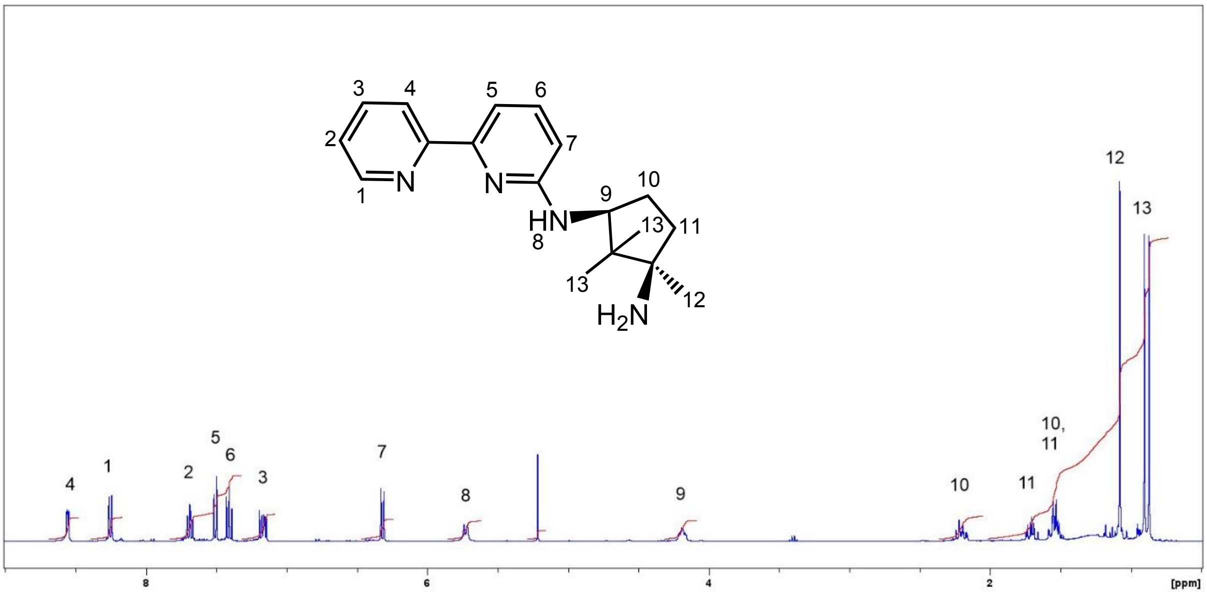

Figure S6. ^{1}H NMR (400 MHz, $CDCl_3$) of *N*¹-([2,2'-bipyridin]-6-yl)-2,2,3-trimethylcyclopentane-1*S*,3*R*-diamine, ^{S,R}**L**.

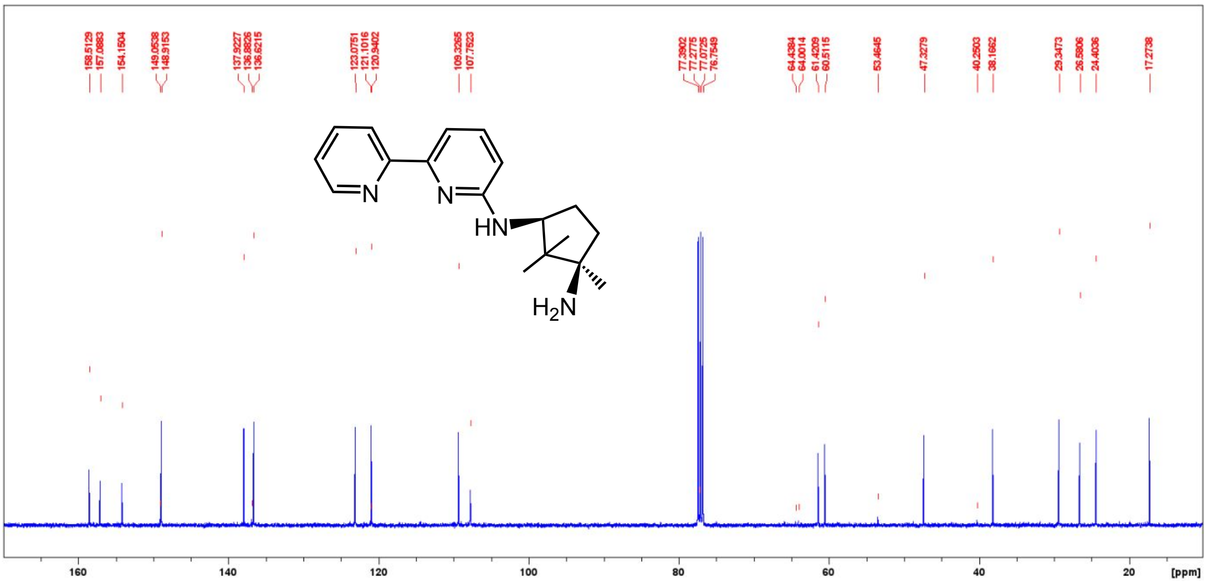

Figure S7. $^{13}C\{^{1}H\}$ NMR (125 MHz, $CDCl_3$) of *N*¹-([2,2'-bipyridin]-6-yl)-2,2,3-trimethylcyclopentane-1*S*,3*R*-diamine, ^{S,R}**L**.

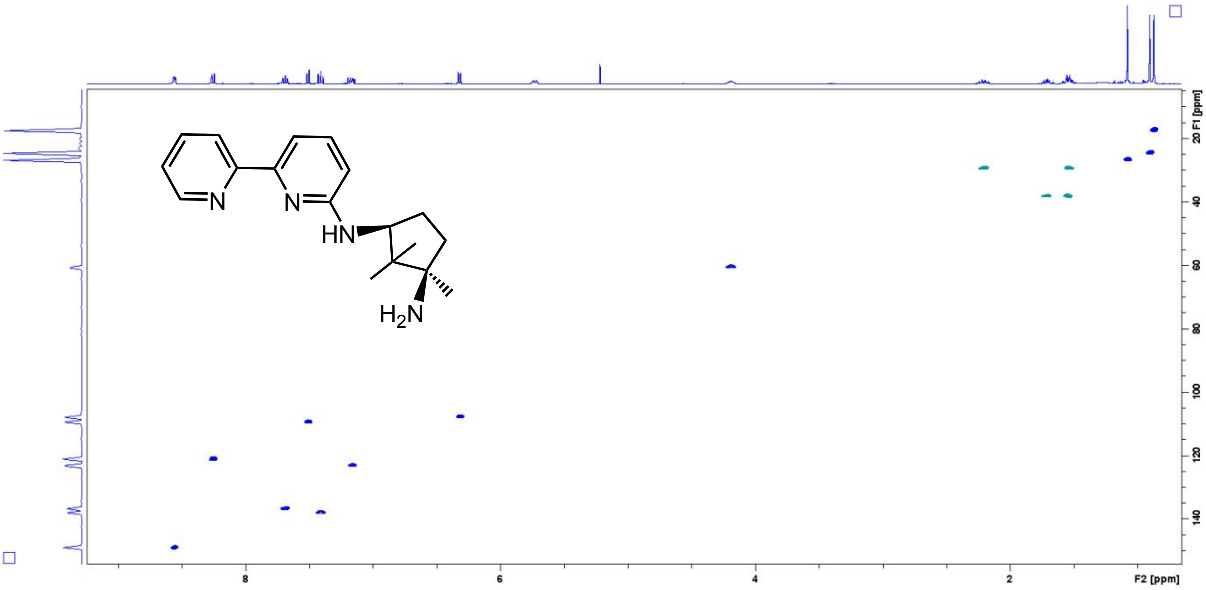

Figure S8. ^{1}H,^{13}C HSQC NMR (400 MHz, $CDCl_3$) of *N*[1]-([2,2'-bipyridin]-6-yl)-2,2,3-trimethylcyclopentane-1*S*,3*R*-diamine, ^{S,R}**L**.

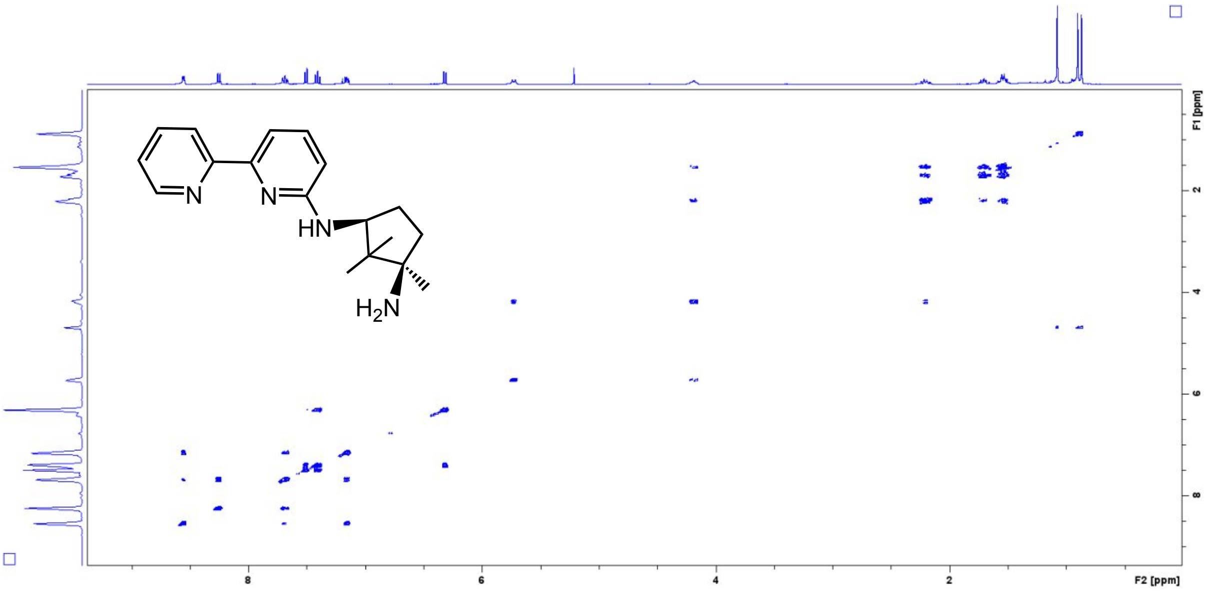

Figure S9. ^{1}H,^{1}H COSY NMR (400 MHz, $CDCl_3$) of *N*[1]-([2,2'-bipyridin]-6-yl)-2,2,3-trimethylcyclopentane-1*S*,3*R*-diamine, ^{S,R}**L**.

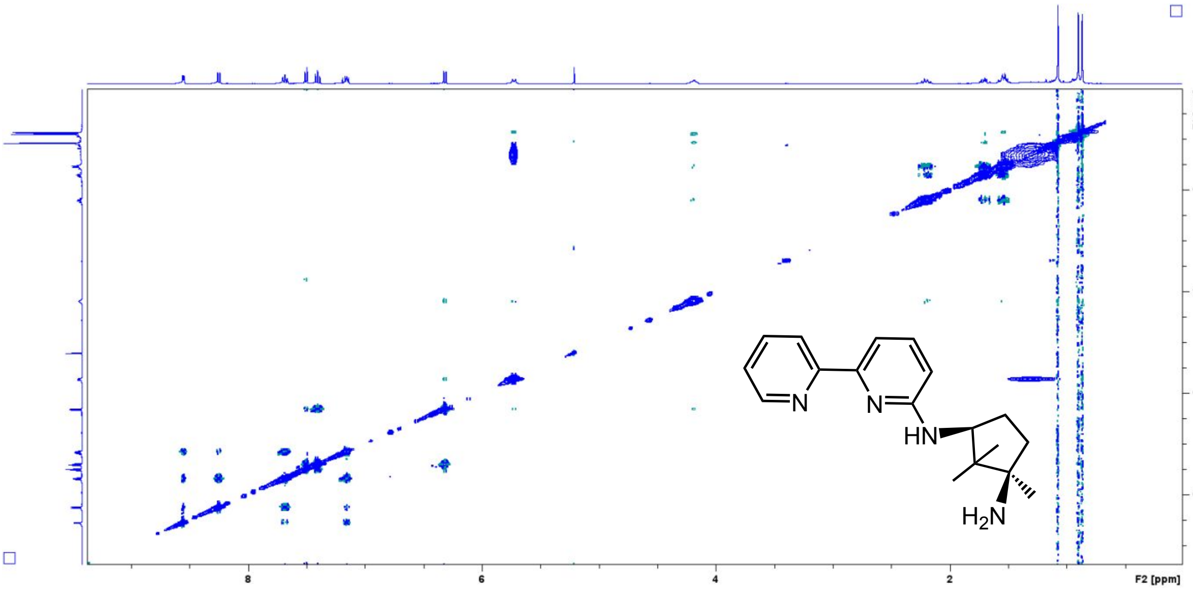

Figure S10. ^{1}H,^{1}H NOESY NMR (400 MHz, $CDCl_3$) of *N*^{1}-([2,2'-bipyridin]-6-yl)-2,2,3-trimethylcyclopentane-1*S*,3*R*-diamine, ^{S,R}**L**.

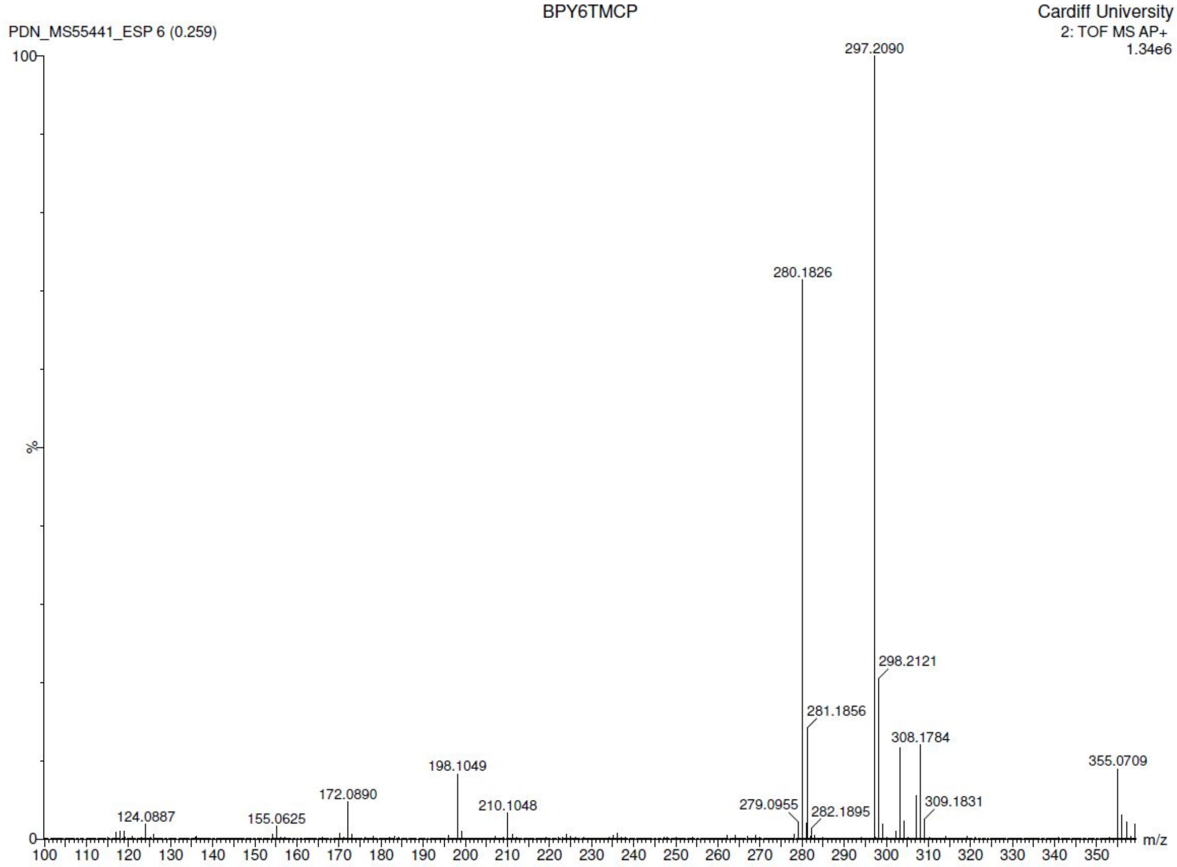

	Mass	Calc. Mass	mDa	PPM	DBE	i-FIT	Norm	Conf(%)	Formula
Minimum:					-1.5				
Maximum:			5.0	5.0	100.0				
	297.2090	297.2079	1.1	3.7	8.5	820.4	n/a	n/a	C18 H25 N4

Figure S11. HRMS of *N*[1]-([2,2'-bipyridin]-6-yl)-2,2,3-trimethylcyclopentane-1*S*,3*R*-diamine, ^{S,R}**L**.

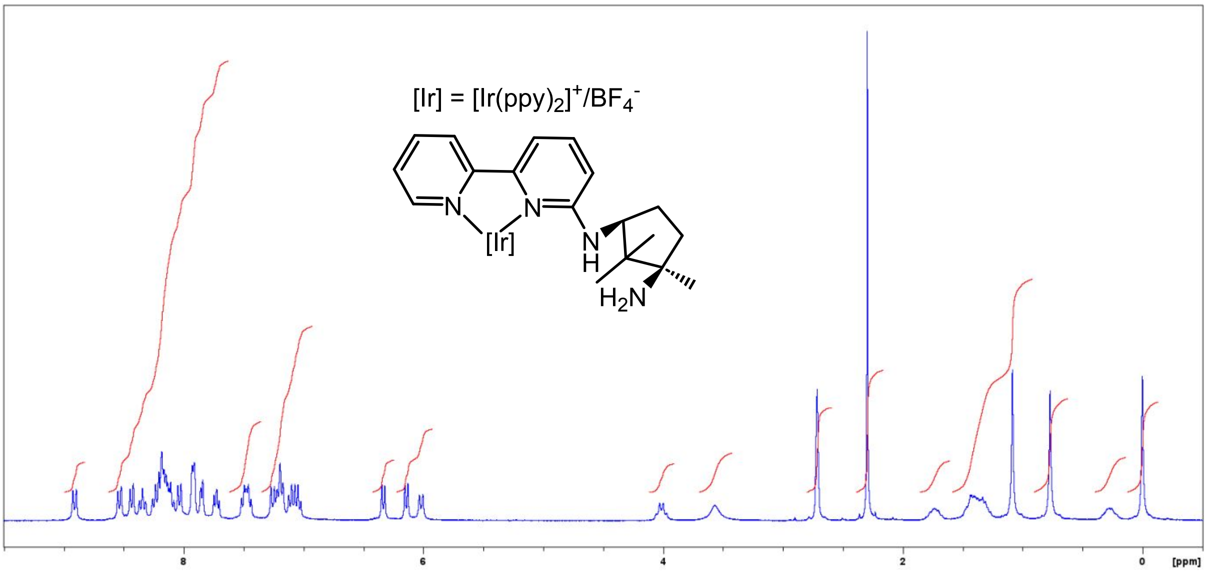

Figure S12. ^{1}H NMR (400 MHz, DMSO-d$_6$) of Λ-**Ir**^{S,R}**1**.

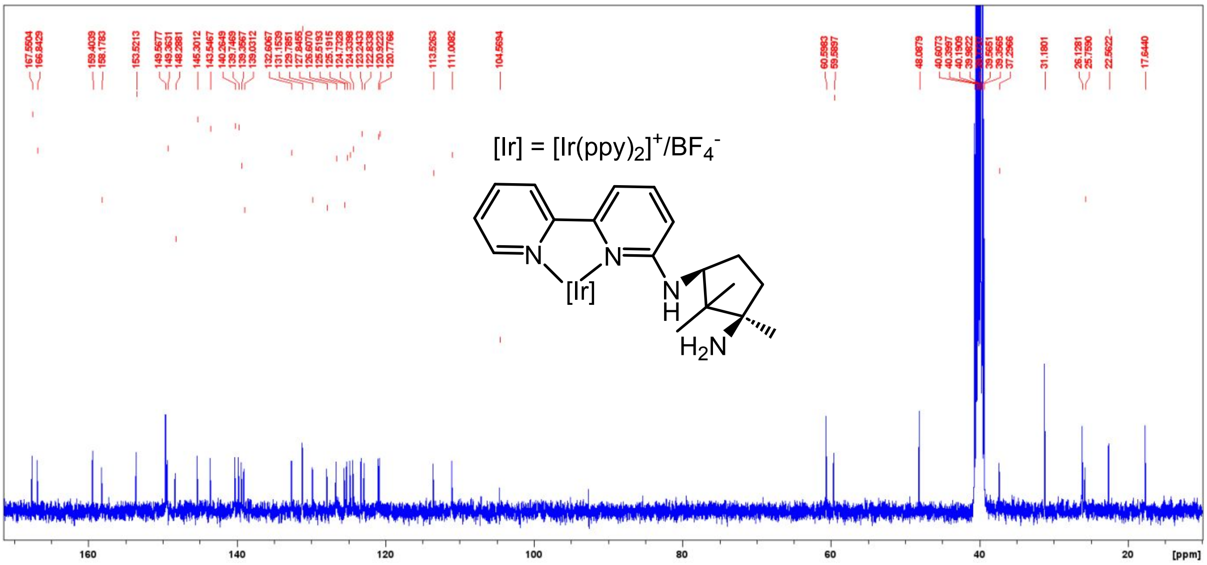

Figure S13. ^{13}C{^{1}H} NMR (125 MHz, DMSO-d$_6$) of Λ-**Ir**^{S,R}**1**.

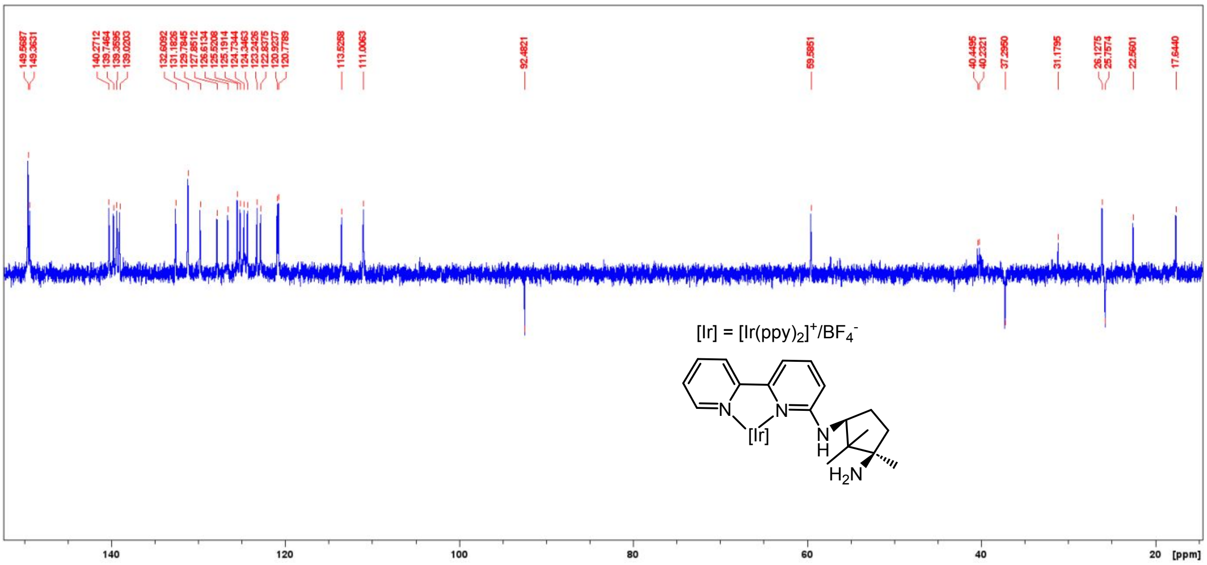

Figure S14. ^{13}C DEPT NMR (125 MHz, DMSO-d_6) of Λ-**Ir**^{S,R}**1**.

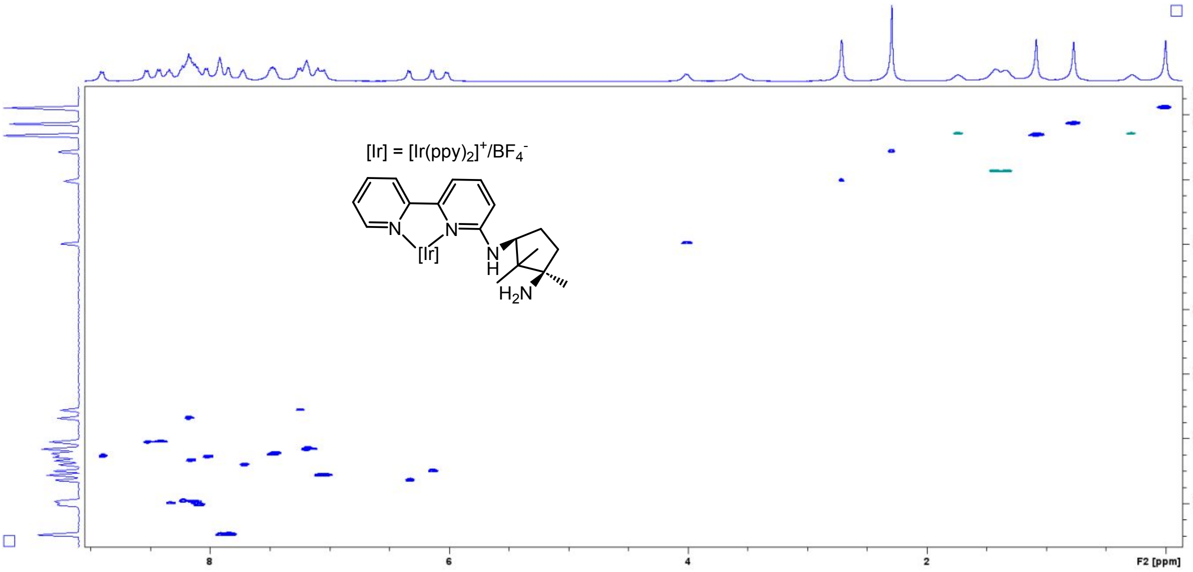

Figure S15. ^{1}H,^{13}C HSQC NMR (400 MHz, DMSO-d_6) of Λ-**Ir**^{S,R}**1**.

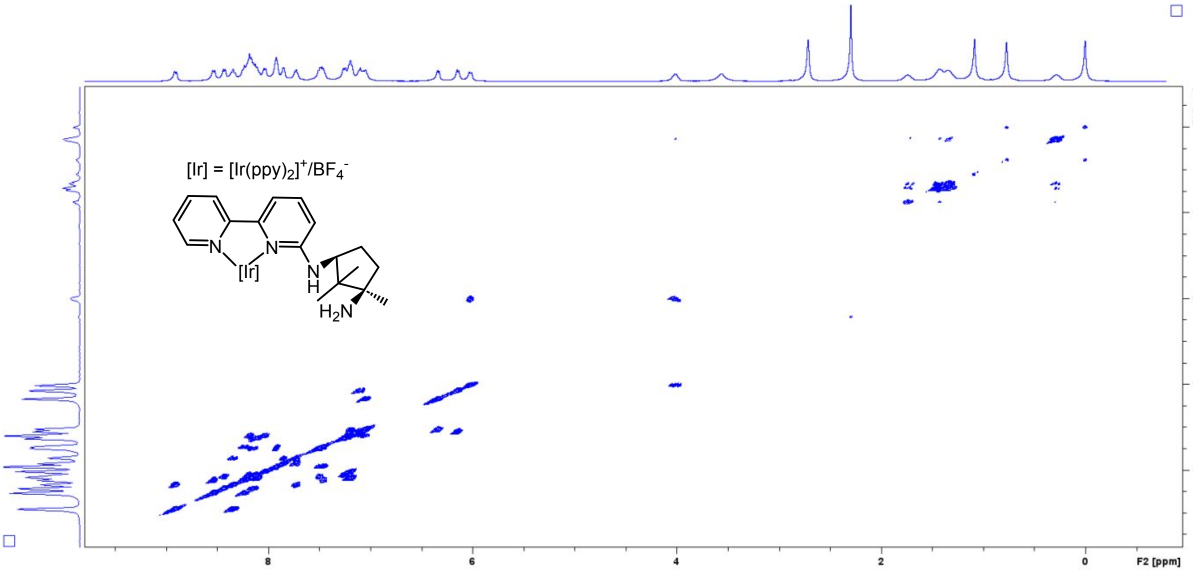

Figure S16. $^{1}H,^{1}H$ COSY NMR (400 MHz, DMSO-d_6) of Λ-**Ir**^{S,R}**1**.

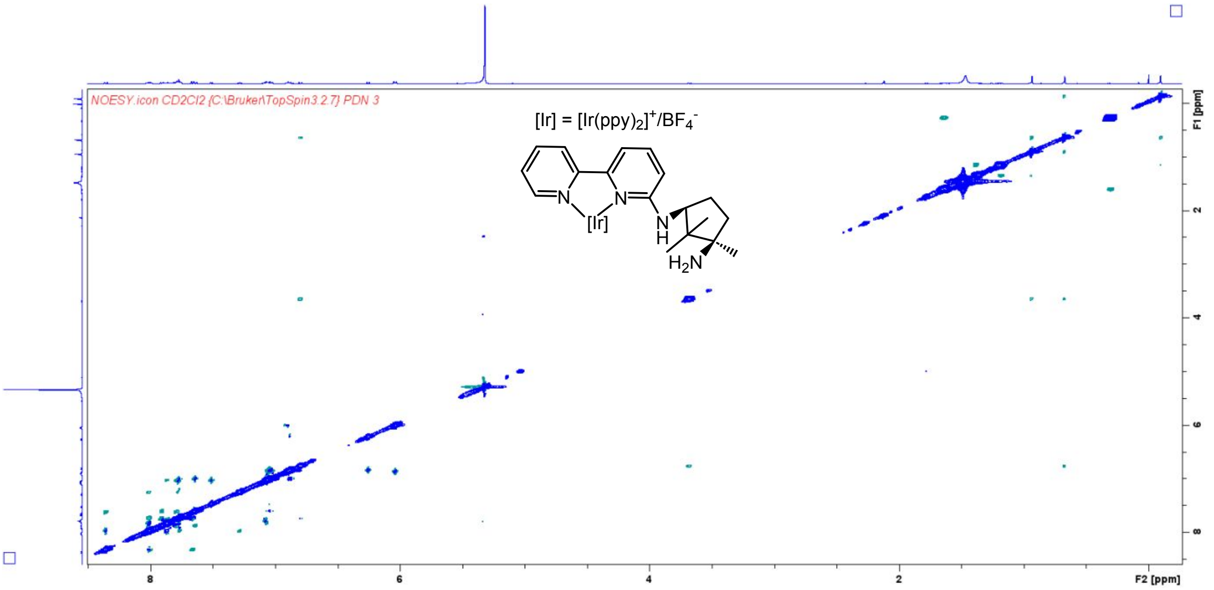

Figure S17. $^{1}H,^{1}H$ NOESY NMR (400 MHz, DMSO-d_6) of Λ-**Ir**^{S,R}**1**.

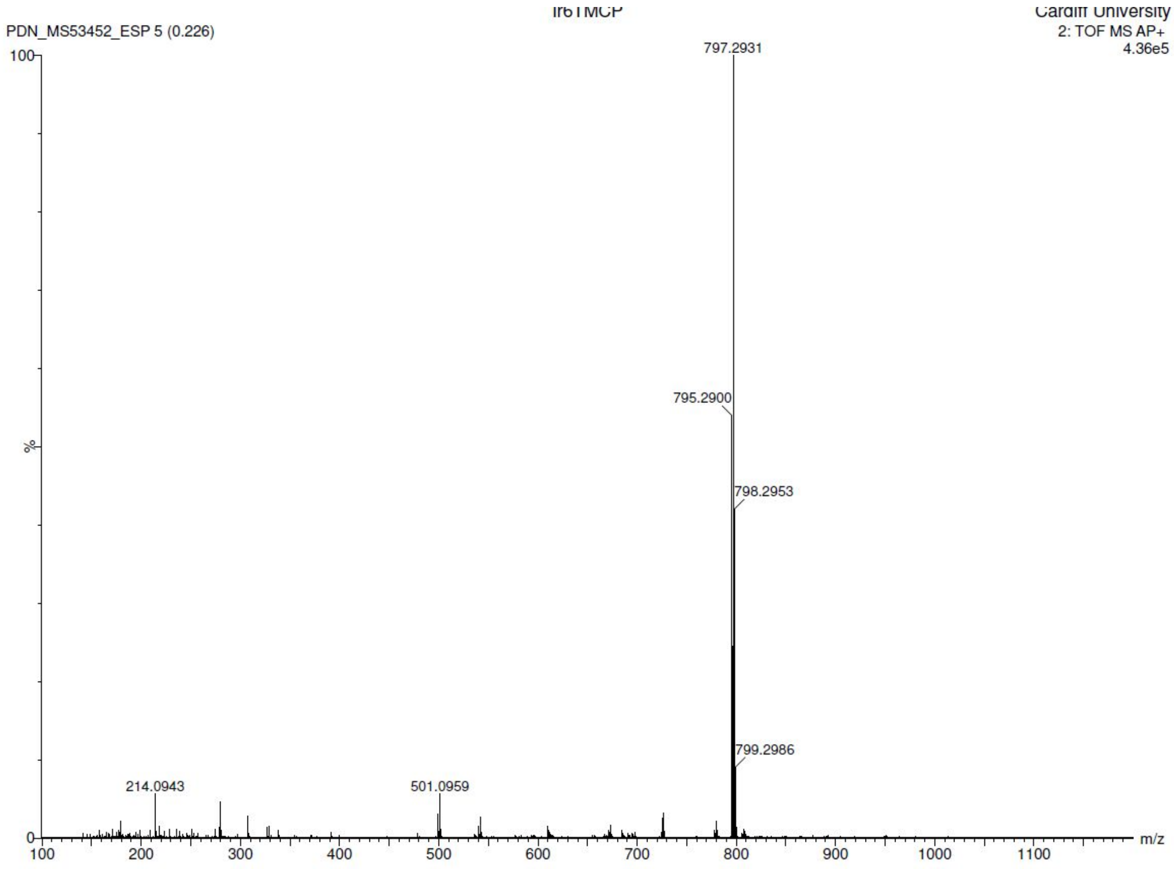

Minimum:				-1.5				
Maximum:		5.0	10.0	50.0				
Mass	Calc. Mass	mDa	PPM	DBE	i-FIT	Norm	Conf(%)	Formula
797.2931	797.2944	-1.3	-1.6	25.0	889.7	n/a	n/a	C40 H40 N6 193Ir

Figure S18. HRMS of Λ-**Ir**^{S,R}**1**.

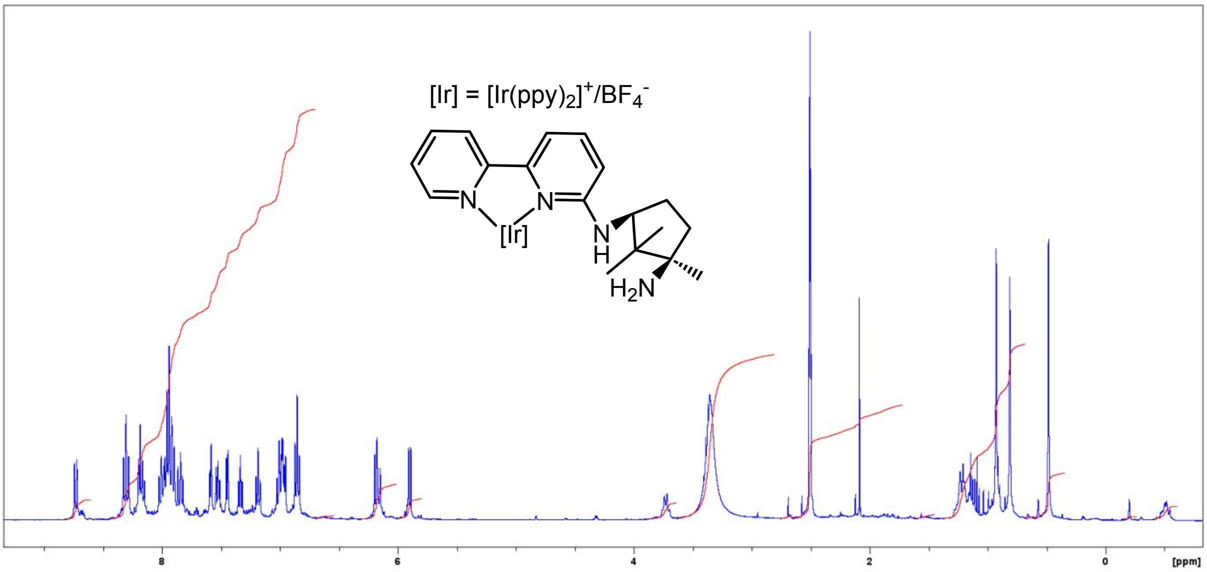

Figure S19. ^{1}H NMR (400 MHz, DMSO-d_6) of Δ-**Ir**^{S,R}**1**.

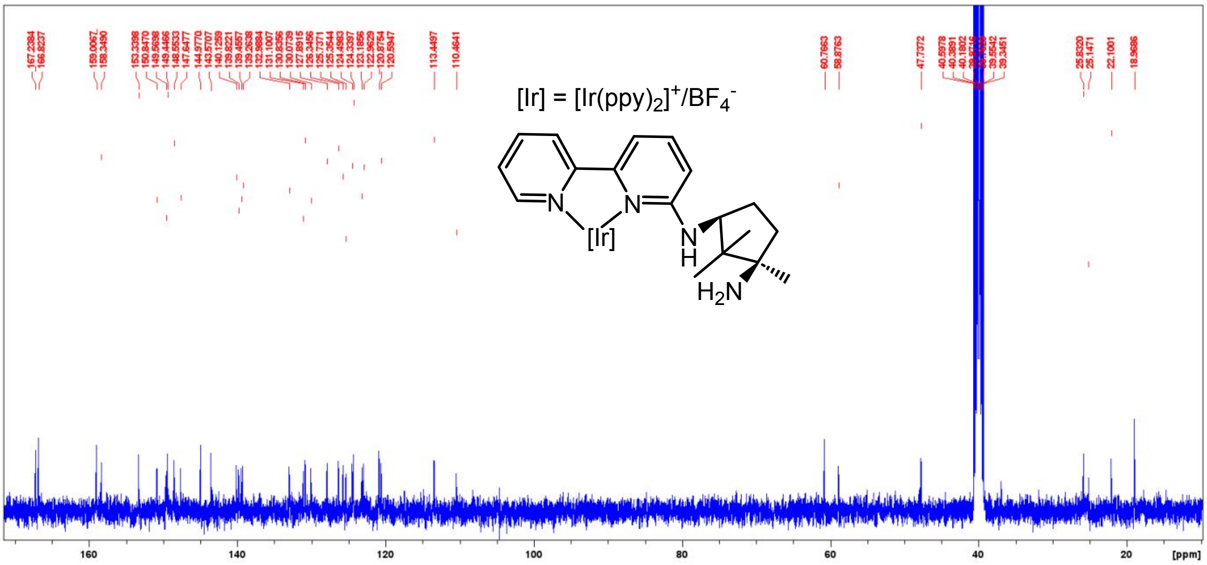

Figure S20. $^{13}C\{^{1}H\}$ NMR (125 MHz, DMSO-d_6) of Δ-**Ir**^{S,R}**1**.

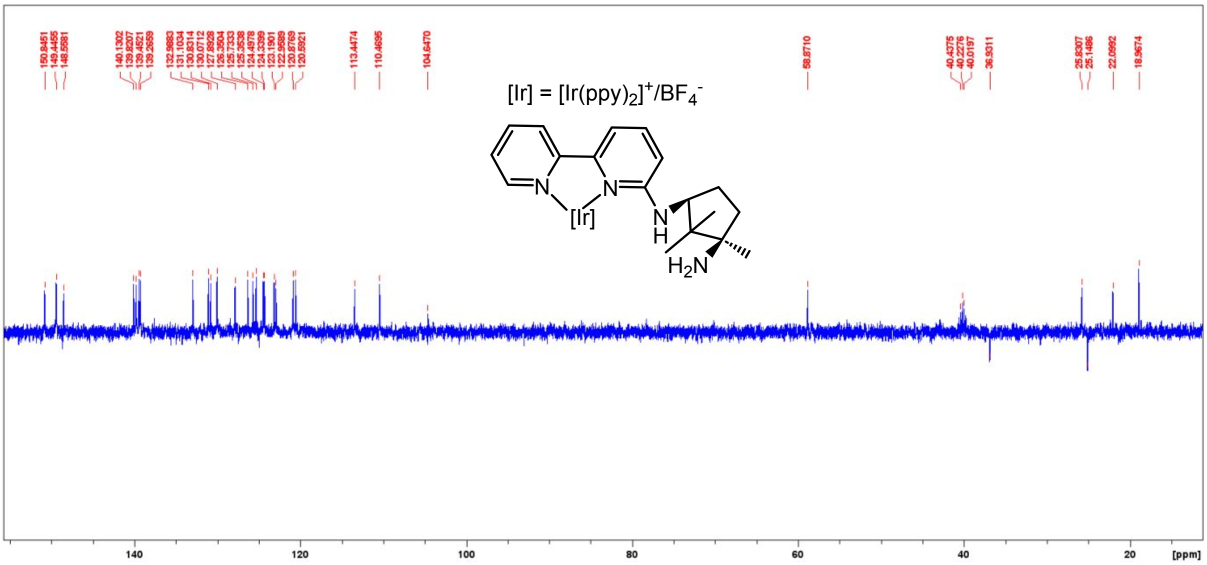

Figure S21. ^{13}C DEPT NMR (125 MHz, DMSO-d_6) of Δ-**Ir**^{S,R}**1**.

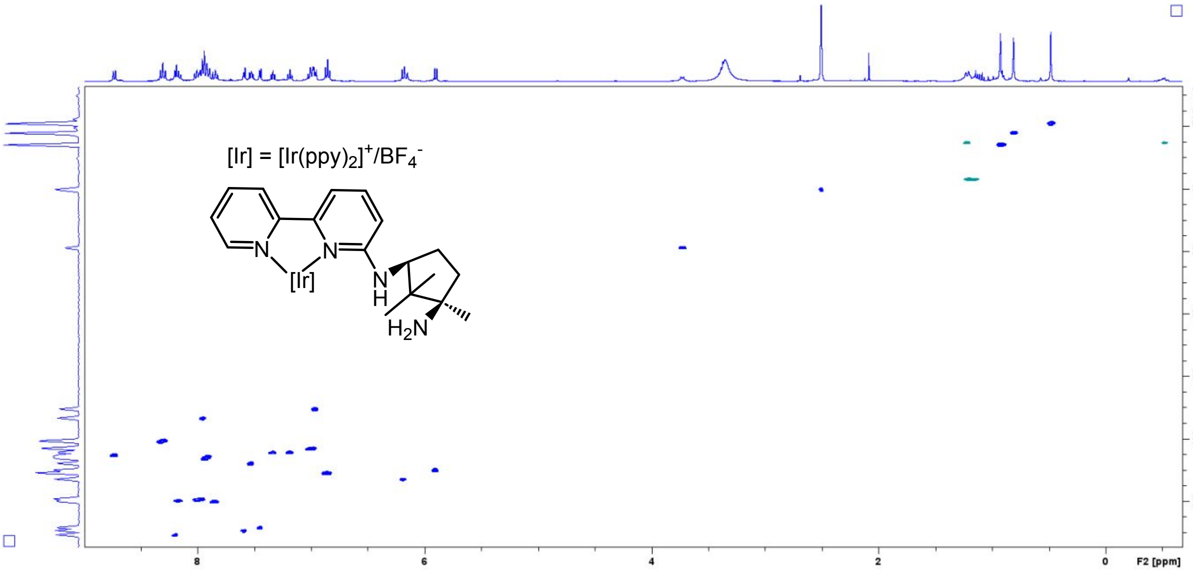

Figure S22. $^{1}H,^{13}C$ HSQC NMR (400 MHz, DMSO-d_6) of Δ-**Ir**^{S,R}**1**.

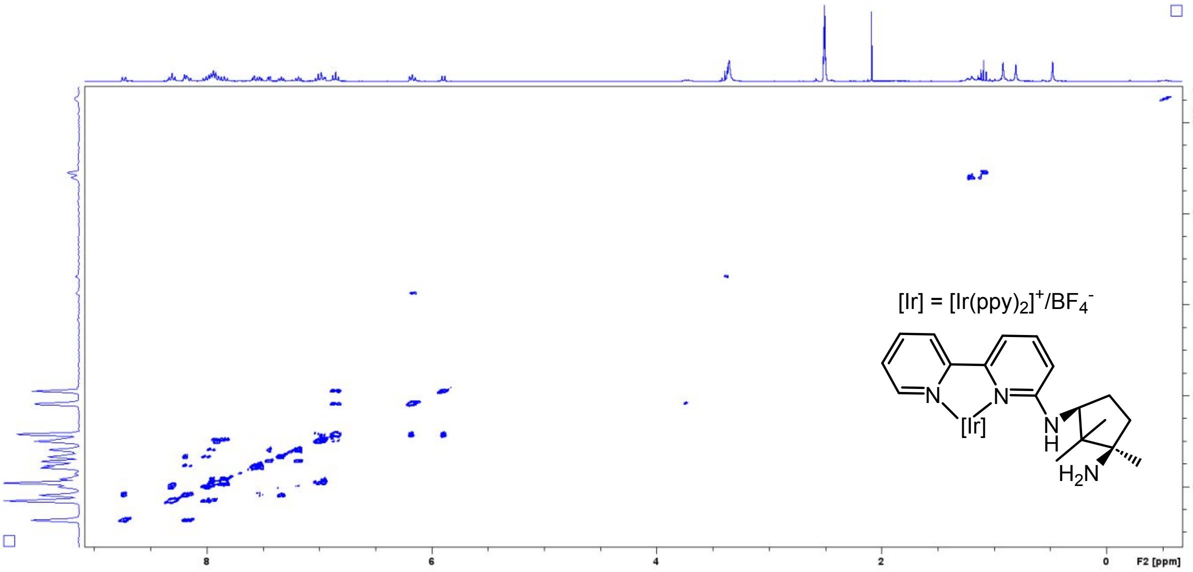

Figure S23. $^{1}H,^{1}H$ COSY NMR (400 MHz, DMSO-d_6) of Δ-**Ir**^{S,R}**1**.

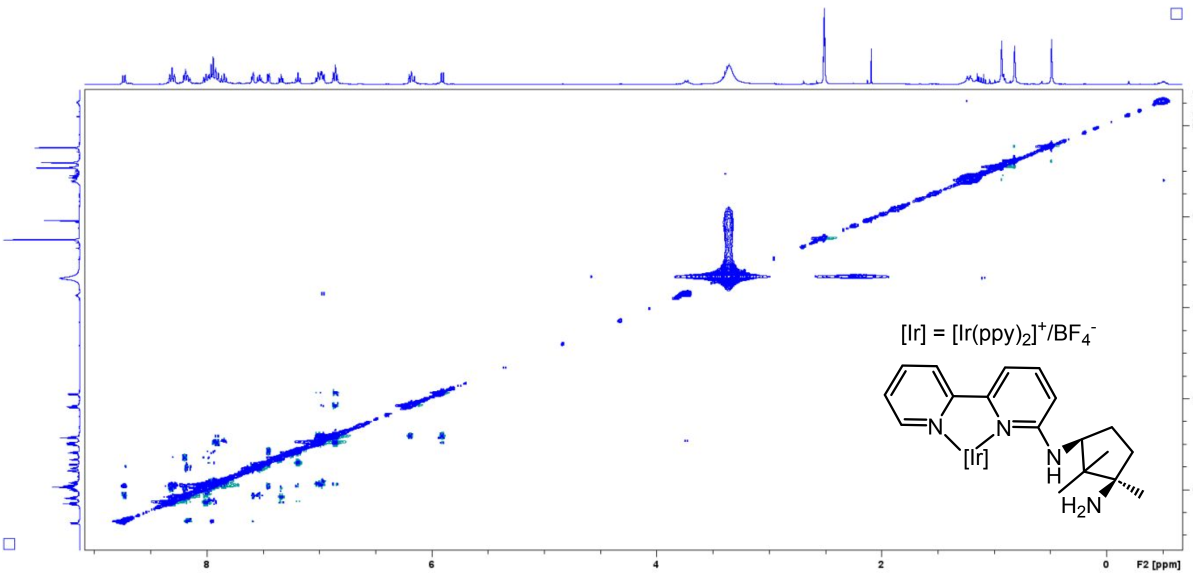

Figure S24. $^{1}H,^{1}H$ NOESY NMR (400 MHz, DMSO-d_6) of Δ-**Ir**^{S,R}**1**.

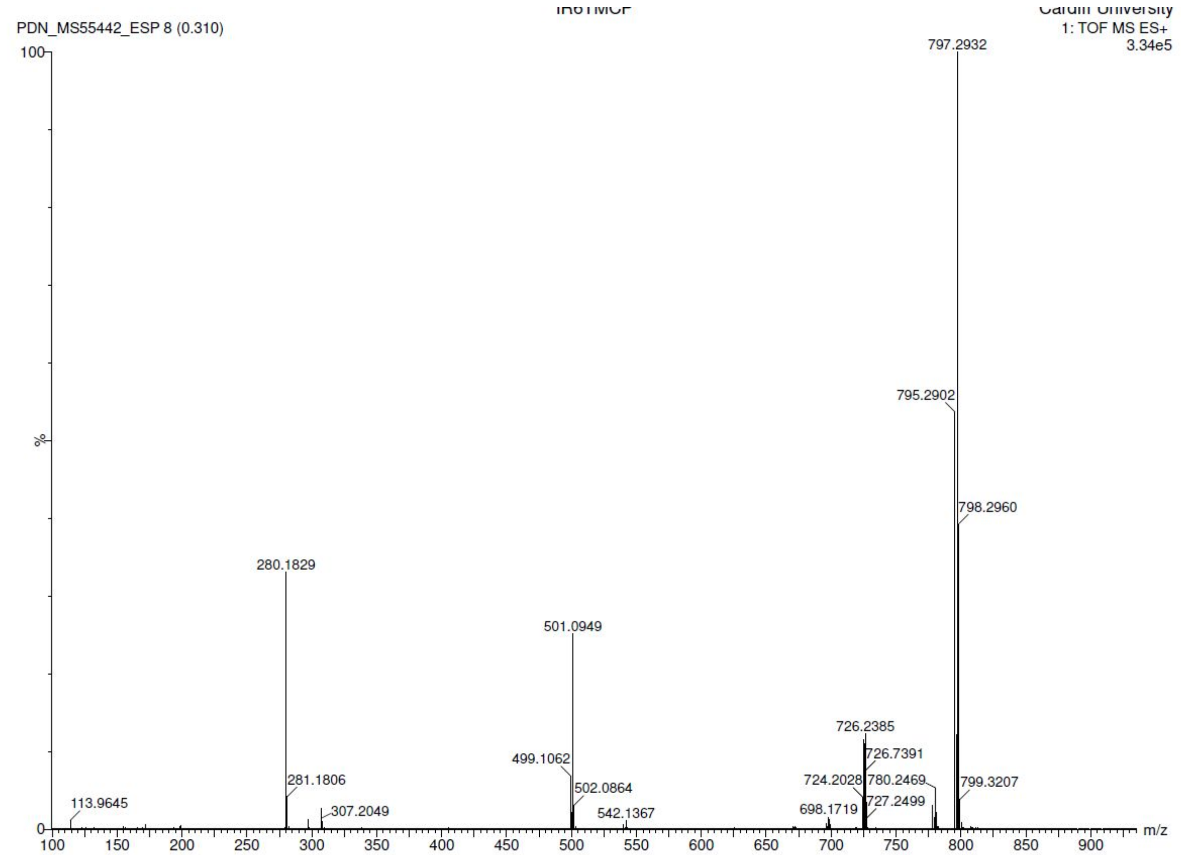

Minimum:				-1.5				
Maximum:		5.0	5.0	100.0				
Mass	Calc. Mass	mDa	PPM	DBE	i-FIT	Norm	Conf(%)	Formula
797.2932	797.2944	-1.2	-1.5	25.0	428.8	n/a	n/a	C40 H40 N6 193Ir

Figure S25. HRMS of Δ-**Ir**^{S,R}**1**.

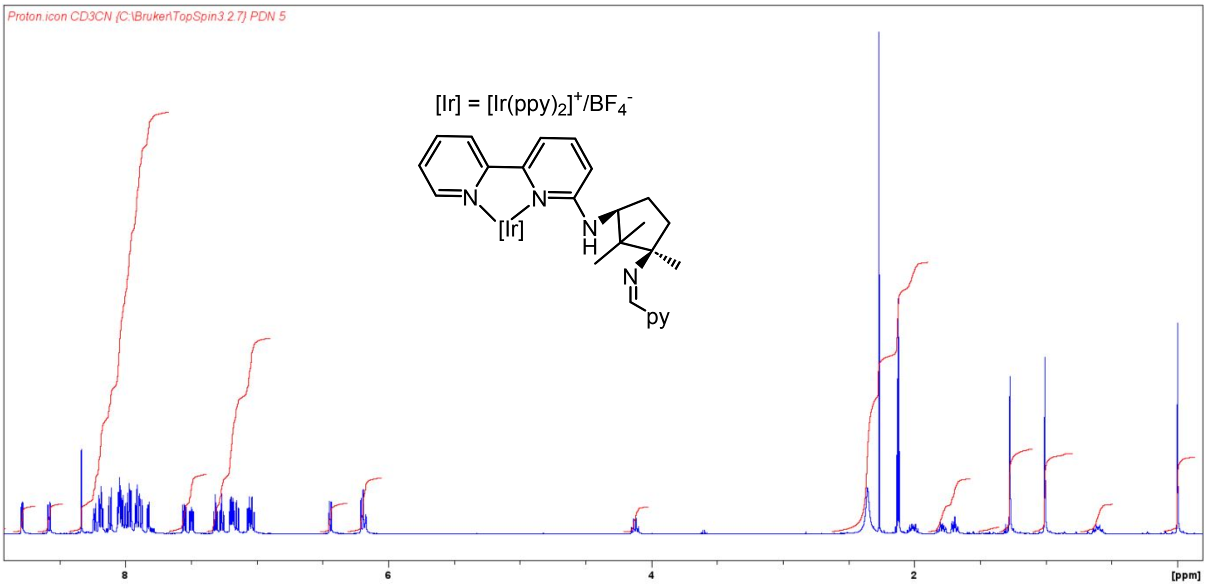

Figure S26. ¹H NMR (500 MHz, MeCN-d_3) of Λ-**Ir**^{S,R}**2**.

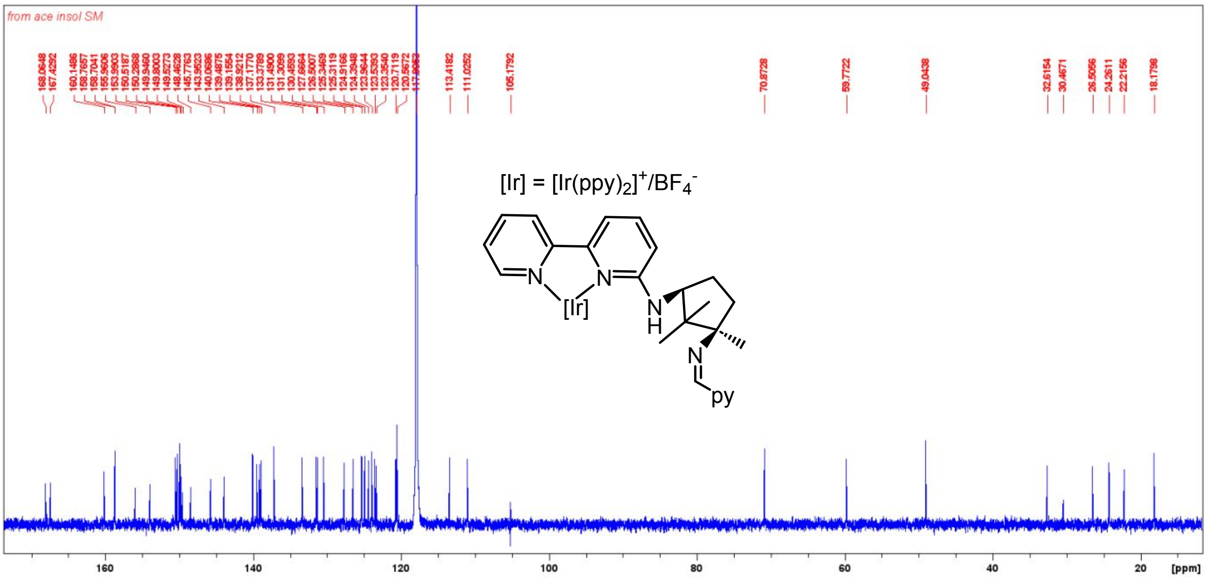

Figure S27. $^{13}C\{^{1}H\}$ NMR (100 MHz, MeCN-d_3) of Λ-**Ir**^{S,R}**2**.

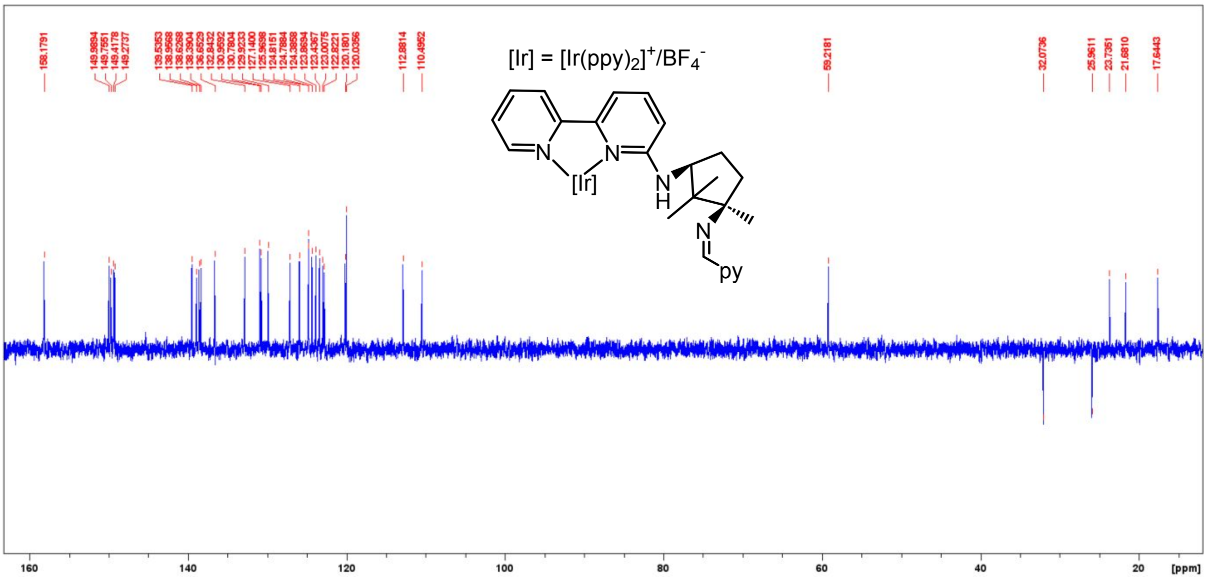

Figure S28. ^{13}C DEPT NMR (100 MHz, MeCN-d$_3$) of Λ-**Ir**^{S,R}**2**.

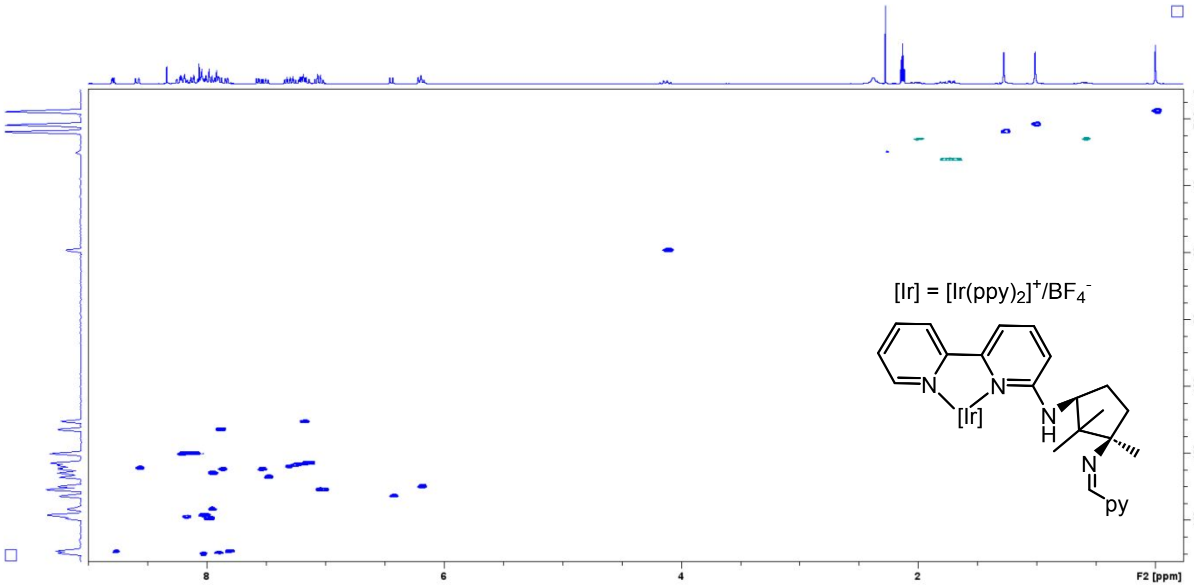

Figure S29. ^{1}H,^{13}C HSQC NMR (500 MHz, MeCN-d$_3$) of Λ-**Ir**^{S,R}**2**.

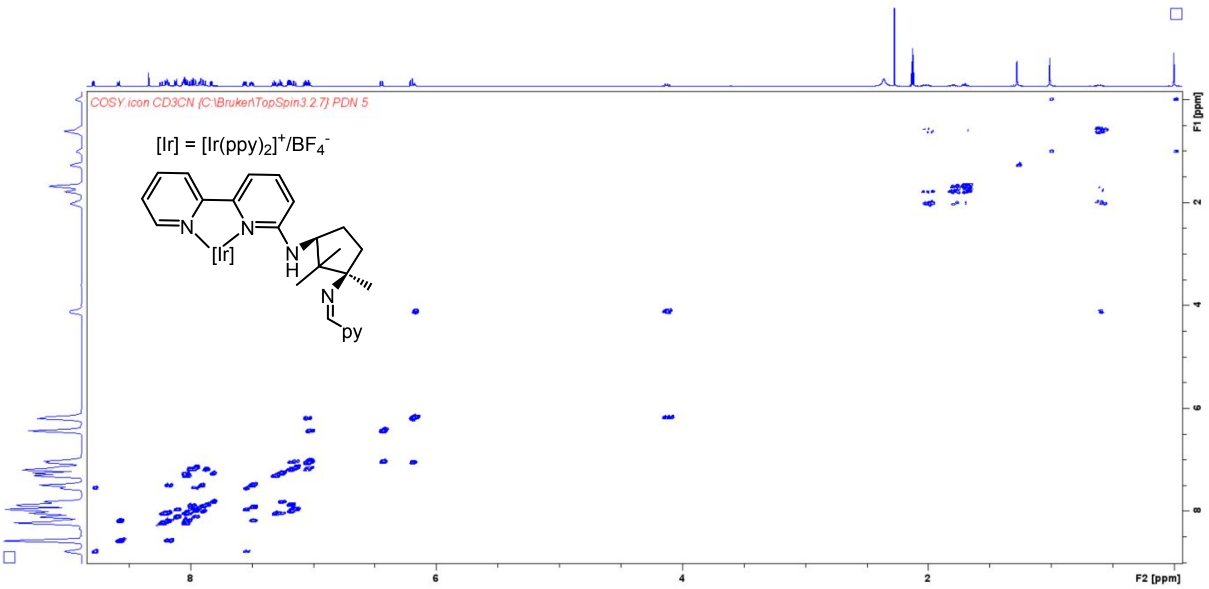

Figure S30. $^{1}H,^{1}H$ COSY NMR (500 MHz, MeCN-d_3) of Λ-**Ir**^{S,R}**2**.

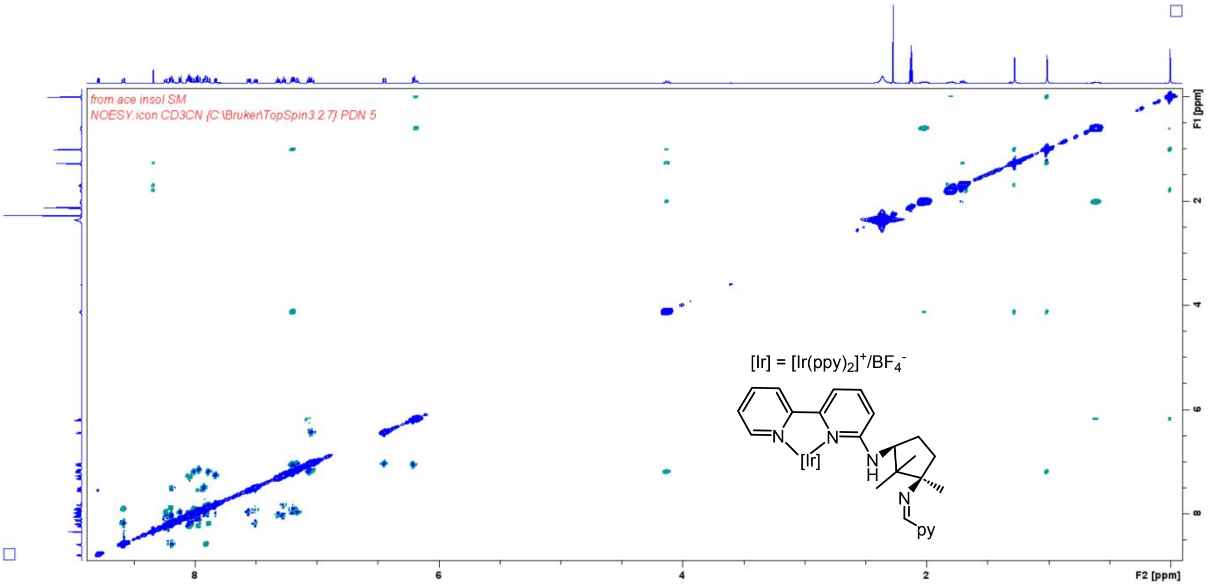

Figure S31. $^{1}H,^{1}H$ NOESY NMR (500 MHz, MeCN-d_3) of Λ-**Ir**^{S,R}**2**.

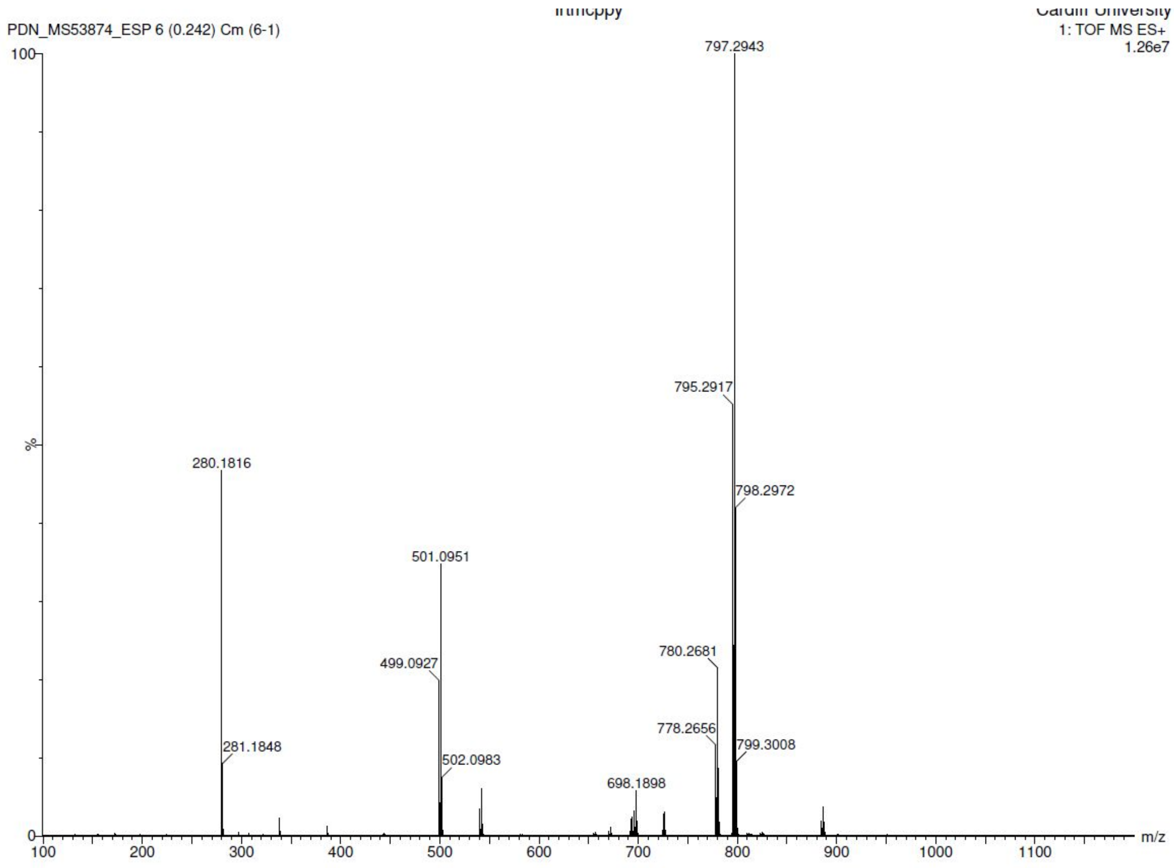

Minimum:				-1.5				
Maximum:		5.0	20.0	50.0				
Mass	Calc. Mass	mDa	PPM	DBE	i-FIT	Norm	Conf(%)	Formula
886.3206	886.3209	-0.3	-0.3	30.0	782.2	n/a	n/a	C46 H43 N7 Ir

Figure S32. HRMS of Λ-$\mathbf{Ir}^{S,R}\mathbf{2}$.

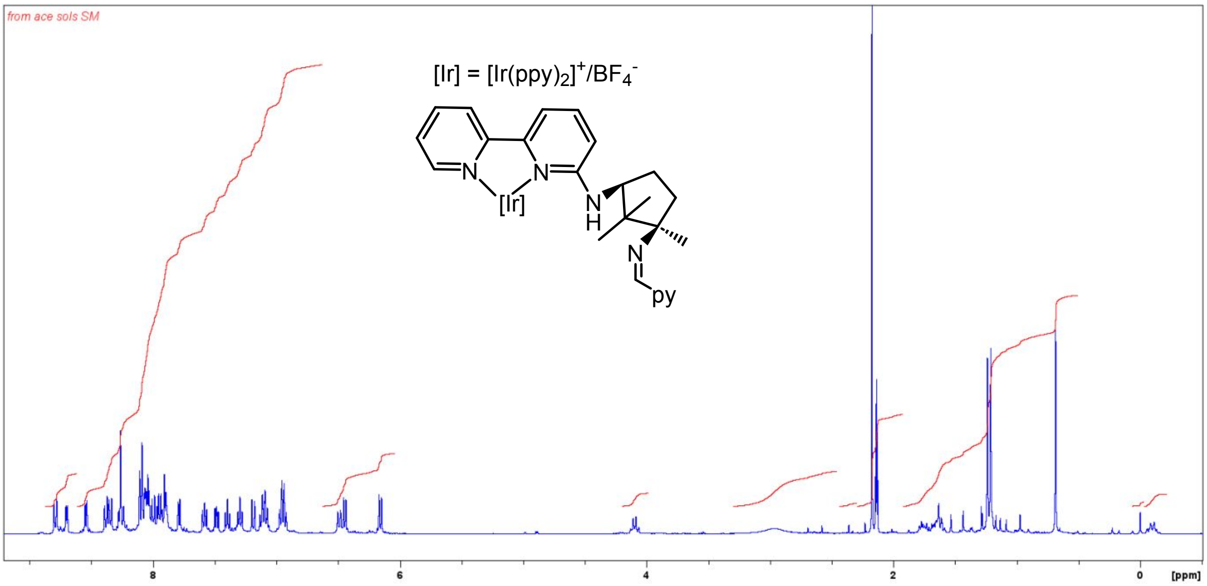

Figure S33. ^{1}H NMR (400 MHz, acetone-d_6) of Δ-$\mathbf{Ir}^{S,R}\mathbf{2}$.

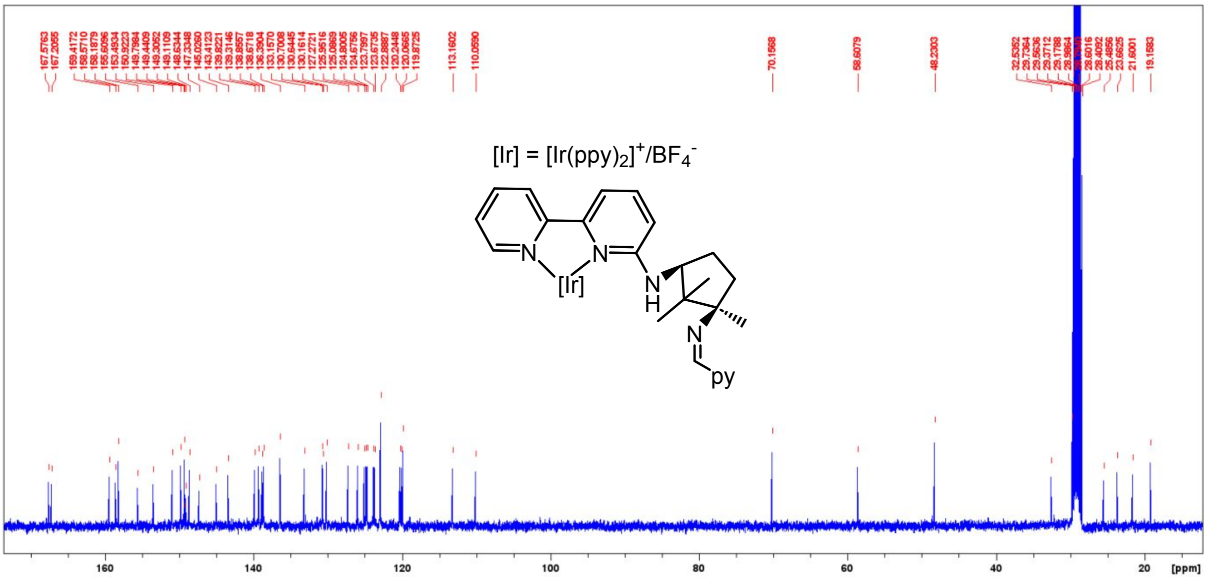

Figure S34. $^{13}C\{^{1}H\}$ NMR (100 MHz, acetone-d_6) of Δ-**Ir**^{S,R}**2**.

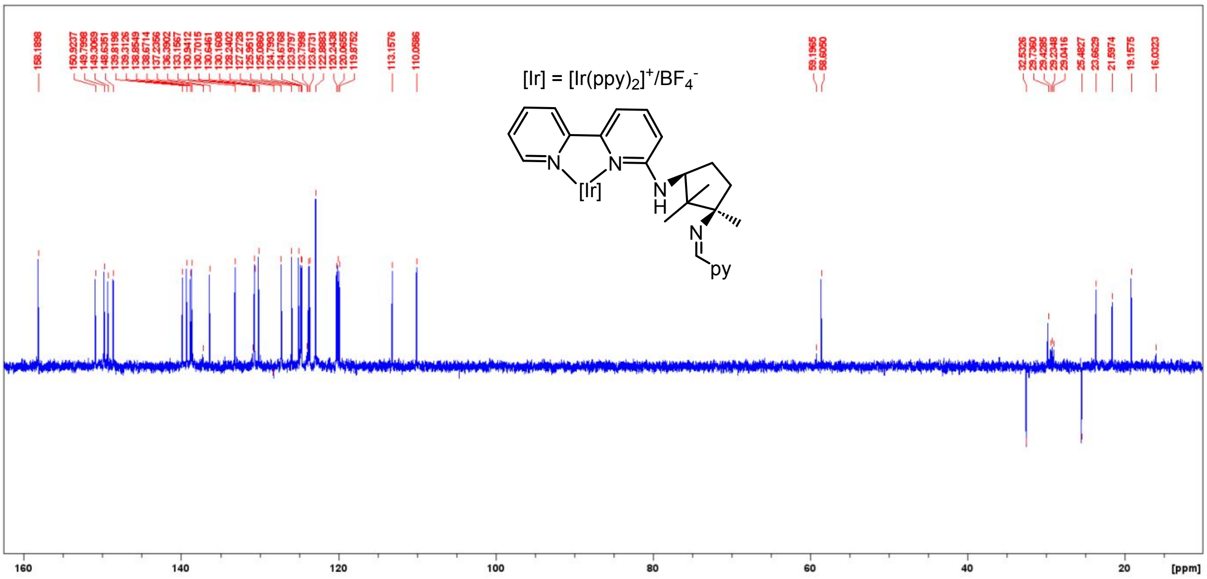

Figure S35. ^{13}C DEPT NMR (100 MHz, acetone-d_6) of Δ-**Ir**^{S,R}**2**.

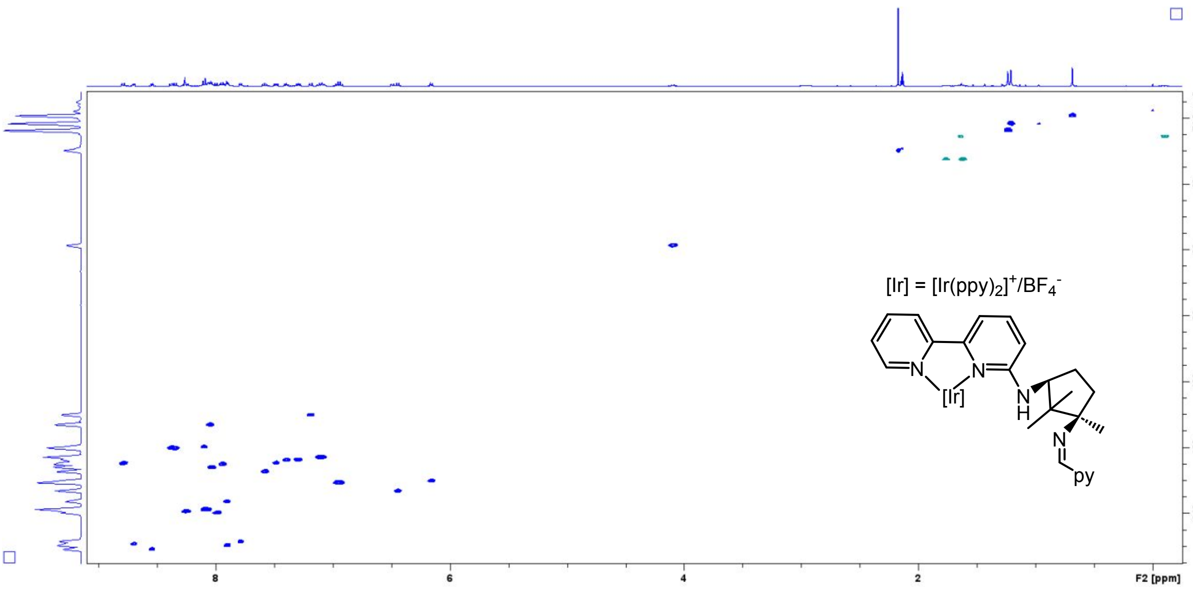

Figure S36. $^{1}H,^{13}C$ HSQC NMR (100 MHz, acetone-d_6) of Δ-**Ir*S,R*2**.

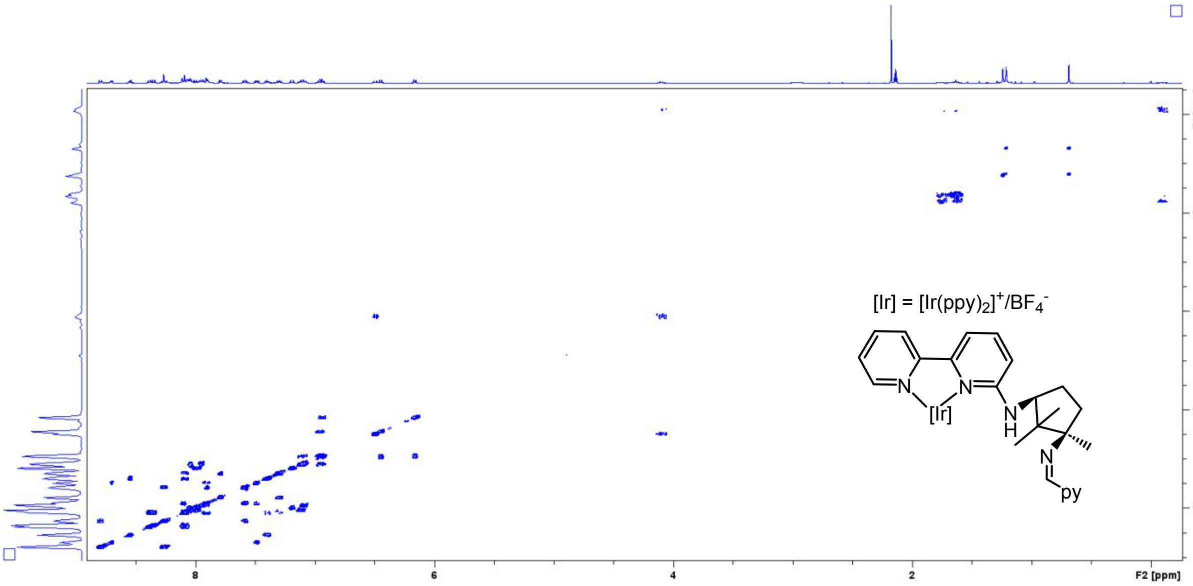

Figure S37. $^{1}H,^{1}H$ COSY NMR (400 MHz, acetone-d_6) of Δ-**Ir*S,R*2**.

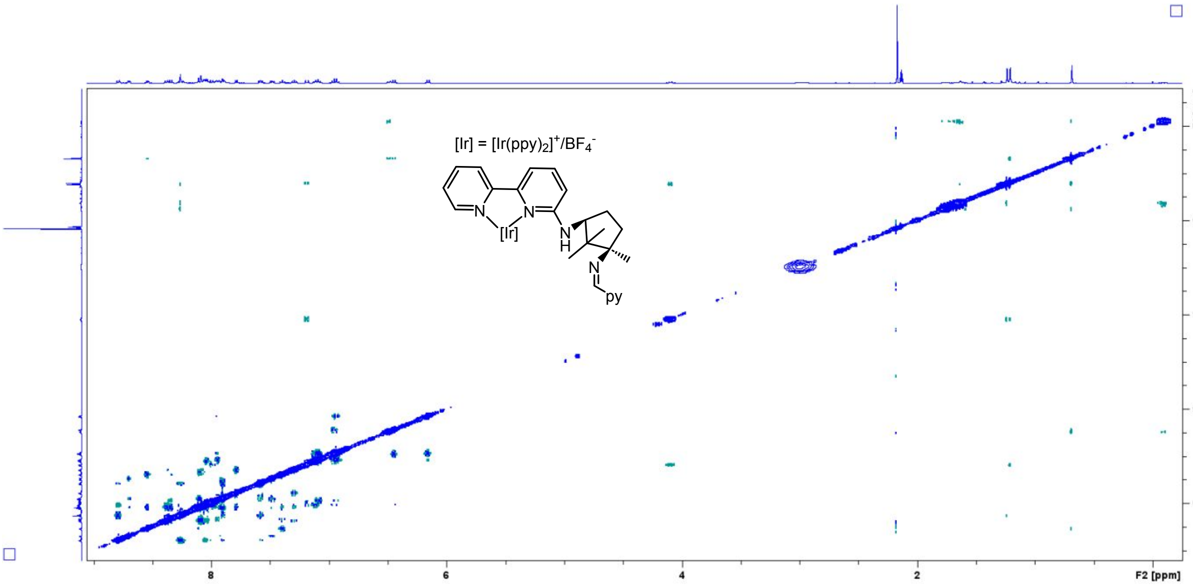

Figure S38. $^{1}H,^{1}H$ NOESY NMR (400 MHz, acetone-d_6) of Δ-**Ir**^{S,R}**2**.

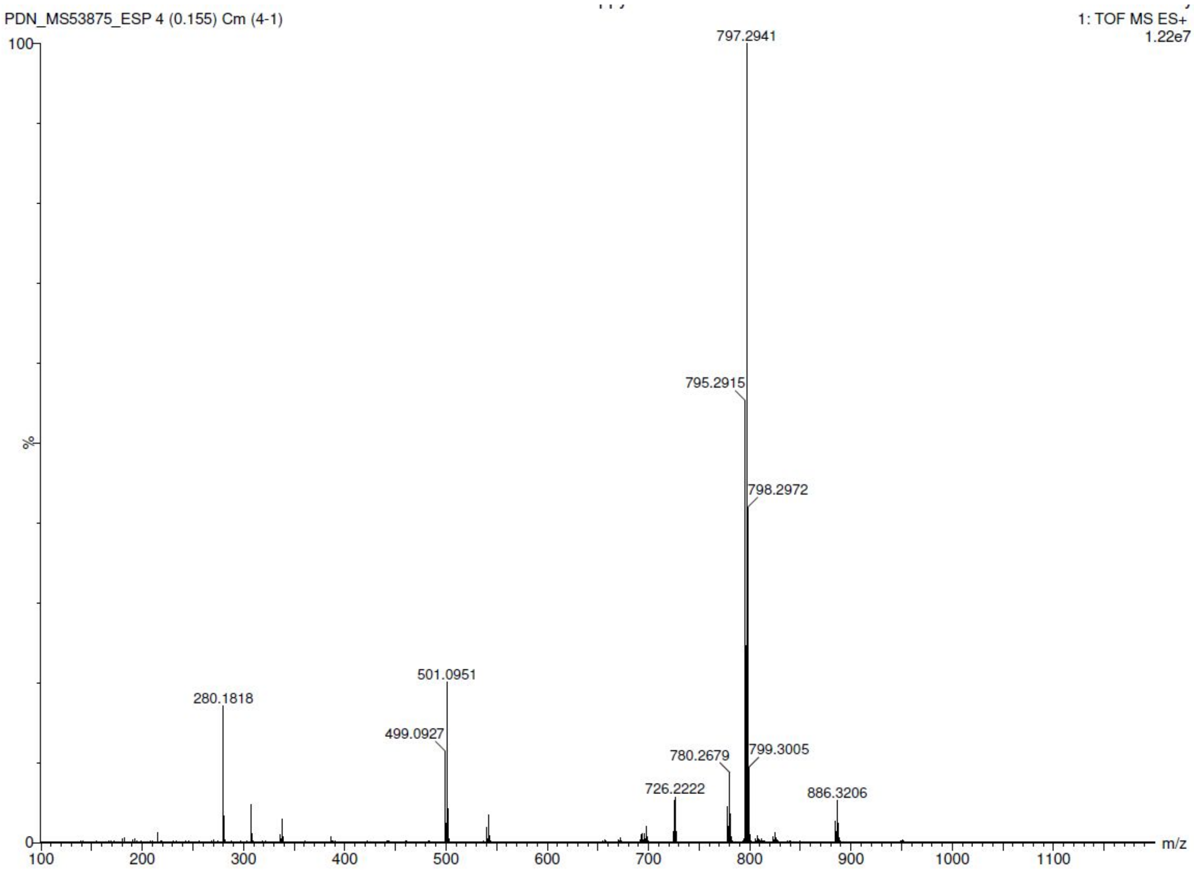

Minimum:				-1.5				
Maximum:		5.0	20.0	50.0				
Mass	Calc. Mass	mDa	PPM	DBE	i-FIT	Norm	Conf(%)	Formula
886.3206	886.3209	-0.3	-0.3	30.0	914.5	n/a	n/a	C46 H43 N7 Ir

Figure S39. HRMS of Δ-**Ir**^{S,R}**2**.

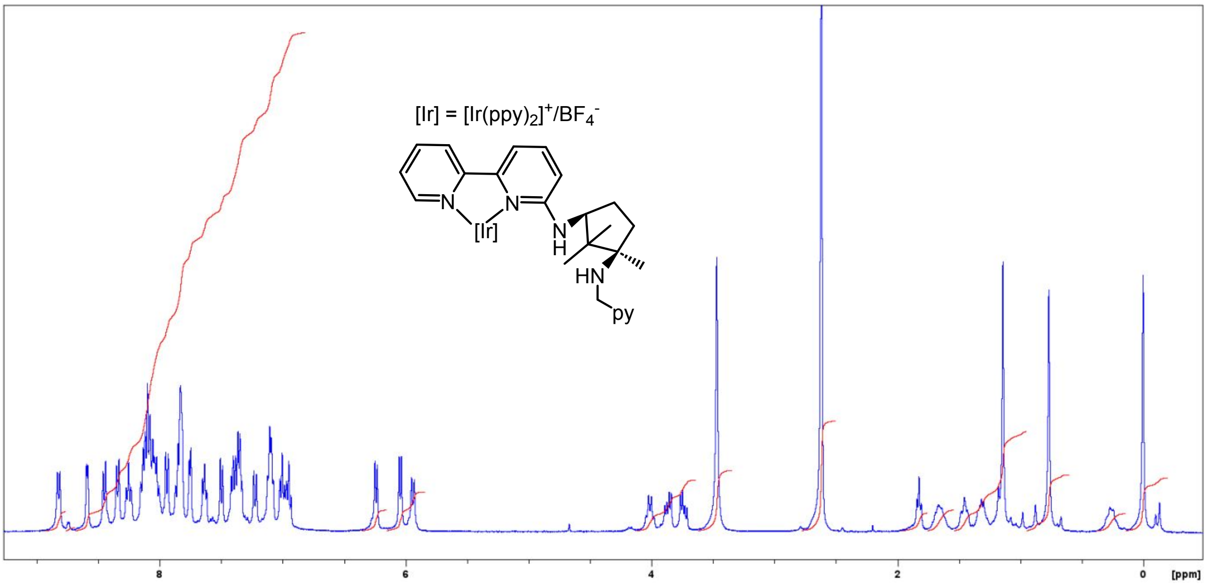

Figure S40. ^{1}H NMR (400 MHz, DMSO-d$_6$) of Λ-**Ir**^{S,R}**3**.

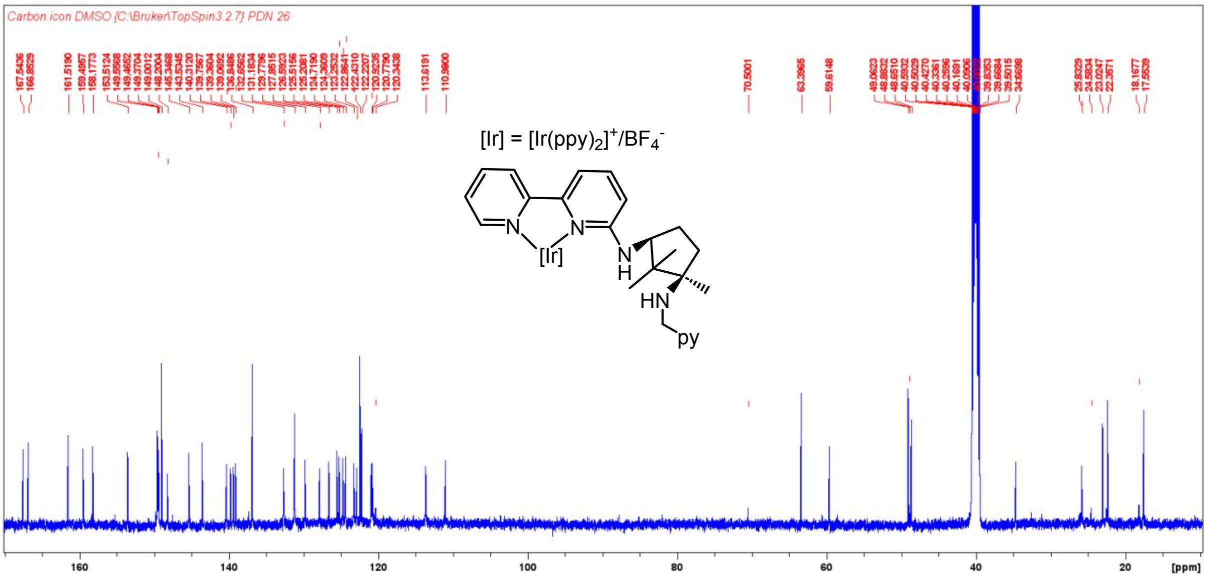

Figure S41. ^{13}C{^{1}H} NMR (125 MHz, DMSO-d$_6$) of Λ-**Ir**^{S,R}**3**.

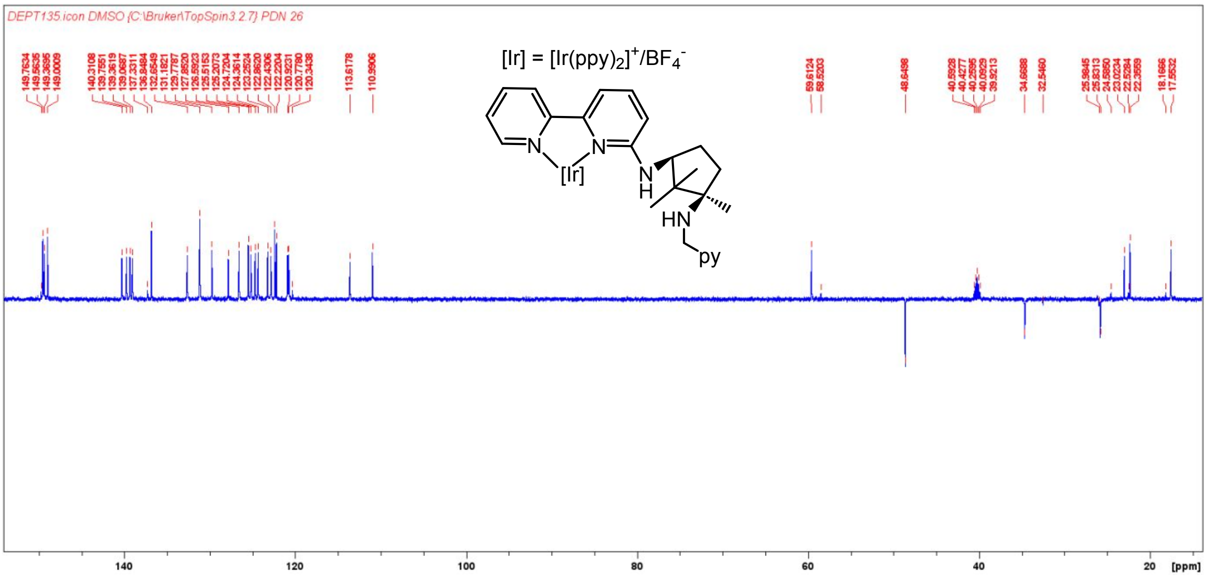

Figure S42. ^{13}C DEPT NMR (125 MHz, DMSO-d$_6$) of Λ-**Ir**^{S,R}**3**.

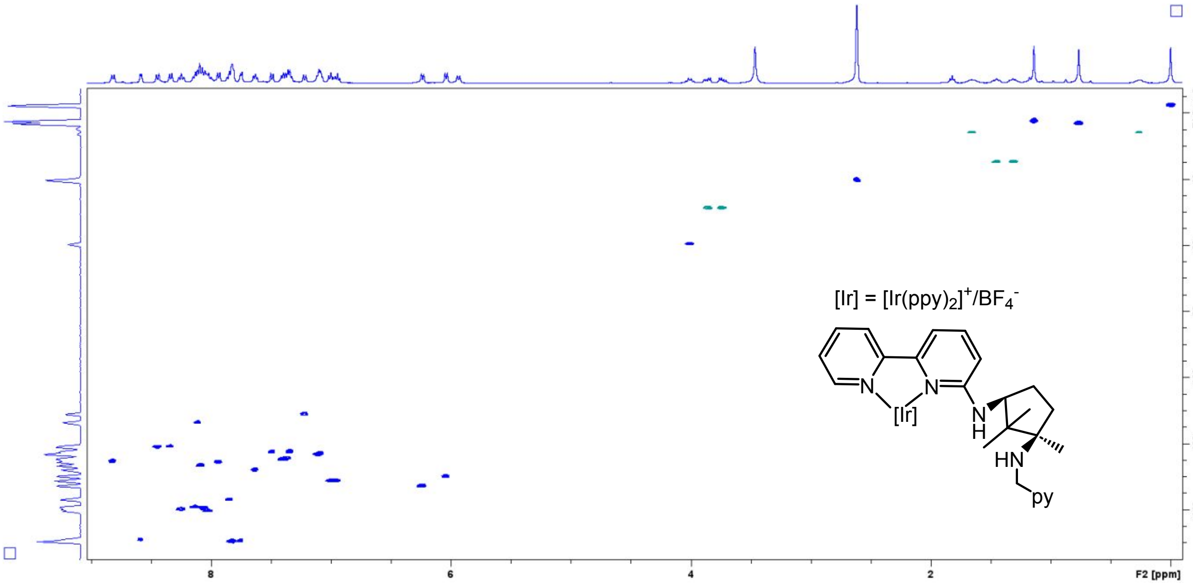

Figure S43. ^{1}H,^{13}C HSQC NMR (400 MHz, DMSO-d$_6$) of Λ-**Ir**^{S,R}**3**.

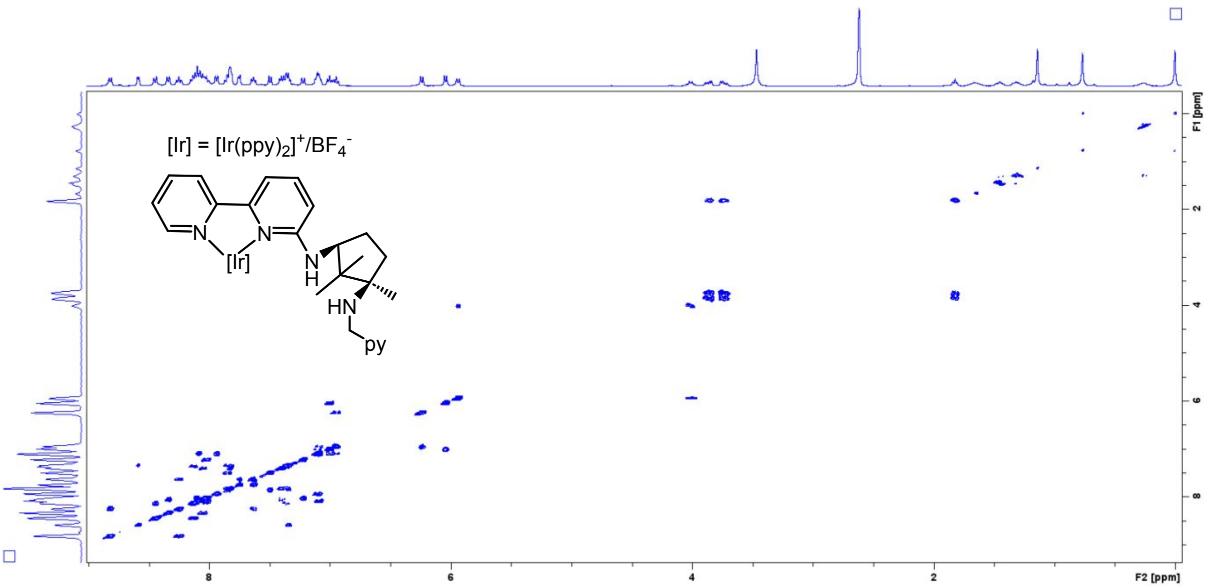

Figure S44. ^{1}H,^{1}H COSY NMR (400 MHz, DMSO-d_6) of Λ-**Ir**^{S,R}**3**.

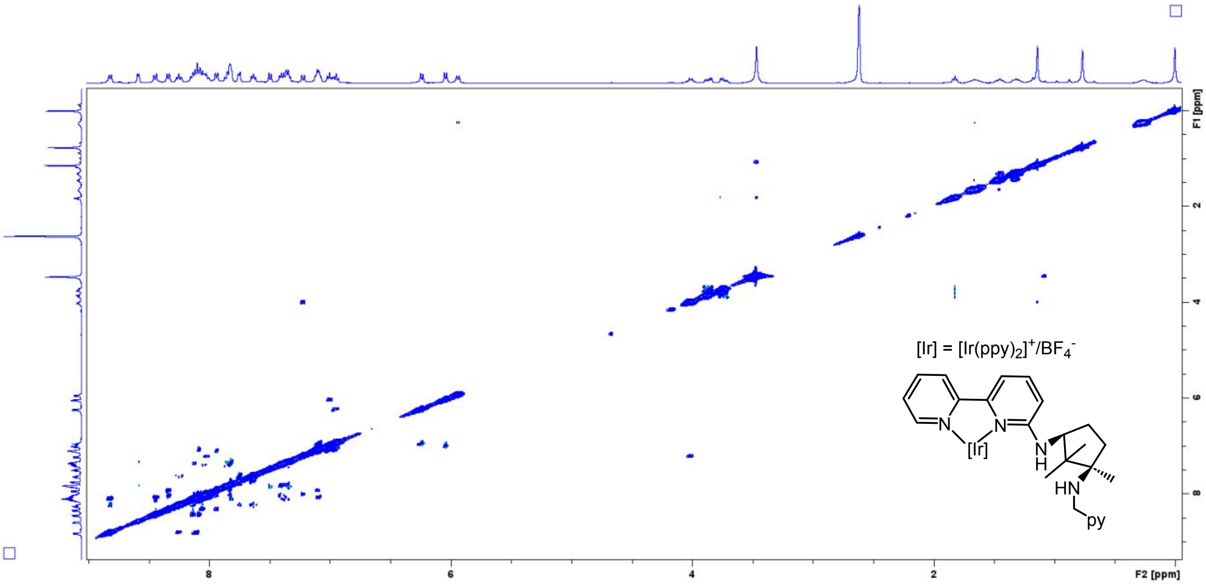

Figure S45. ^{1}H,^{1}H NOESY NMR (400 MHz, DMSO-d_6) of Λ-**Ir**^{S,R}**3**.

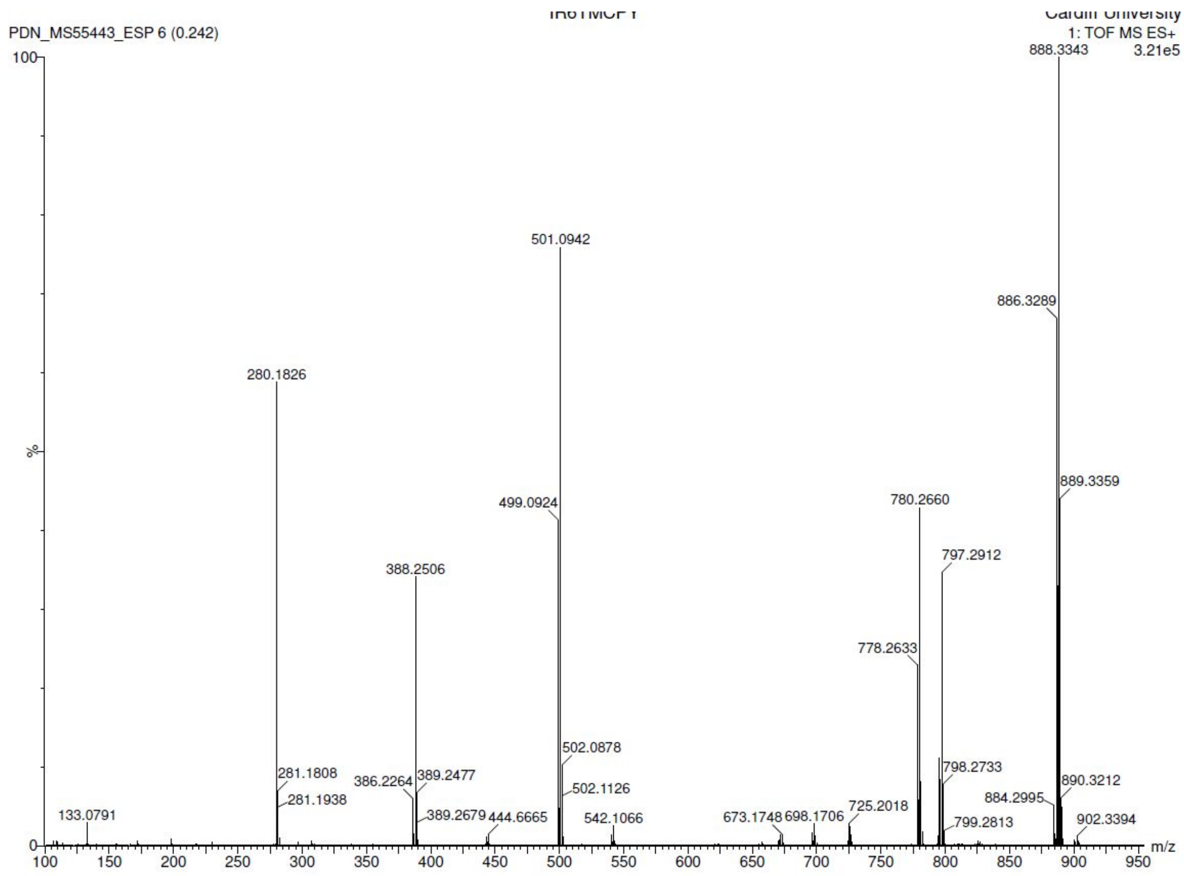

Mass	Calc. Mass	mDa	PPM	DBE	i-FIT	Norm	Conf(%)	Formula
Minimum:				-1.5				
Maximum:		5.0	5.0	100.0				
888.3343	888.3366	-2.3	-2.6	29.0	418.7	n/a	n/a	C46 H45 N7 193Ir

Figure S46. HRMS of Λ-**Ir**^{S,R}**3**.

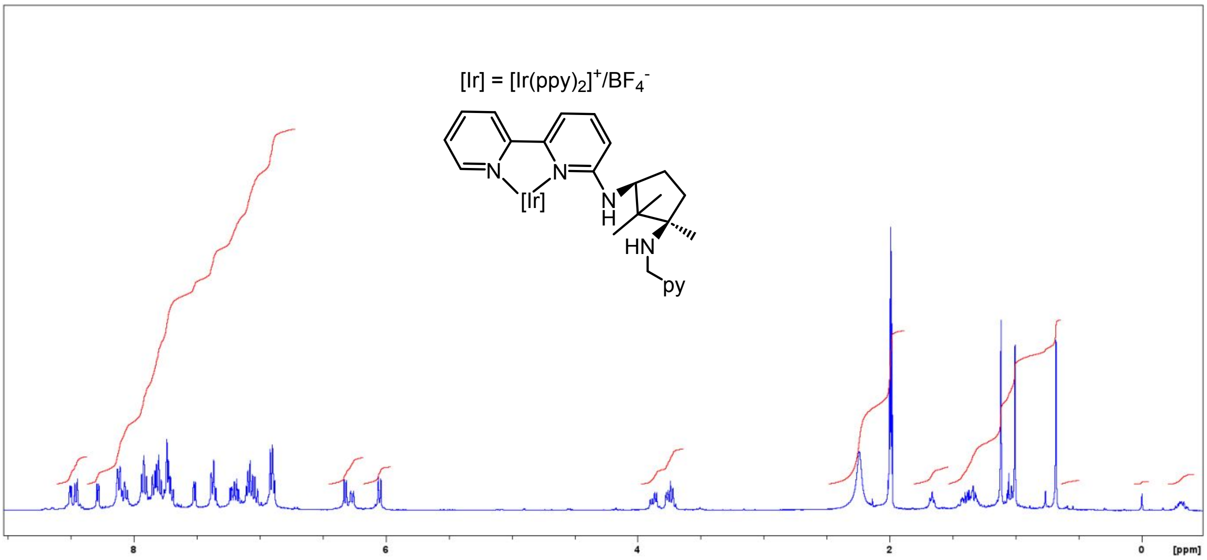

Figure S47. ^{1}H NMR (400 MHz, MeCN-d_3) of Δ-**Ir**^{S,R}**3**.

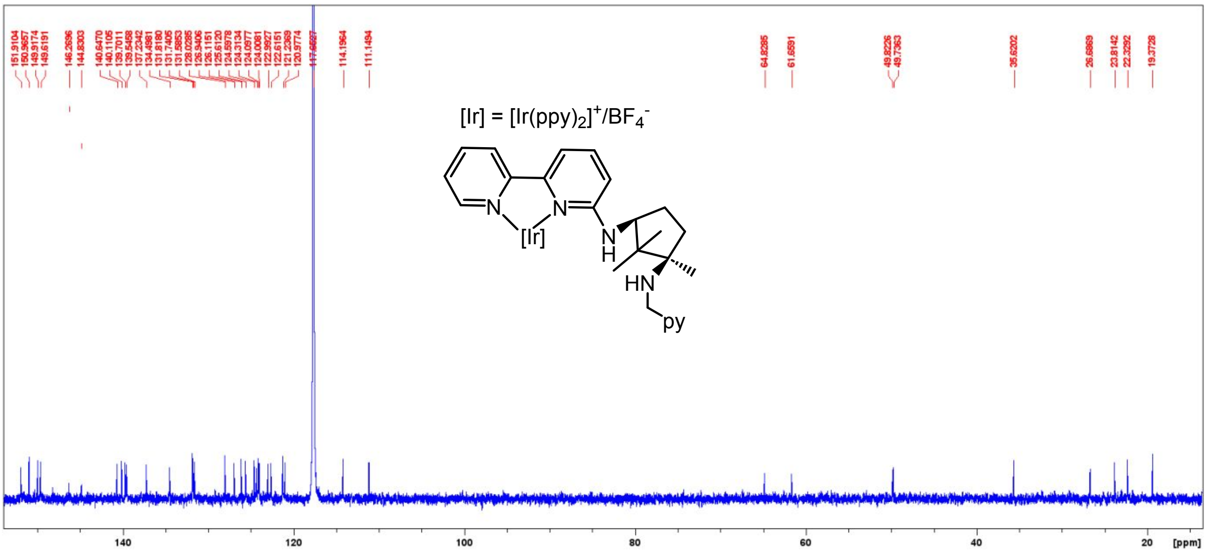

Figure S48. $^{13}C\{^{1}H\}$ NMR (100 MHz, MeCN-d$_3$) of Δ-**Ir**^{S,R}**3**.

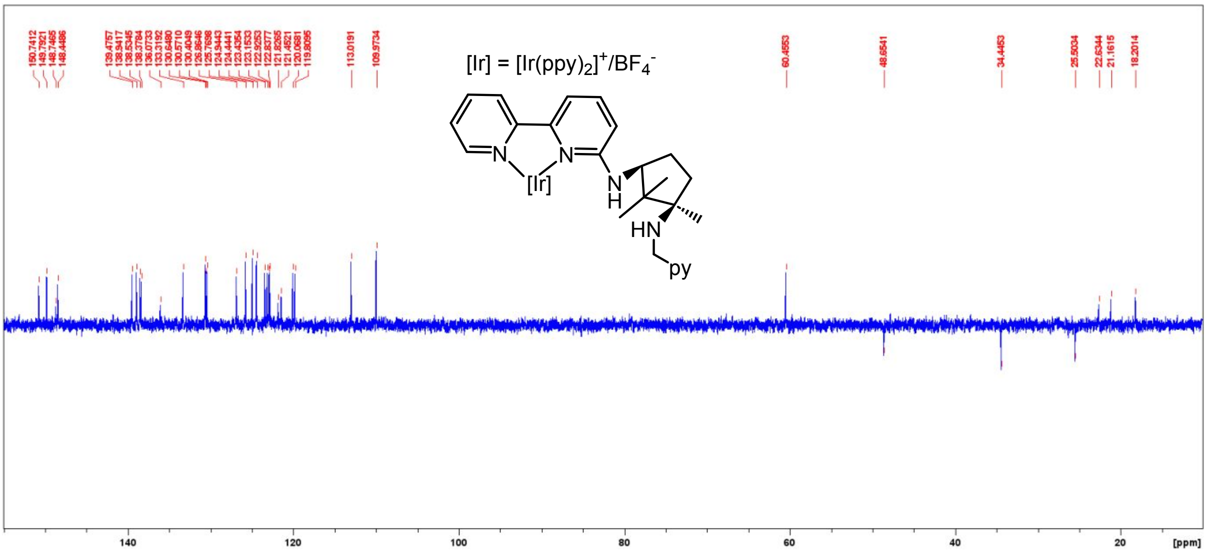

Figure S49. ^{13}C DEPT NMR (100 MHz, MeCN-d$_3$) of Δ-**Ir**^{S,R}**3**.

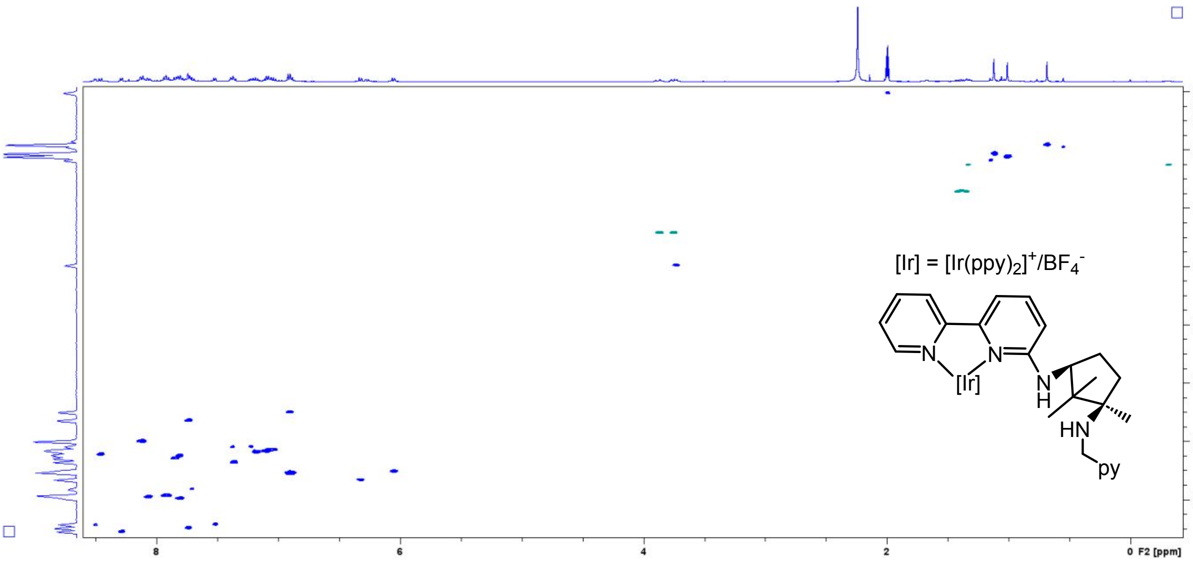

Figure S50. $^{1}H,^{13}C$ HSQC NMR (400 MHz, MeCN-d$_3$) of Δ-**Ir**^{S,R}**3**.

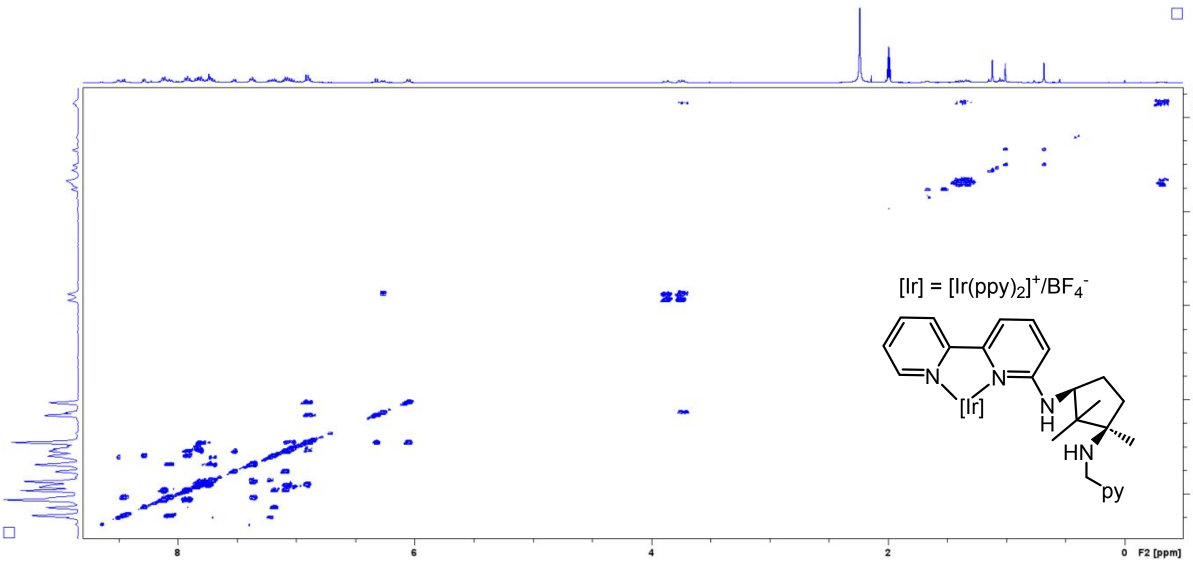

Figure S51. $^{1}H,^{1}H$ COSY NMR (400 MHz, MeCN-d_3) of Δ-**Ir**^{S,R}**3**.

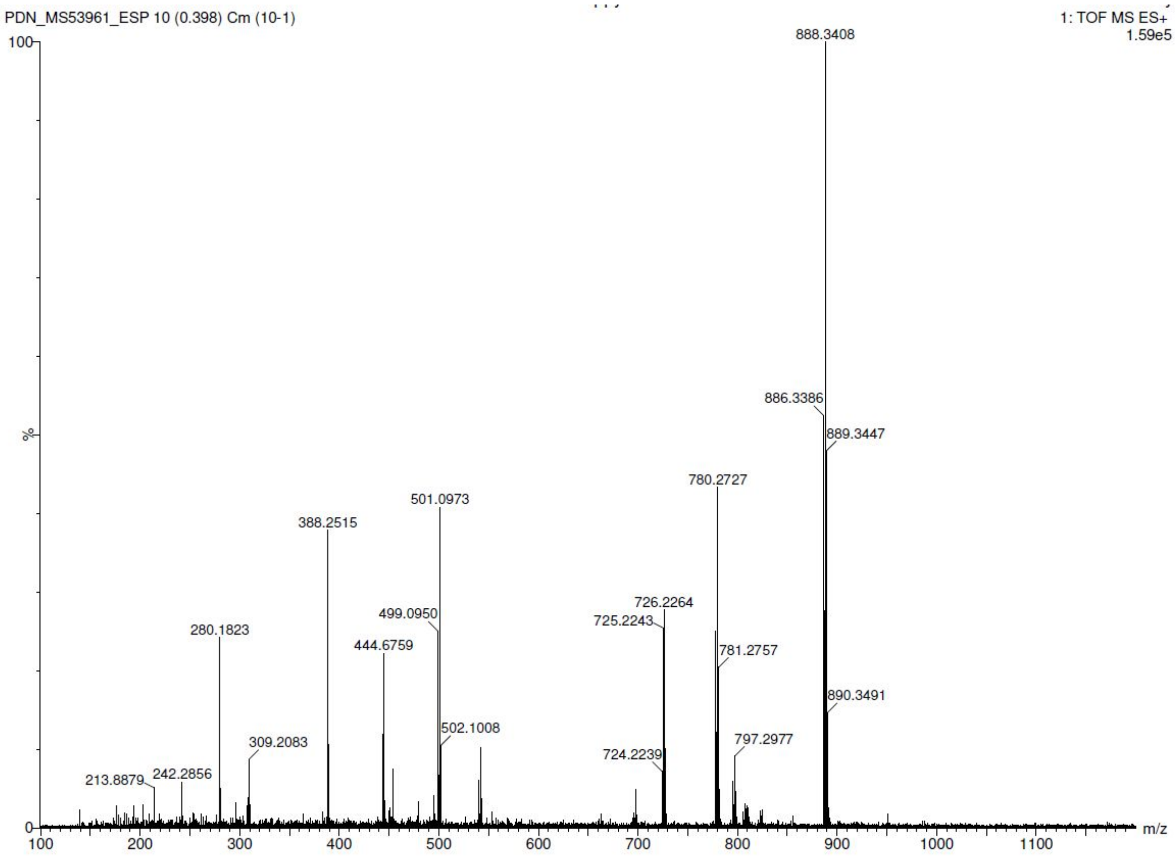

Minimum:				-1.5				
Maximum:		5.0	10.0	50.0				
Mass	Calc. Mass	mDa	PPM	DBE	i-FIT	Norm	Conf(%)	Formula
888.3408	888.3366	4.2	4.7	29.0	607.1	n/a	n/a	C46 H45 N7 Ir

Figure S52. HRMS of Δ-**Ir**^{S,R}**3**.

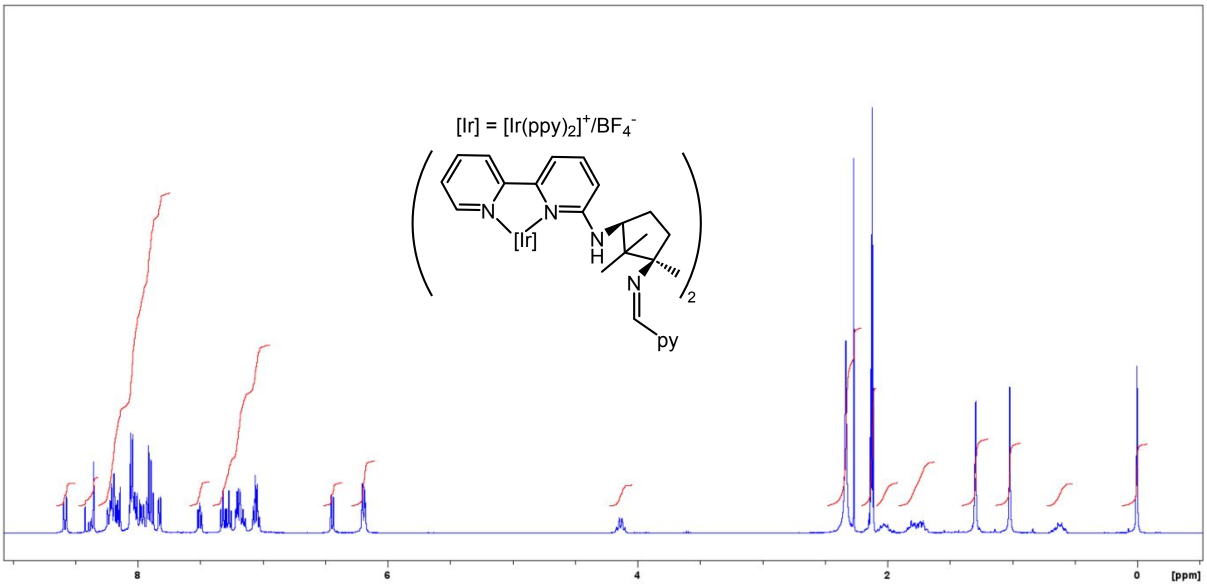

Figure S53. ^{1}H NMR (400 MHz, MeCN-d$_3$) of Λ-**Ir**^{S,R}**4**.

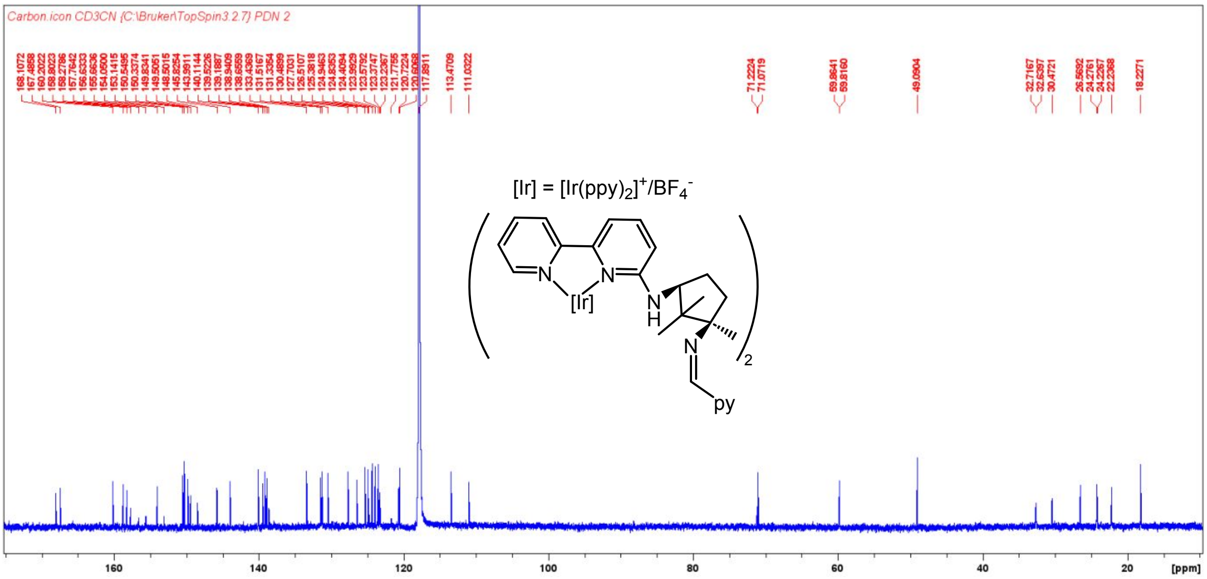

Figure S54. ^{13}C{^{1}H} NMR (100 MHz, MeCN-d$_3$) of Λ-**Ir**^{S,R}**4**.

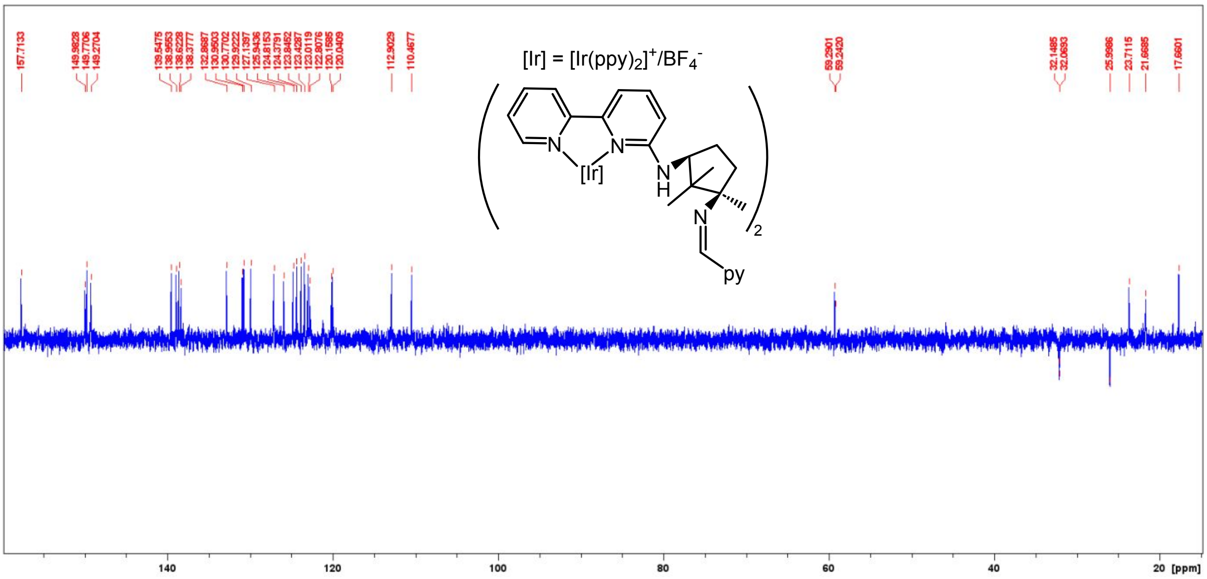

Figure S55. ^{13}C DEPT NMR (100 MHz, MeCN-d$_3$) of Λ-**Ir**^{S,R}**4**.

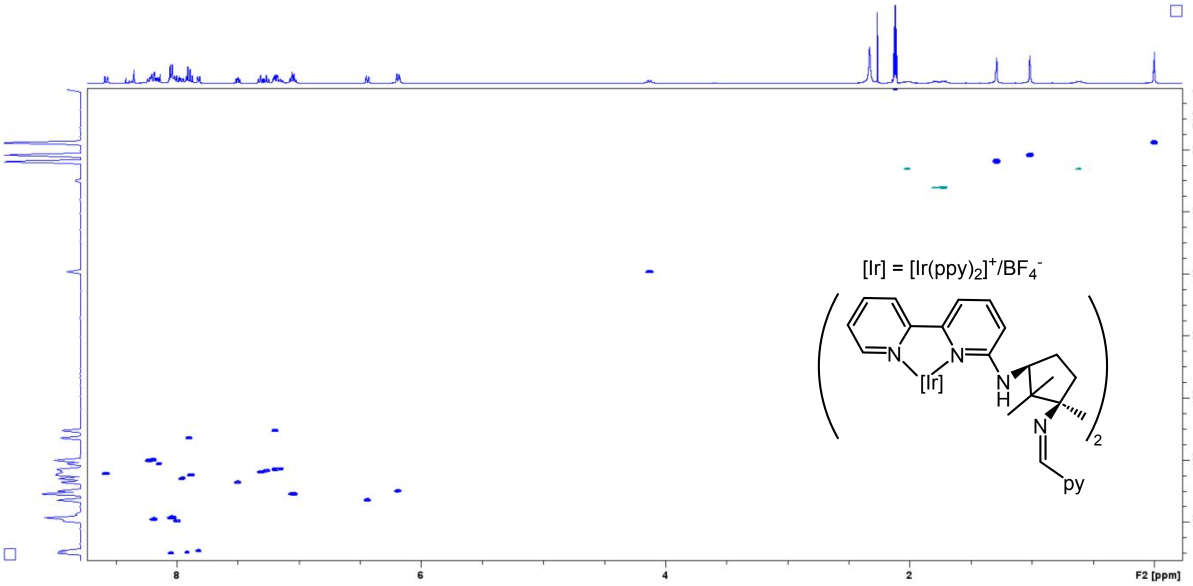

Figure S56. ^{1}H,^{13}C HSQC NMR (400 MHz, MeCN-d$_3$) of Λ-**Ir**^{S,R}**4**.

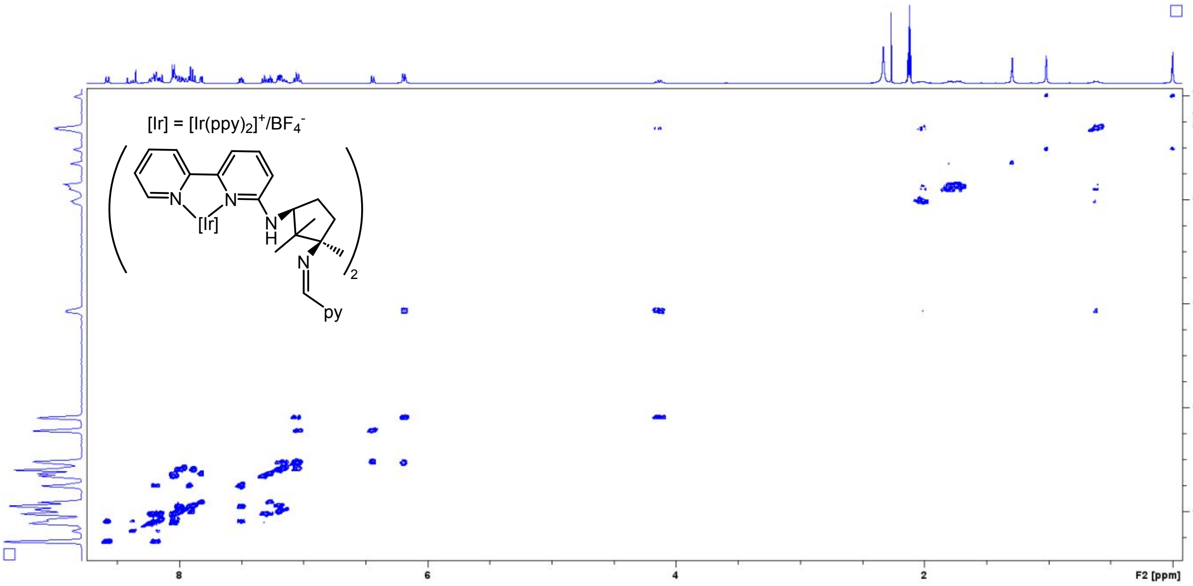

Figure S57. $^{1}H,^{1}H$ COSY NMR (400 MHz, MeCN-d_3) of Λ-**Ir**^{S,R}**4**.

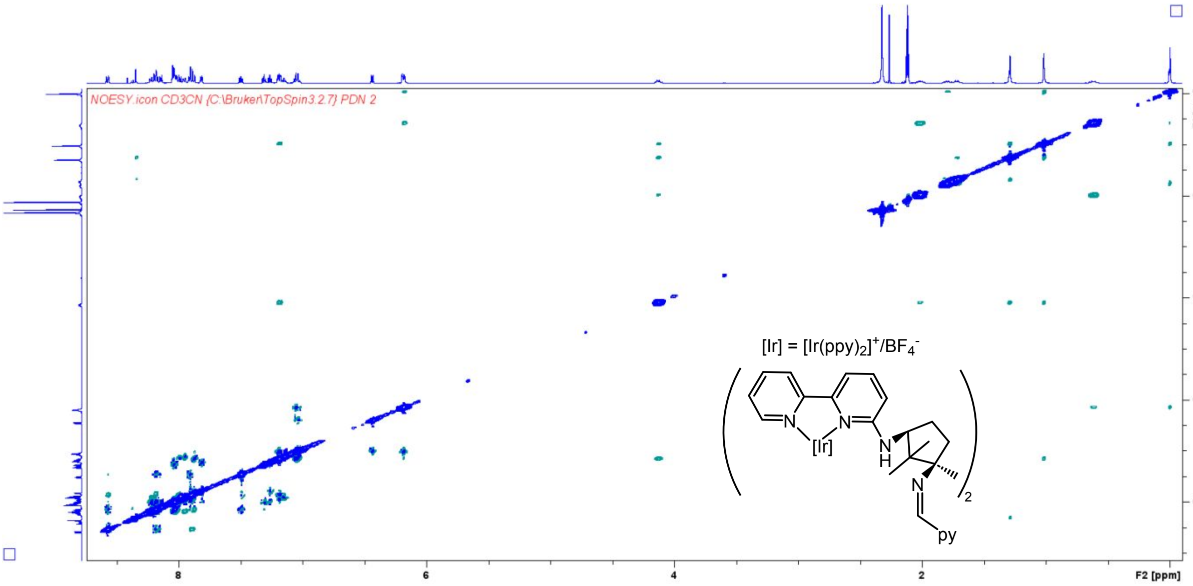

Figure S58. $^{1}H,^{1}H$ NOESY NMR (400 MHz, MeCN-d_3) of Λ-**Ir**^{S,R}**4**.

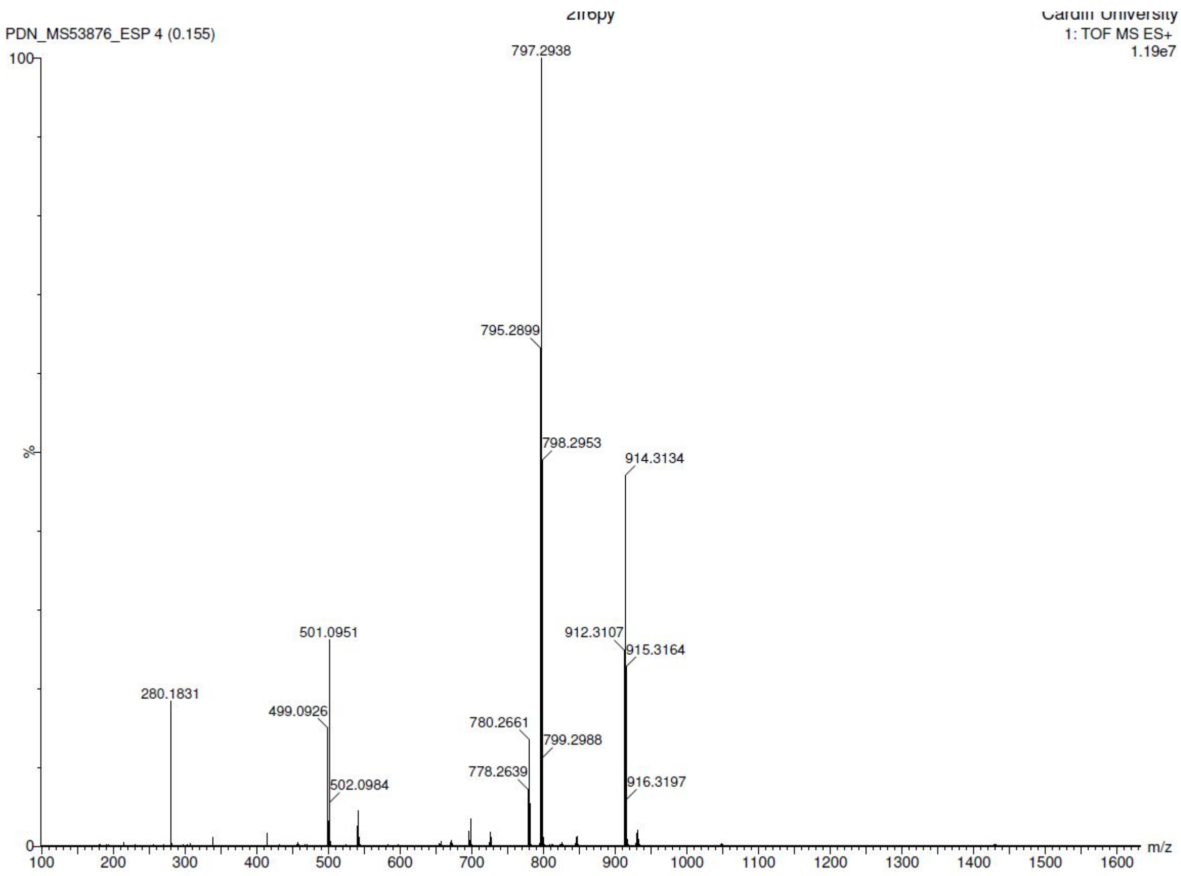

Figure S59. HRMS of Λ-**Ir**^{S,R}**4**. The peak at 914.3134 amu is for the cleaved fragment where one of the iridium complexes has been lost to leave the CHO group (C47H43N7OIr).

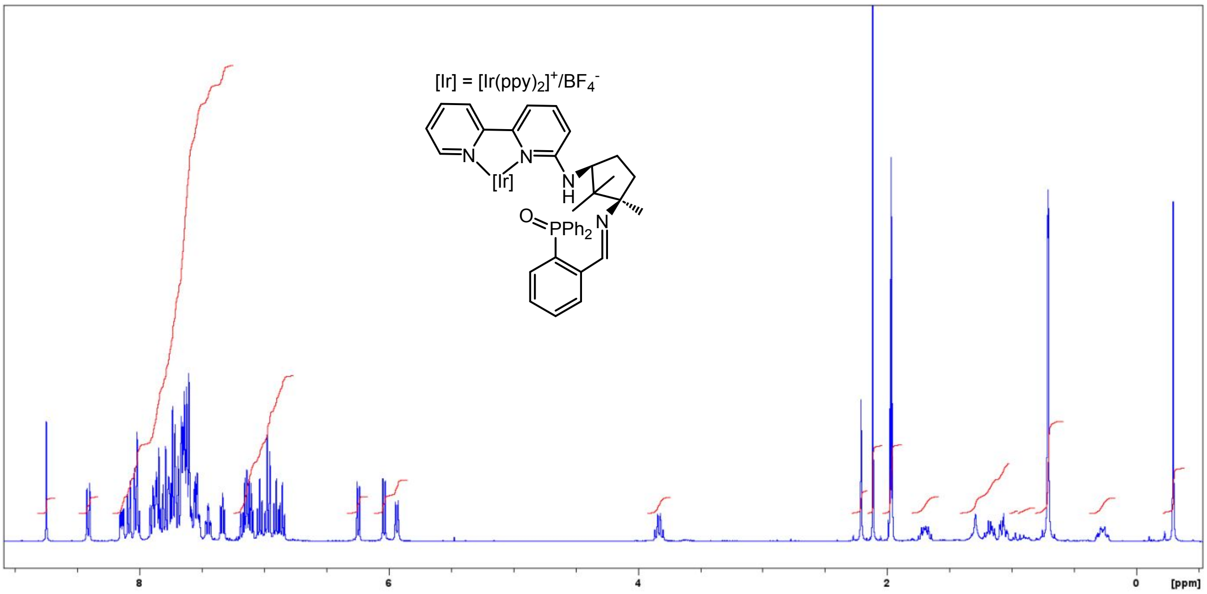

Figure S60. ^{1}H NMR (400 MHz, MeCN-d$_3$) of Λ-**Ir**^{S,R}**5**.

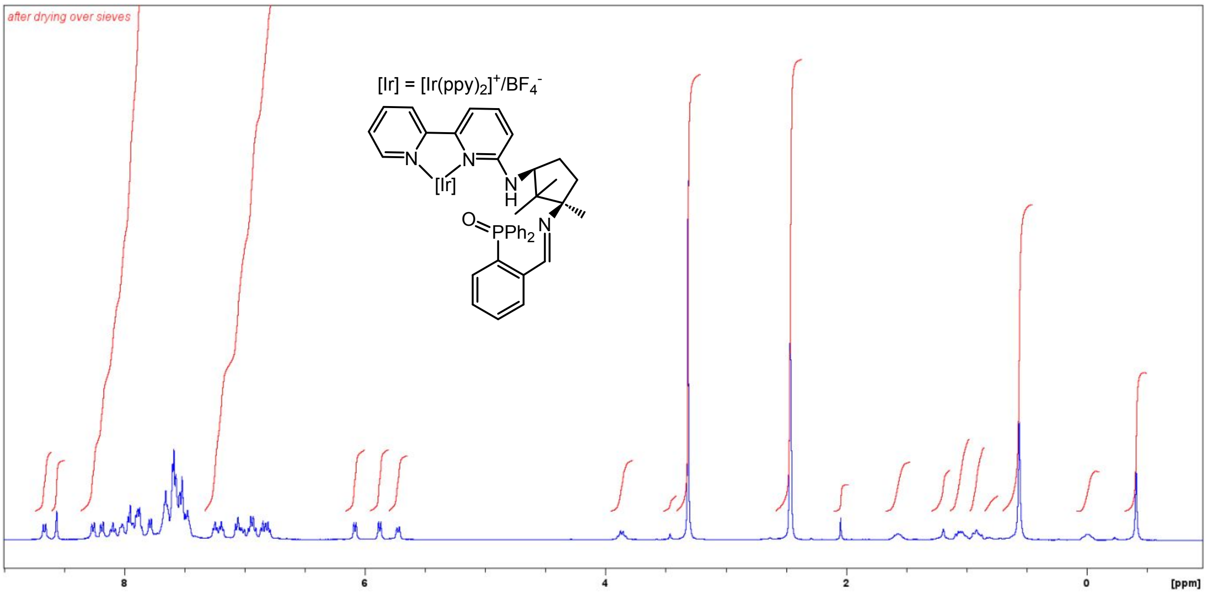

Figure S61. ^{1}H NMR (400 MHz, DMSO-d$_6$) of Λ-**Ir*^{S,R}***5**.

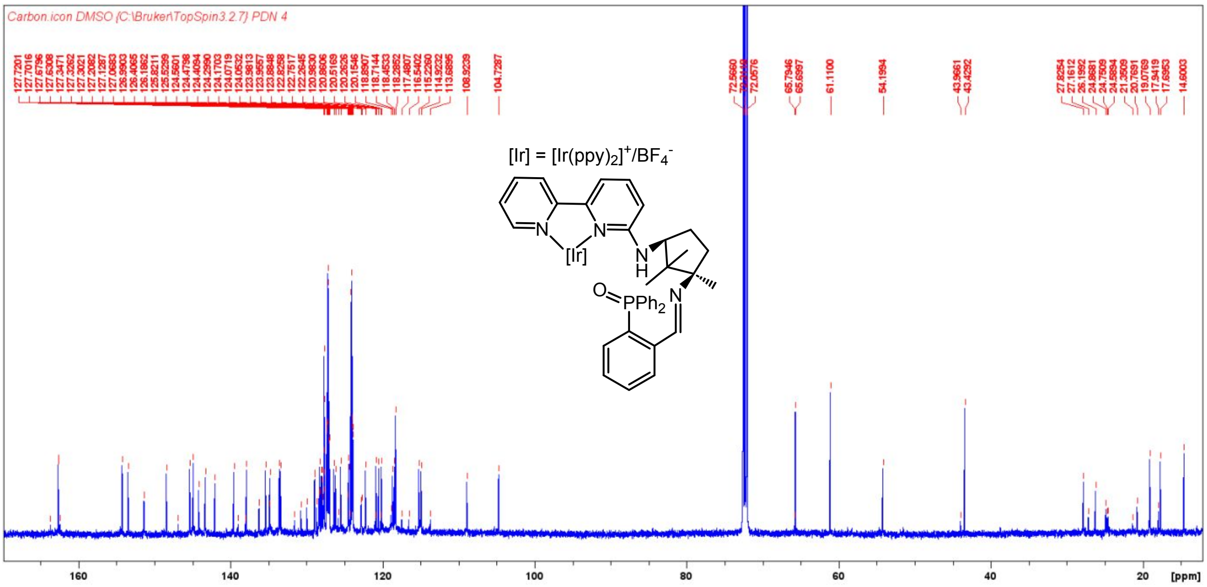

Figure S62. ^{13}C{^{1}H} NMR (100 MHz, DMSO-d$_6$) of Λ-**Ir*^{S,R}***5**.

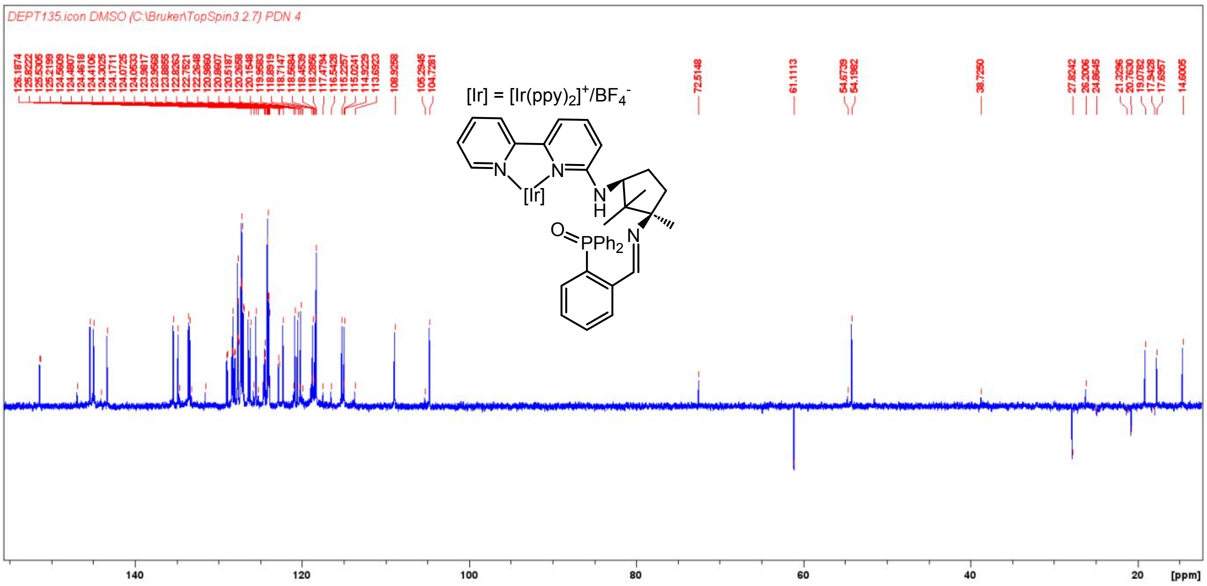

Figure S63. ^{13}C DEPT NMR (100 MHz, DMSO-d$_6$) of Λ-**Ir^{S,R}5**.

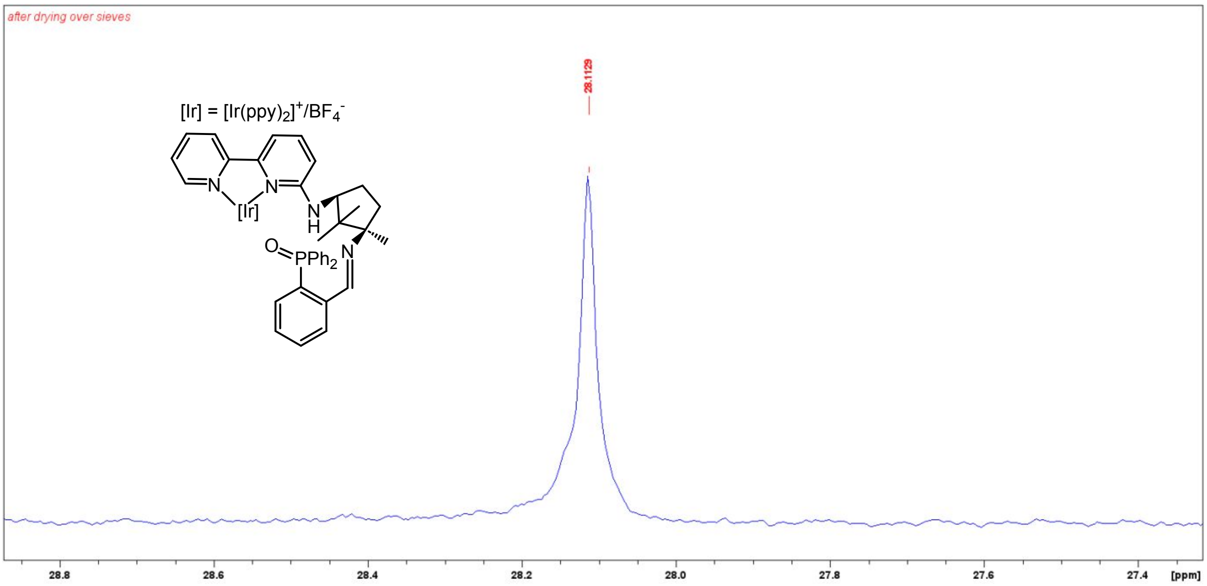

Figure S64. ^{31}P{^{1}H} NMR (162 MHz, DMSO-d$_6$) of Λ-**Ir^{S,R}5**.

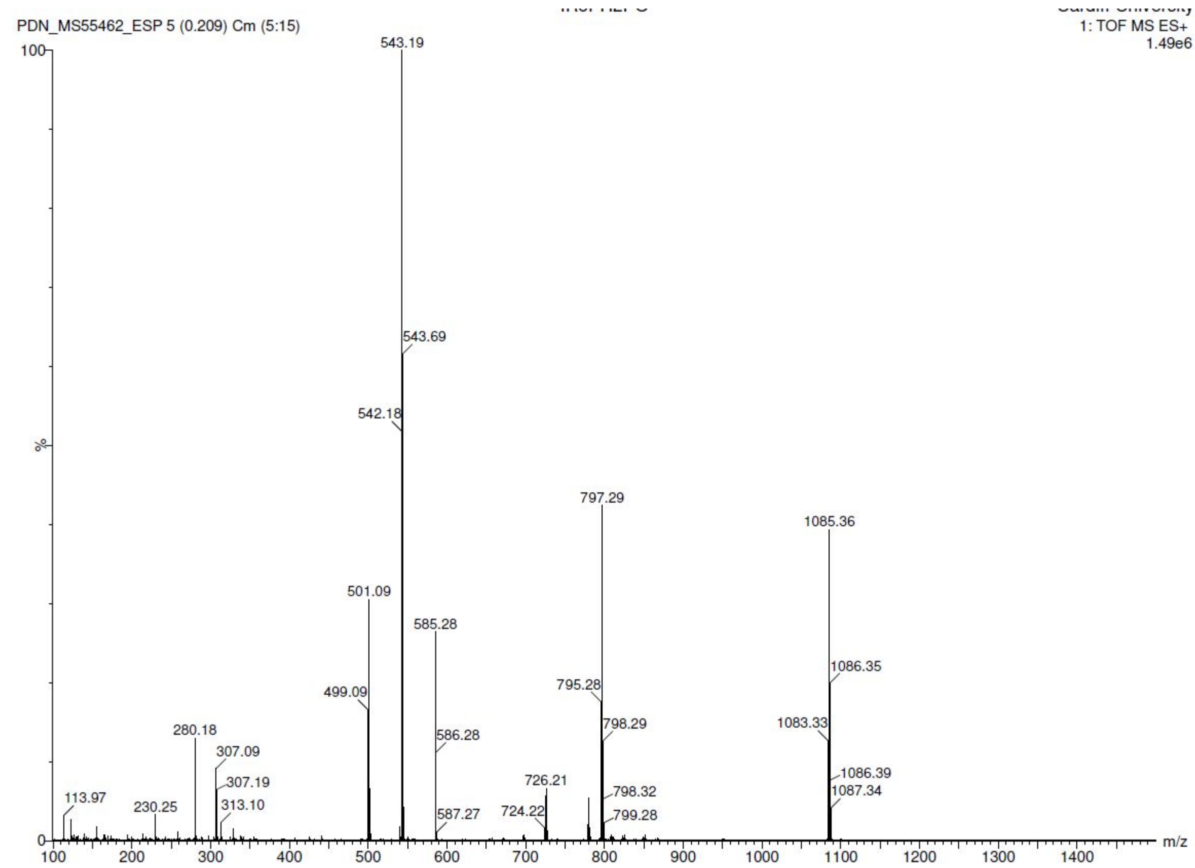

Figure S65. LRMS of Λ-**Ir**^{S,R}**5**. HRMS could not be obtained due to interference from a +1 amu species (presumably protonated).

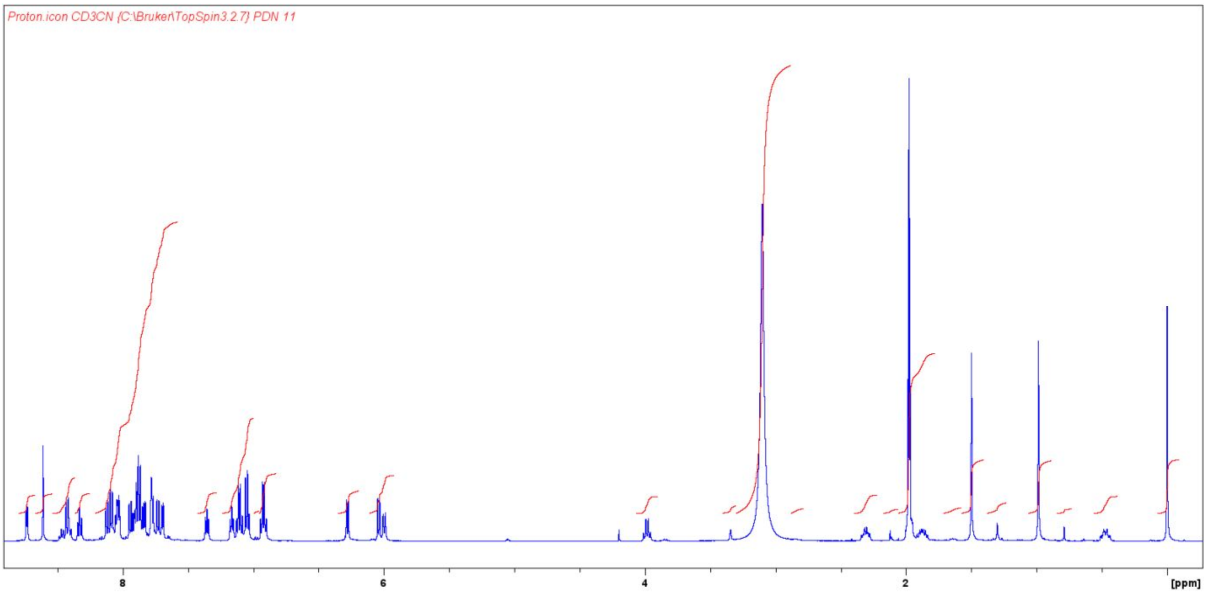

Figure S66. ^{1}H NMR (500 MHz, MeCN-d$_3$) of Λ-**Ir**^{S,R}**2-Zn**.

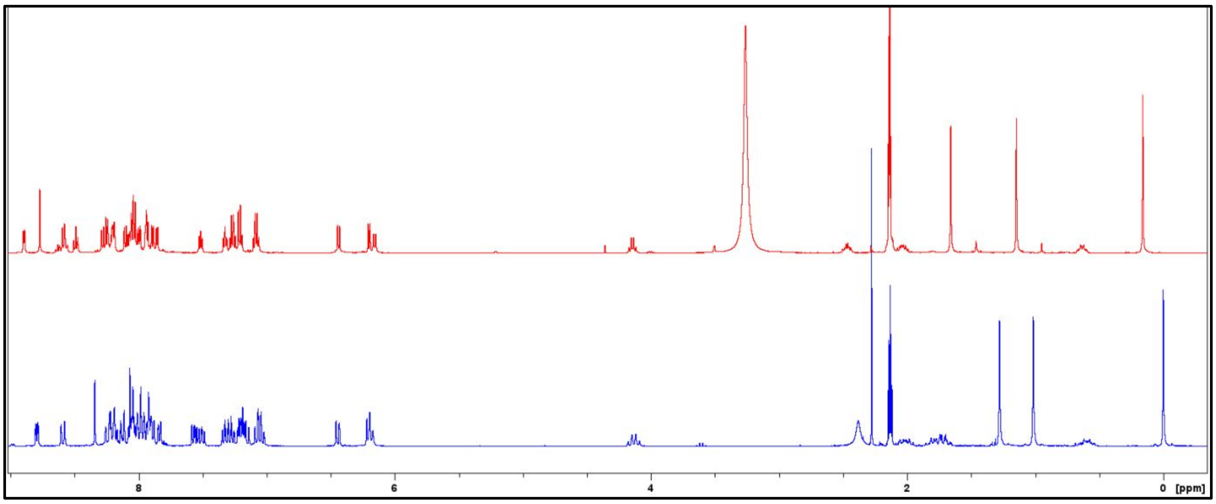

Figure S67. ^{1}H NMR (500 MHz, MeCN-d_3) of Λ-**Ir**^{S,R}**2** (bottom) and Λ-**Ir**^{S,R}**2-Zn** (top).

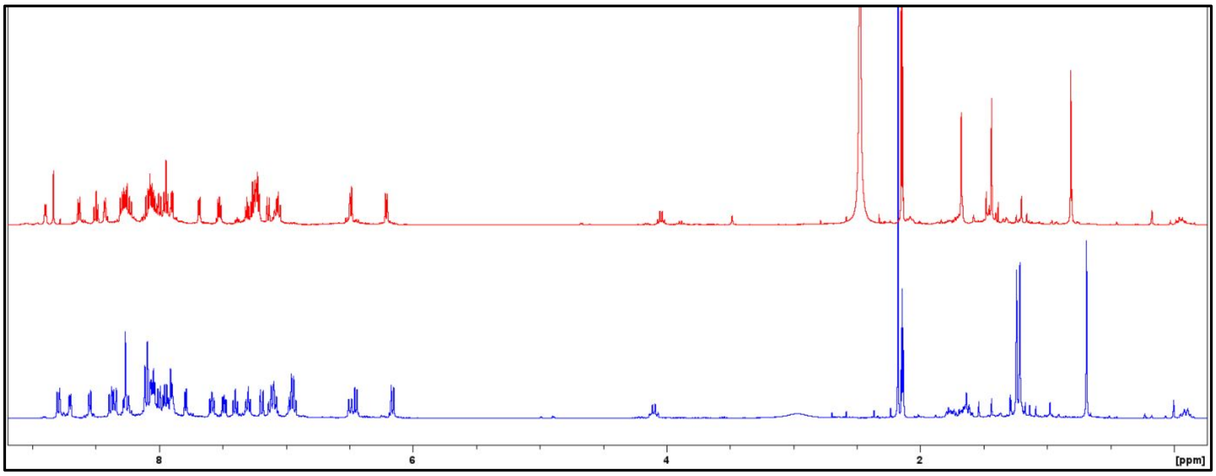

Figure S68. ^{1}H NMR (500 MHz, MeCN-d_3) of Δ-**Ir**^{S,R}**2** (bottom) and Δ-**Ir**^{S,R}**2-Zn** (top).

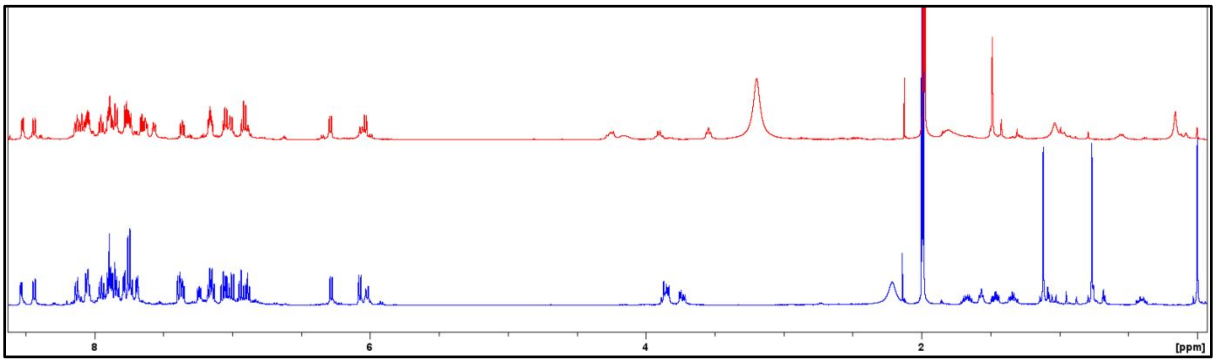

Figure S69. ^{1}H NMR (500 MHz, MeCN-d_3) of Λ-**Ir**^{S,R}**3** (bottom) and Λ-**Ir**^{S,R}**3-Zn** (top).

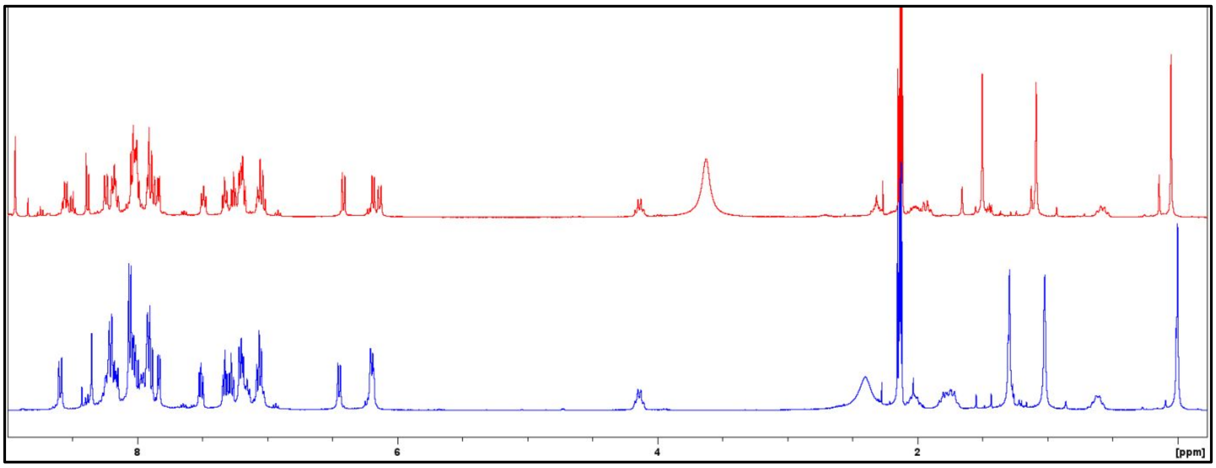

Figure S70. ^{1}H NMR (500 MHz, MeCN-d$_3$) of Λ-**Ir^{S,R}4** (bottom) and Λ-**Ir^{S,R}4-Zn** (top).

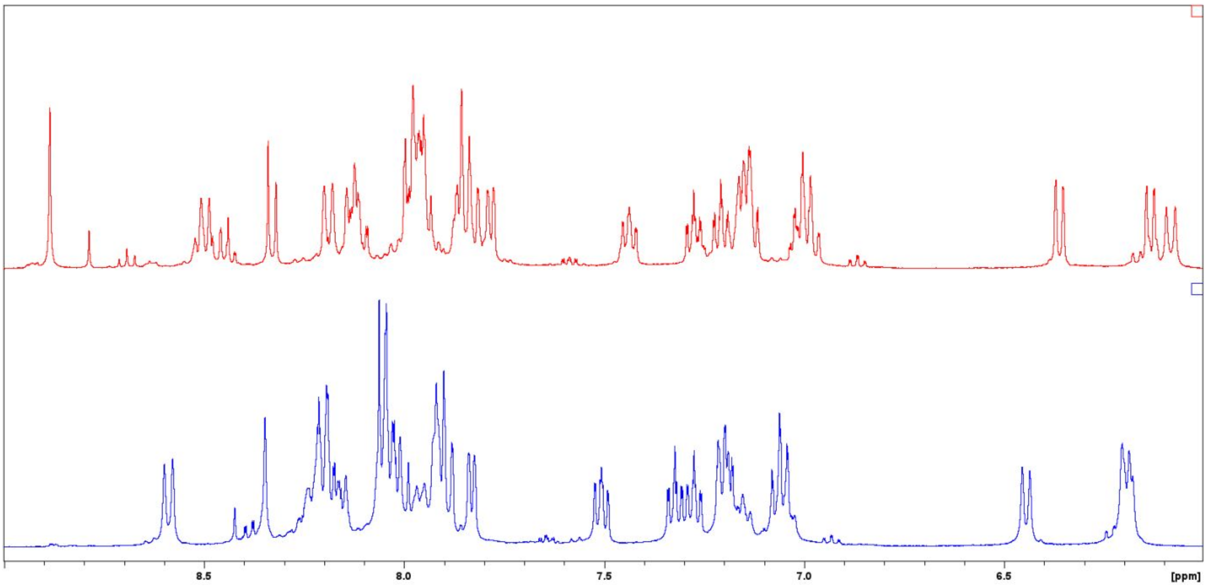

Figure S71. Expanded aromatic region of the ^{1}H NMR (500 MHz, MeCN-d$_3$) of Λ-**Ir^{S,R}4** (bottom) and Λ-**Ir^{S,R}4-Zn** (top).

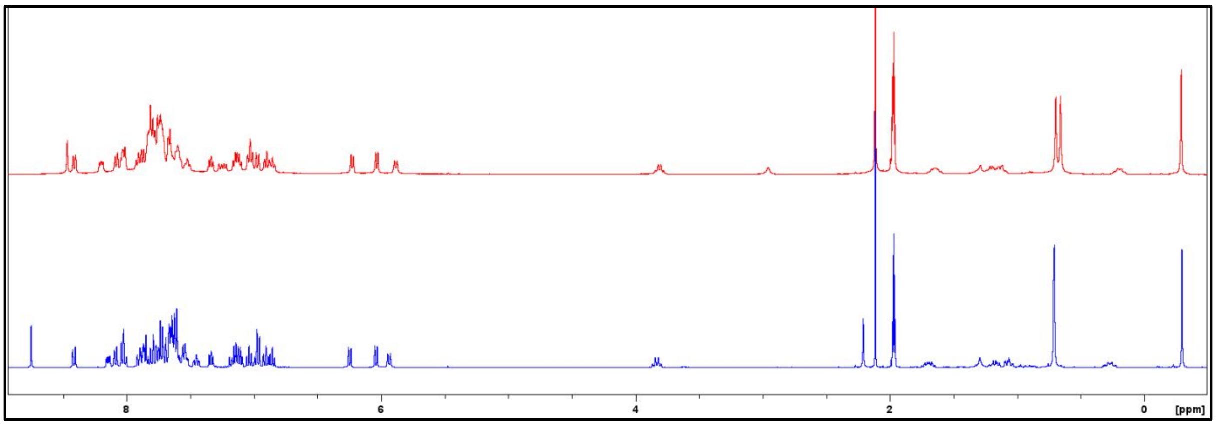

Figure S72. ^{1}H NMR (400 MHz, MeCN-d$_3$) of Λ-**Ir^{S,R}5** (bottom) and Λ-**Ir^{S,R}5-Zn** (top).

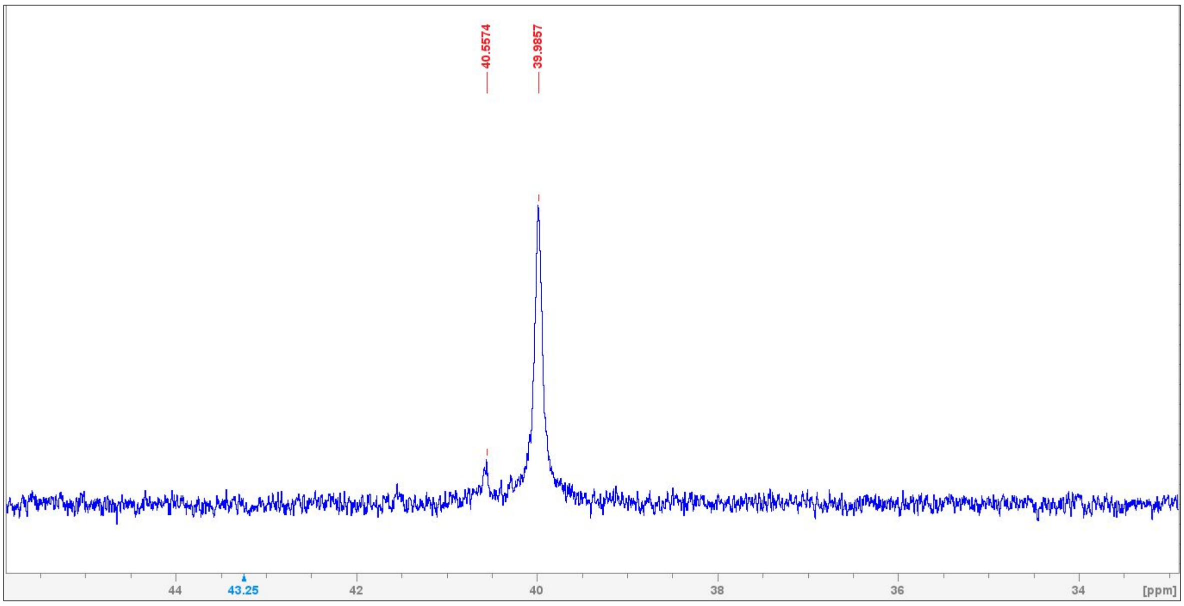

Figure S73. $^{31}P\{^{1}H\}$ NMR (162 MHz, DMSO-d_6) of Λ-**Ir^{S,R}5-Zn**.

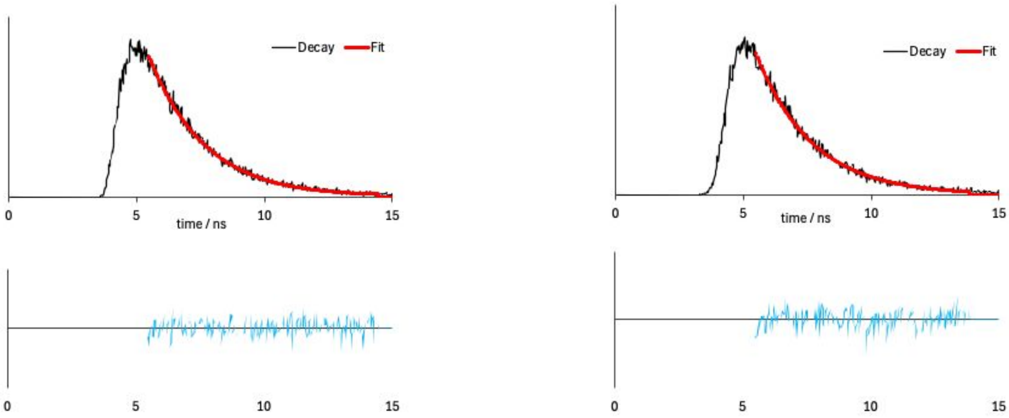

Figure S74. Fitted emission decay traces for Λ-**Ir^{S,R}1** (**left**, fitted parameters τ = 2.2 ns, χ^2 = 1.2) and Δ-**Ir^{S,R}1** (**right,** fitted parameters τ = 2.2 ns, χ^2 = 0.97) for the emission feature noted ca. 435 nm. The residual errors from the monoexponential fit are shown below.

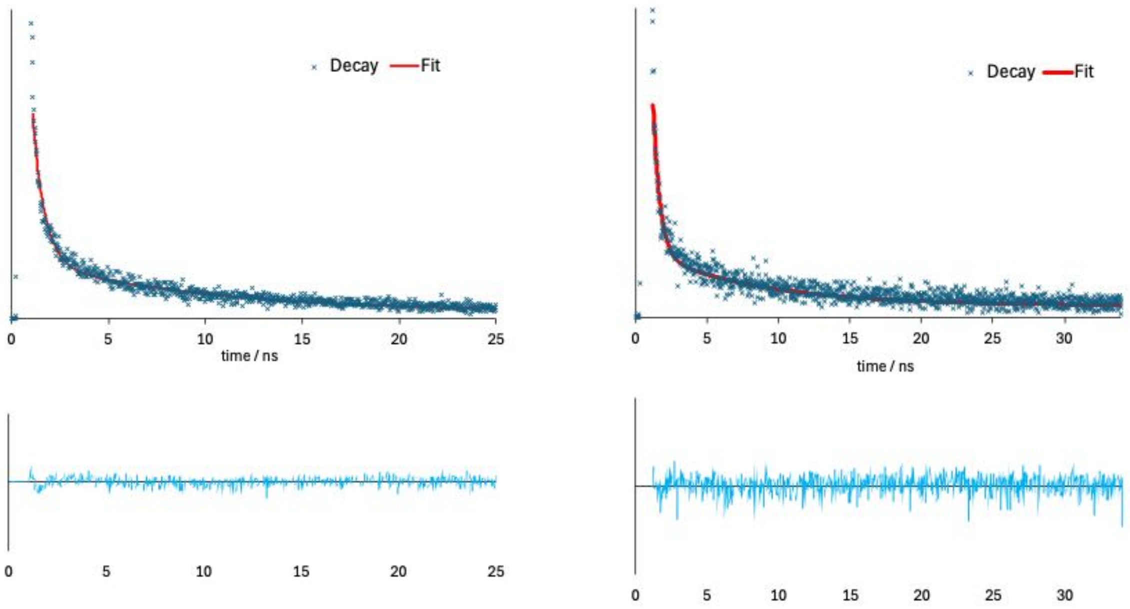

Figure S75. Fitted emission decay traces for Λ-**Ir**^{S,R}**1** (**left**, fitted parameters τ = 4.1 ns, and 65 ns (84%), χ^2 = 1.1) and Δ-**Ir**^{S,R}**1** (**right,** fitted parameters τ = 5.2 ns and 79 ns (83%), χ^2 = 1.1) for the emission feature noted ca. 550 nm. The residual errors from the biexponential fit are shown below.
